# Sphingosine analogue drug FTY720 targets I2PP2A/SET and mediates lung tumour suppression via activation of PP2A-RIPK1-dependent necroptosis

**DOI:** 10.1002/emmm.201201283

**Published:** 2012-11-25

**Authors:** Sahar A Saddoughi, Salih Gencer, Yuri K Peterson, Katherine E Ward, Archana Mukhopadhyay, Joshua Oaks, Jacek Bielawski, Zdzislaw M Szulc, Raquela J Thomas, Shanmugam P Selvam, Can E Senkal, Elizabeth Garrett-Mayer, Ryan M De Palma, Dzmitry Fedarovich, Angen Liu, Amyn A Habib, Robert V Stahelin, Danilo Perrotti, Besim Ogretmen

**Affiliations:** 1 Department of Biochemistry and Molecular Biology, Medical University of South CarolinaCharleston, SC, USA; 2 Hollings Cancer Center, Medical University of South CarolinaCharleston, SC, USA; 3 Department of Pharmaceutical Sciences, Medical University of South CarolinaCharleston, SC, USA; 4 Department of Chemistry and Biochemistry and the Mike and Josie Harper Center for Cancer Research, University of Notre DameNotre Dame, IN, USA; 5 Human Cancer Genetics Program, Department of Molecular Virology, Immunology, and Medical Genetics, The Ohio State UniversityColumbus, OH, USA; 6 Department of Neurology and Neurotherapeutics, University of Texas Southwestern Medical CenterDallas, TX, USA; 7 North Texas Veterans Administration Medical CenterDallas, TX, USA; 8 Department of Biochemistry and Molecular Biology, Indiana University School of MedicineSouth Bend, IN, USA

**Keywords:** ceramide, FTY720, sphingolipids, sphingosine, sphingosine kinase 2

## Abstract

Mechanisms that alter protein phosphatase 2A (PP2A)-dependent lung tumour suppression via the I2PP2A/SET oncoprotein are unknown. We show here that the tumour suppressor ceramide binds I2PP2A/SET selectively in the nucleus and including its K209 and Y122 residues as determined by molecular modelling/simulations and site-directed mutagenesis. Because I2PP2A/SET was found overexpressed, whereas ceramide was downregulated in lung tumours, a sphingolipid analogue drug, FTY720, was identified to mimick ceramide for binding and targeting I2PP2A/SET, leading to PP2A reactivation, lung cancer cell death, and tumour suppression *in vivo*. Accordingly, while molecular targeting of I2PP2A/SET by stable knockdown prevented further tumour suppression by FTY720, reconstitution of WT-I2PP2A/SET expression restored this process. Mechanistically, targeting I2PP2A/SET by FTY720 mediated PP2A/RIPK1-dependent programmed necrosis (necroptosis), but not by apoptosis. The RIPK1 inhibitor necrostatin and knockdown or genetic loss of RIPK1 prevented growth inhibition by FTY720. Expression of WT- or death-domain-deleted (DDD)-RIPK1, but not the kinase-domain-deleted (KDD)-RIPK1, restored FTY720-mediated necroptosis in RIPK1^−/−^ MEFs. Thus, these data suggest that targeting I2PP2A/SET by FTY720 suppresses lung tumour growth, at least in part, via PP2A activation and necroptosis mediated by the kinase domain of RIPK1.

## INTRODUCTION

Protein phosphatase 2A (PP2A) is a tumour suppressor enzyme (Eichhorn et al, 2009; Westermarck & Hahn, 2008) involved in the regulation of oncoproteins, such as c-Myc (Yeh et al, 2004) and Bcr-Abl (Salas et al, 2011), in various cancers including lung cancers and CML, respectively. In addition to inactivating mutations of PP2A (Westermarck & Hahn, 2008), there are biological inhibitors, such as inhibitor 2 of PP2A (I2PP2A/SET oncoprotein), which directly binds and modulates PP2A function (Li et al, 1996). However, mechanisms involved in the regulation of PP2A-I2PP2A/SET interaction for controlling PP2A-dependent tumour suppression in human cancer cells have been largely unknown.

Ceramide activates PP2A (Chalfant et al, 1999; Ogretmen and Hannun, 2004) in part via directly binding I2PP2A/SET, which relieves PP2A from the inhibitor, thus increasing PP2A activity (Mukhopadhyay et al, 2009). However, whether endogenous ceramides, which are generated *de novo* by ceramide synthases 1–6 (CerS1–6) (Pewzner-Jung et al, 2006), also bind I2PP2A/SET remain unknown.

FTY720 (2-amino-2-[2-(4-octylphenyl)ethyl]propane-1,3-diol; Fingolimod, Novartis), a synthetic sphingosine analogue of myriocin, regulates sphingosine-1-phosphate receptor signalling and suppresses autoimmunity upon its phosphorylation by sphingosine kinase-2 (SK-2) (Billich et al, 2003; Paugh et al, 2003). FTY720 has been approved by the FDA for treatment of patients with refractory multiple sclerosis (Cohen et al, 2010) and has been shown to exert anti-cancer functions against CML (Neviani et al, 2007) and GISTs via activation of PP2A (Roberts et al, 2010) by an unknown mechanism.

Although induction of apoptosis via Bax/Bak-dependent caspase activation is important for anti-cancer therapeutics, necroptosis via activation of RIPK1 (Thon et al, 2005) might also play a role in this process (Bonapace et al, 2010; Degterev et al, 2008). However, involvement of RIPK1-induced necroptosis in lung tumour suppression via I2PP2A/SET targeting by FTY720 has not been reported previously. Here, using molecular modelling/simulations and site-directed mutagenesis, we determined the structural details of endogenous ceramide and I2PP2A/SET binding. Ceramide was found altered in lung tumours, whereas I2PP2A/SET was highly expressed in the majority of these tissues. We then examined whether tumour suppressive ceramide signalling can be restored by the sphingosine analogue drug FTY720. We discovered that FTY720 directly binds I2PP2A/SET, leading to PP2A activation and cell death. We also defined the mechanism by which FTY720 mediates cell death via induction of PP2A/RIPK1-dependent necroptosis, leading to lung tumour suppression.

## RESULTS

### Structural modelling of I2PP2A/SET-ceramide binding

**Figure 1:**
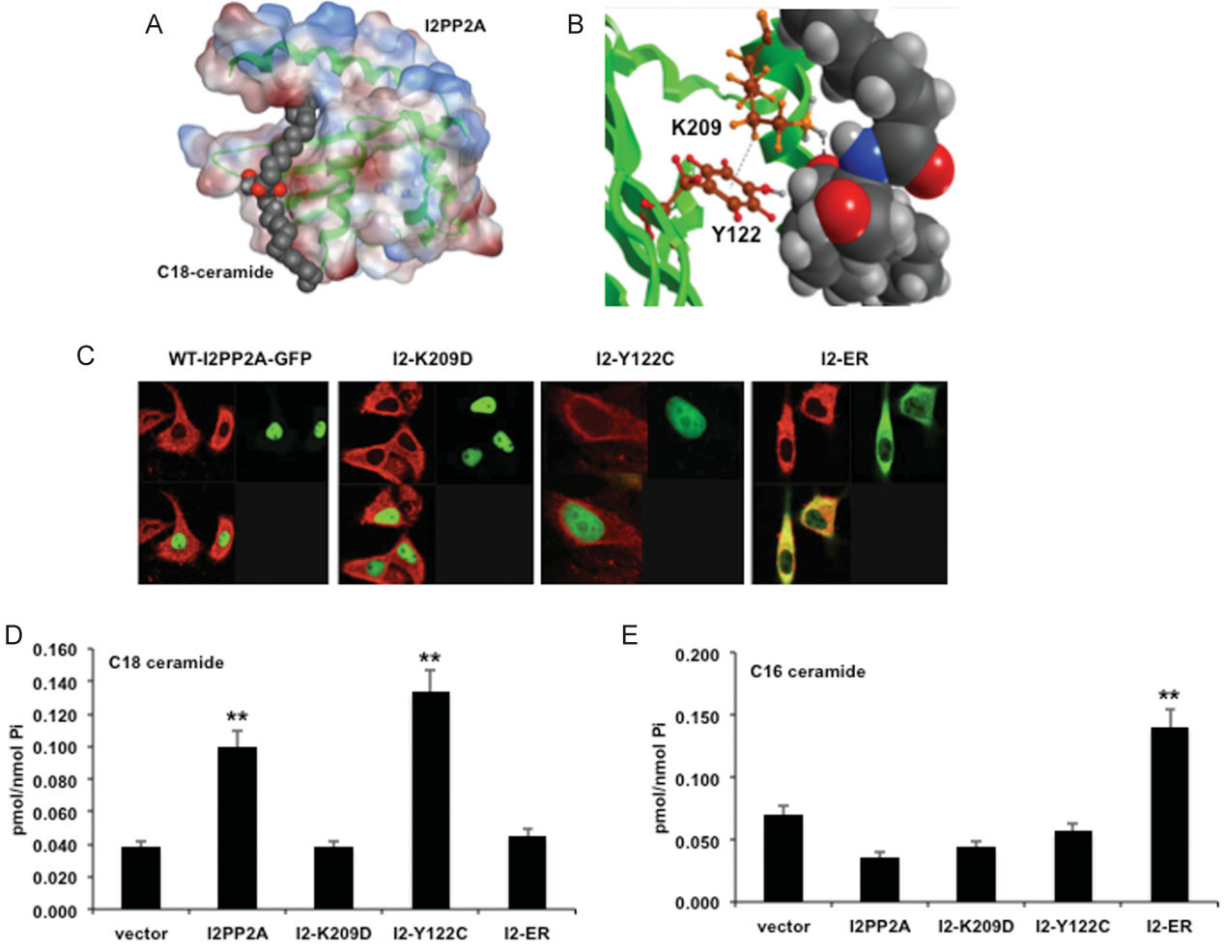
C_18_-ceramide selectively binds nuclear I2PP2A/SET through K209 and Y122 residues **A.** I2PP2A/SET is docked to the hydrophobic domain of I2PP2A/SET, containing two anti-parallel beta-sheets and an alpha helix. Hydrophobic and hydrophilic residues are shown in red and blue, respectively.**B.** Molecular docking simulations of C_18_-ceramide to I2PP2A/SET identifies K209 residue interacting with the 1-OH group of ceramide. Close up examination of K209 residue of I2PP2A/SET shows a potential π-cationic interaction between K209 (cationic) and Y122 (π-arene) that may form a gate to the hydrophobic pocket of I2PP2A/SET.**C.** Sub-cellular localization of WT- and K209D-, Y122C- and ER-I2PP2A/SET-GFP were examined by confocal microscopy. Co-localization of GFP and calnexin (red) were determined for ER detection (yellow).**D,E.** Binding of WT-, K209D-, Y122C- and ER-I2PP2A/SET-GFP to endogenous C_18_-ceramide (**D**) *versus* C_16_-ceramide (**E**) was measured by LC/MS/MS. Samples were normalized to inorganic phosphate (Pi). Error bars show s.d., and **p < 0.01 were considered significant.

To uncover the structural details of I2PP2A/SET-ceramide binding, molecular modelling/simulations were performed using the crystal structure of I2PP2A/SET (Muto et al, 2007) and C_18_-ceramide as a probe (Fig 1A). Our previous study showed that a single mutation with K209D conversion significantly inhibited the binding of I2PP2A/SET to ceramide both *in vitro* and in A549 cells (Mukhopadhyay et al, 2009). Accordingly, one of the prominent docking sites of I2PP2A/SET for ceramide binding included the K209 residue (Fig 1A and B, and Supporting Information Fig S1), which interacts with the primary hydroxyl group of ceramide possibly via charge attraction (Fig 1B, and Supporting Information Fig S1). The model also suggested that the K209 directly interacts with the Y122 residue via a hydrophobic–ionic (cation/π-arene) interaction (Fig 1B), possibly playing a role as a gate for regulating the access of ceramide to the hydrophobic pocket.

### Binding preferences of I2PP2A/SET with endogenous ceramides in A549 cells

To determine the possible involvement of K209 and Y122 residues in ceramide binding, we generated K209D and Y122C mutants of I2PP2A/SET. The K209D point mutation introduces negatively charged aspartic acid in place of lysine, which is predicted to cause electron repulsion with the 1-OH of ceramide, thus decreasing ceramide binding. In addition, the interaction between K209 and Y122 might form a cation/π-arene interaction. Thus, the conversion of Y122 to C122 should eliminate the interaction and open the gate for the hydrophobic pocket, which should increase ceramide binding. To examine the preference of Y122C or K209D mutations with endogenous ceramide binding, WT-, K209D- and Y122C-I2PP2A/SET-GFP proteins were expressed in A549 cells and accumulated in the nucleus like endogenous I2PP2A (Mukhopadhyay et al, 2009) (Fig 1C). Using anti-GFP columns, I2PP2A/SET-GFP was pulled down and protein-bound ceramides were measured after lipid extraction from the pulled down protein extracts followed by LC/MS/MS (Fig 1D and E; Supporting Information Fig S2). The data revealed that WT-I2PP2A/SET preferentially binds C_18_-ceramide (about 30% of total C_18_-ceramide) over C_14_–C_16_-ceramides in A549 cells compared to vector-transfected controls (Fig 1D and E, and Supporting Information Fig S2). In addition, WT-I2PP2A/SET also bound to C_20_- (1.9-fold), C_22_- (2.6-fold), C_24_- (2.1-fold) and C_26_-ceramide (2.5-fold) compared to the K209D-I2PP2A/SET in A549 cells (Supporting Information Fig S2). Thus, these data suggest that I2PP2A/SET preferentially binds C_18_-ceramide and, to a lesser extent, C_20_–C_26_-ceramides. As the model suggested, the Y122C-I2PP2A/SET increased binding to C_18_-, C_20_-, C_22_- and C_26_-ceramide around 2.5-, 4.2-, 4.6-, 3.2- and 2.4-fold, respectively, compared to vector-transfected controls (Supporting Information Fig S2). In contrast, the K209D mutation of I2PP2A/SET decreased binding to C_18_–C_26_ ceramides, except for C_14_-ceramide (Supporting Information Fig S2). Thus, these data suggest that the K209D conversion significantly decreases the binding of I2PP2A/SET to C_18_–C_26_-ceramides, while the Y122C mutation enhances ceramide binding, supporting the cation/π-arene gating system of the I2PP2A/SET–ceramide complex.

### Effects of sub-cellular localization of I2PP2A/SET on ceramide binding

To examine whether sub-cellular localization of I2PP2A/SET plays a role in its binding selectivity to endogenously generated ceramides, we generated a mutant of I2PP2A/SET for ER localization/retention (Senkal et al, 2011), where *de novo* ceramide synthesis occurs. We confirmed the ER localization of this mutant (ER-I2PP2A/SET-GFP) *versus* WT-I2PP2A/SET-GFP, K209D-I2PP2A/SET-GFP and Y122C-I2PP2A/SET-GFP using confocal microscopy/immunofluorescence by co-localization of GFP with the ER protein calnexin (Fig 1C). Wt- and mutant-I2PP2A/SET-GFP expression was confirmed by Western blotting (Supporting Information Fig S3). The ER-I2PP2A/SET-GFP was preferentially expressed in the ER, whereas WT-, K209D- and Y122C-I2PP2A/SET-GFP were localized mainly in the nucleus of A549 cells (Fig 1C). We then examined binding of ER-I2PP2A/SET-GFP to endogenous ceramides in A549 cells after pull-down studies using anti-GFP columns followed by lipid extraction and LC/MS/MS (Fig 1D and E, and Supporting Information Fig S2). Interestingly, retention of I2PP2A/SET in the ER shifted its binding preference towards C_16_-ceramide over C_18_-ceramide compared to WT-I2PP2A/SET *in situ* (Fig 1D and E), which was independent of their relative concentrations in the ER *versus* nucleus; C_16_-ceramide was higher than C_18_-ceramide in both cytoplasm and nucleus (see Supporting Information Fig S4A). Moreover, targeting mutation of the ER-I2PP2A/SET-GFP had no inhibitory effect on its binding activity to ceramide compared to WT-I2PP2A *in vitro* (Supporting Information Fig S4B). Thus, these data suggest that ceramide-binding selectivity is mainly regulated by the sub-cellular localization of I2PP2A/SET and availability of ceramides, rather than their fatty acid chain lengths, in ER *versus* nuclear membranes.

### I2PP2A/SET is a novel target for lung cancer

**Figure 2:**
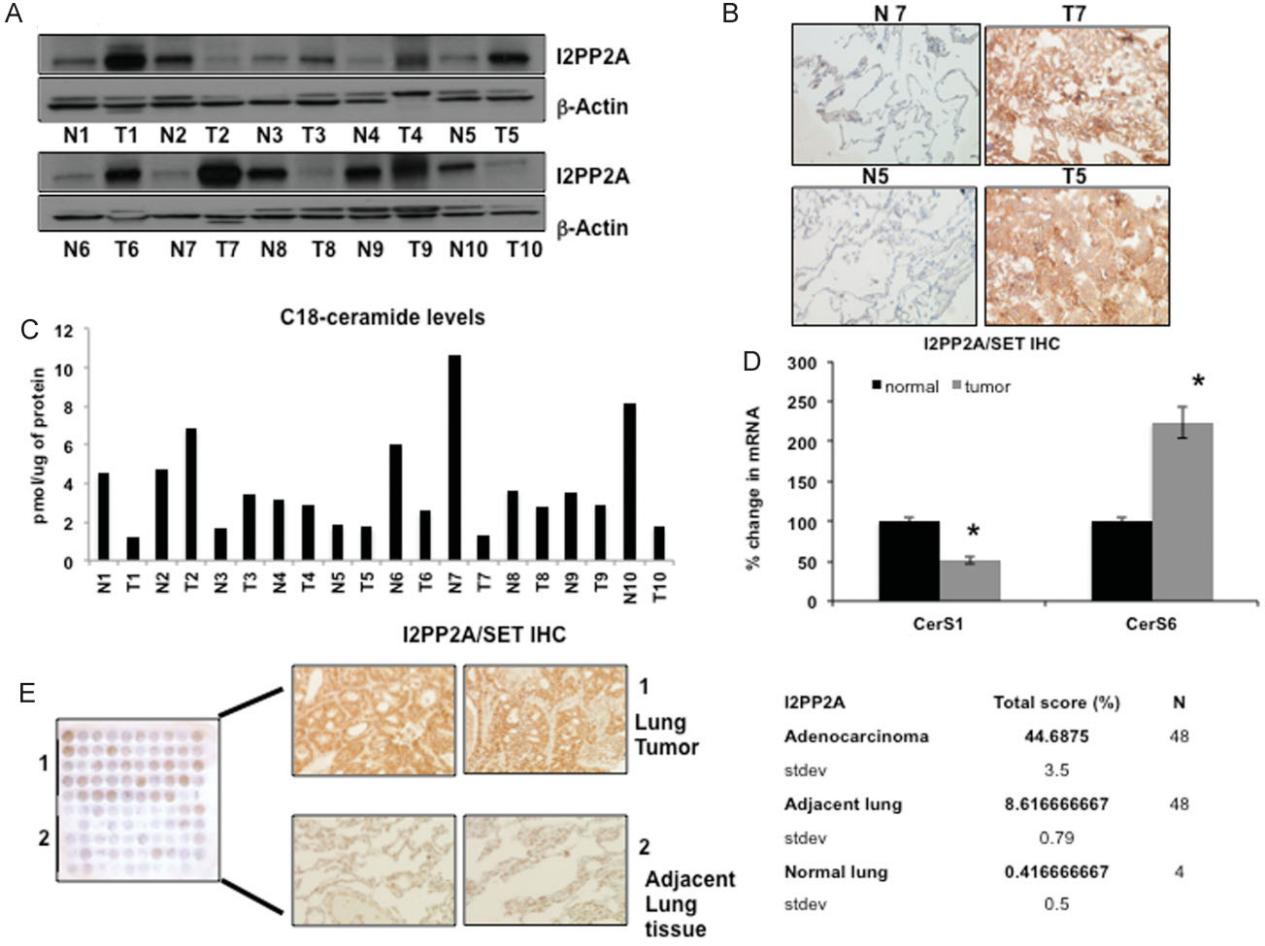
I2PP2A/SET is overexpressed and C_18_-ceramide is decreased in primary lung tumour tissues Ten paired samples of fresh frozen lung adenocarcinoma tumours (T) with adjacent, non-cancerous lung tissues (N) were homogenized and Western blotting was done to evaluate I2PP2A/SET. β-actin was used as a control. Full blots can be seen in Supporting Information Fig S15.I2PP2A/SET was detected in N7, N5 *versus* T7, T5 tissues using IHC (left and right panels, respectively).Ceramides were measured by LC/MS/MS in N1–10 *versus* T1–10 tissues (normalized to µg of protein), and C_18_-ceramide levels in N1–10 compared to T1–10 tissues were reported.CerS1 and CerS6 mRNA were measured by Q-PCR in T1–10 compared to N1–10 tissues (normalized to rRNA).I2PP2A/SET was detected by IHC and scored (percentage of I2PP2A/SET expression was calculated using positive staining scored between 1 and 5 and intensity of staining scored between 1 and 3) in TMAs containing lung tumour (1) and non-cancerous lung tissues (2) (*n* = 48 pairs), and normal lung tissues (*n* = 4). *p* < 0.05 was considered significant (calculated using paired *t*-test).

Binding of I2PP2A/SET to ceramide in lung cancer cells might have clinical and biological significance. To define the clinical relevance of I2PP2A/SET-C_18_-ceramide in cancer pathogenesis, we measured I2PP2A/SET and C_18_-ceramide in tumour *versus* pathologically non-cancerous adjacent lung tissues obtained from 10 patients with NSCLC using Western blotting and immunohistochemistry (IHC; Fig 2A and B). Data showed that I2PP2A/SET is overexpressed in 70% (7/10, *n* = 10) of these tumours (Fig 2A), whereas C_18_-ceramide and CerS1 mRNA levels are decreased (∼50%) in the majority of tumours (8/10, *n* = 10) compared to non-cancerous lung tissues (Fig 2C and D, respectively). Importantly, at least 4 of 10 patients exhibited overexpression of I2PP2A/SET in combination with down-regulation of C_18_-ceramide in their lung tumours, suggesting that tumour suppressive ceramide/PP2A signalling is altered in these tumours (Fig 2C and D). However, C_16_-ceramide was significantly higher (∼1.8-fold, *p* < 0.01, *n* = 10) in tumour compared to non-cancerous lung tissues, and there were no significant changes in the levels of other ceramide species (Supporting Information Fig S5).

The significance of I2PP2A/SET in lung tumours was further explored by IHC using a tumour micro-array (TMA) containing lung adenocarcinoma (*n* = 48), adjacent lung (*n* = 48) and normal lung tissues (*n* = 4). I2PP2A/SET expression was significantly higher in 47 of 48 (*p* < 0.001) lung tumours when compared to adjacent and normal lung tissues (Fig 2E, left and right panels). These data suggest that PP2A might be inhibited in lung tumours, because its biological inhibitor I2PP2A/SET is highly expressed, whereas PP2A activator C_18_-ceramide (Mukhopadhyay et al, 2009) is lower in these tumour tissues compared to controls. In fact, an inactive p-PP2A (Y307) was elevated in the majority of lung tumours compared to paired non-cancerous lung tissues (Supporting Information Fig S6). Overall, these data suggest that targeting I2PP2A/SET might be a novel therapeutic strategy for the treatment of NSCLC to reactivate PP2A tumour suppressor signalling.

### FTY720 directly binds I2PP2A/SET

**Figure 3:**
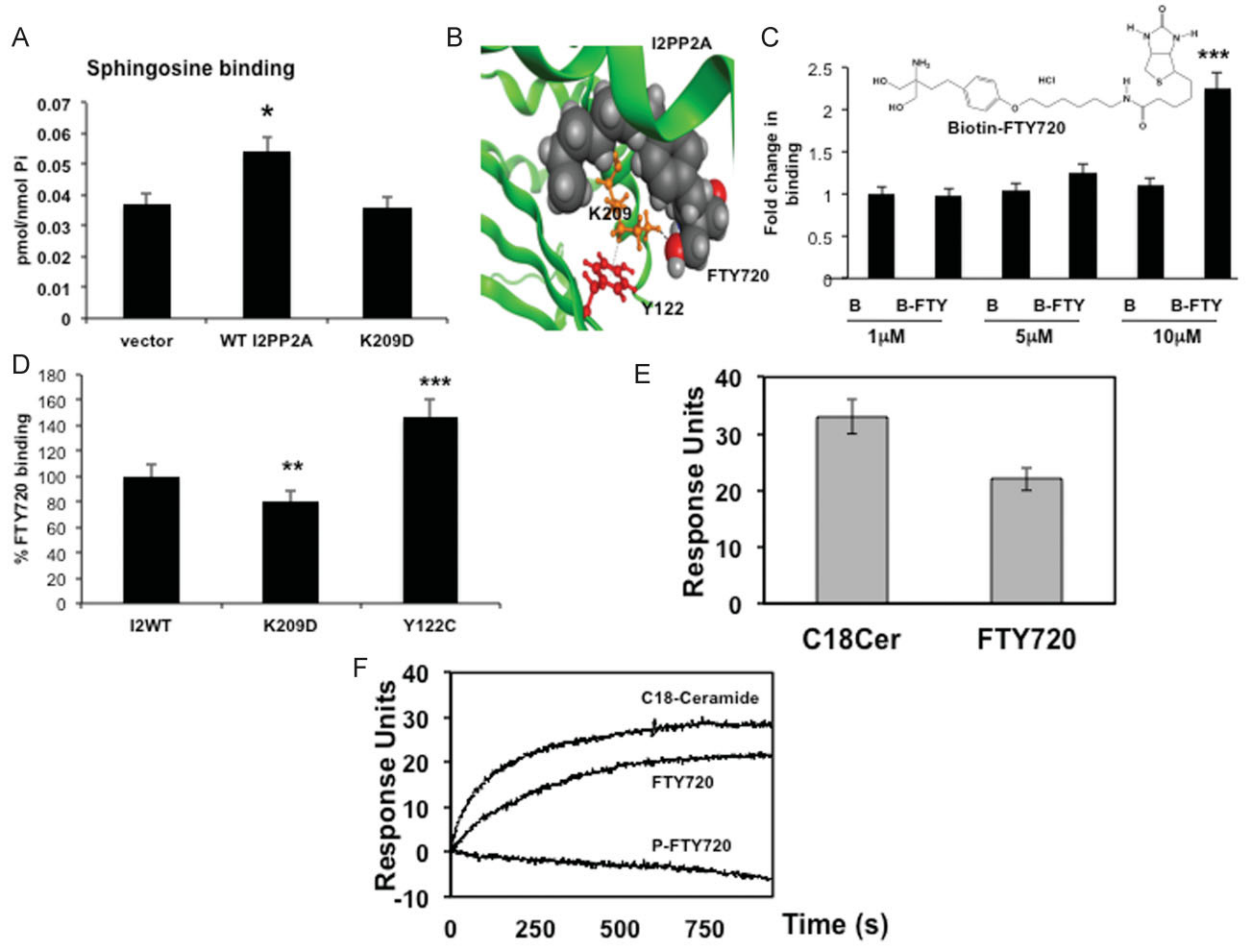
FTY720 binds I2PP2A/SET **A.** Binding of endogenous sphingosine was detected by LC/MS/MS in A549 lung cancer cells after overexpression and pull-down of vector, WT-I2PP2A or K209D-I2PP2A-GFP, using anti-GFP columns.**B.** Molecular docking predicts various residues of I2PP2A/SET that may be involved in FTY720 binding, including K209 and Y122. The 1-OH of FTY720 is expected to interact with the K209, which might form a cationic/π-arene interaction with the Y122 to form a possible gate for FTY720/ceramide binding.**C.** Binding of I2PP2A/SET to B-FTY720 (1, 5 or 10 µM) was detected using avidin pull-down (elution) followed by Western blotting with the anti-I2PP2A/SET antibody. Structure of B-FTY720 is shown. Full blots can be seen in Supporting Information Fig S15.**D.** Binding of B-FTY720 (10 µM) to WT-I2PP2A/SET compared to K209D- or Y122C-I2PP2A-GFP was measured as described in Materials and Methods section.**E,F.** Binding of purified human I2PP2A/SET to C_18_-ceramide and FTY720 (**E**) or P-FTY720 (**F**) were measured using SPR. Saturation values determined for 1 µM I2PP2A/SET injected over the respective active surface. The average response for 1 µM I2PP2A/SET binding to C_18_-ceramide, FTY720 or P-FTY720 over the control POPC:POPE surface, performed in triplicate, were shown. Error bars represent s.d. (*p* < 0.05, *p* < 0.01, *p* < 0.001 were considered significant).

Targeting I2PP2A/SET exogenously with a sphingolipid analogue drug that mimics ceramide and/or sphingosine could potentially bind I2PP2A/SET and reactivate PP2A tumour suppressor signalling (Mukhopadhyay et al, 2009). Since exogenous (Mukhopadhyay et al, 2009) and endogenous sphingosine (Fig 3A) bind wt-I2PP2A/SET, but not K209D-I2PP2A/SET, in A549 cells (Fig 3A), we examined whether sphingosine analogue FTY720, which was shown to inhibit tumour growth (Liu et al, 2010; Neviani et al, 2007; Pchejetski et al, 2010; Wallington-Beddoe et al, 2011), binds/targets I2PP2A/SET. We performed molecular docking studies based on I2PP2A/SET–ceramide modelling (Fig 1A and B), which predicted that one of the primary hydroxyl groups of FTY720 might bind to the K209 residue of I2PP2A/SET (Fig 3B and Supporting Information Fig S7). Biotin-labelled FTY720 (B-FTY720) was incubated with purified I2PP2A/SET, drug-bound proteins were then separated through an avidin column, and B-FTY720-bound I2PP2A/SET was detected by Western blotting using anti-I2PP2A/SET antibody. Remarkably, FTY720 (5–10 µM) also bound directly to purified I2PP2A/SET *in vitro* or endogenously expressed I2PP2A/SET in A549 cells (Fig 3C).

Then, to explore the role of K209 and Y122 residues in the interaction between FTY720 and I2PP2A/SET, the K209D-, Y122C- or WT-I2PP2A/SET-GFP were expressed in A549 cells, and their binding to B-FTY720 was examined as compared to biotin-only-treated controls. The K209D mutation decreased I2PP2A/SET-FTY720 binding (∼20%), whereas the Y122C conversion increased its FTY720 binding (∼45%) compared to WT-I2PP2A/SET, which was considered as 100%, compared to biotin-only-treated controls (Fig 3D). Collectively, these data suggest that FTY720 directly binds I2PP2A/SET both *in vitro* and *in situ*, and that K209/Y122 residues are involved in FTY720-I2PP2A/SET binding, mimicking ceramide as predicted by molecular modelling.

Moreover, binding of FTY720 to purified recombinant human I2PP2A/SET, containing a histidine (His) tag, was also confirmed by surface plasmon resonance (SPR) (Stahelin and Cho, 2001). To quantitatively determine the binding affinity of I2PP2A/SET for liposomes harbouring ceramide, FTY720 or FTY720P, we performed SPR, which directly measures lipid–protein interactions. In these experiments, an active surface was coated with POPC (1-palmitoyl-2-oleoyl-sn-glycero-3-phosphocholine)/POPE (1-palmitoyl-2-oleoyl-snglycero-3-phospho-ethanolamine)-ceramide, FTY720 or P-FTY720 (75:20:5). A control surface was then coated with POPC/POPE (80:20). Recombinant purified I2PP2A/SET or control p47^*phox*^-PX, a phosphoinositide binding domain of p47, which does not bind ceramide, were injected at 1 µM across the active and control surfaces to determine the relative binding for each of the lipids. As shown in Fig 3E, I2PP2A/SET displayed substantial binding to liposomes containing ceramide or FTY720, but binding to P-FTY720 was not detectable. Relative kinetic binding constants ± the standard deviation for 3 replicates are shown in Fig 3F. I2PP2A/SET bound with similar affinity (*K*_d_ = ∼11 nM) to vesicles containing C_18_-ceramide or FTY720 (Table 1), whereas Kd for P-FTY720 was below the detection limit (Table 1). Moreover, a control lipid binding domain p47^*phox*^-PX, which has previously been shown to bind to PI(3,4)P_2_ and phosphatidic acid (Karathanassis et al, 2002), did not display detectable binding on any of the surfaces (Table 1). Overall, these data support the binding preference of I2PP2A/SET for C_18_-ceramide and FTY720 over P-FTY720 *in vitro*.

**Table 1:** Binding of purified human I2PP2A/SET to C_18_-ceramide, FTY720 and P-FTY720 *in vitro*, measured using SPR

Surface	Protein	Protein *K*_d_ (nM)
POPC:POPE	I2PP2A	70 ± 20
C_18_-ceramide^a^	I2PP2A	11 ± 7
FTY720^a^	I2PP2A	11 ± 9
FTY720P	I2PP2A	N.D.^b^
POPC:POPE	p47^*phox*^-PX	N.D.
C_18_-ceramide^a^	p47^*phox*^-PX	N.D.
FTY720^a^	p47^*phox*^-PX	N.D.

*K*_d_ values determined with SPR. All data was collected at 1 µM I2PP2A in 20 mM HEPES, pH 7.4 buffer containing 160 mM KCl.

a Liposomes contained POPC:POPE:*x* at (75:20:5) ratio while control liposomes contained POPC:POPE at a (80:20) ratio.

b N.D. refers to not detectable on the indicated surface.

### Treatment with FTY720 suppresses A549-xenograft-derived tumour growth

**Figure 4:**
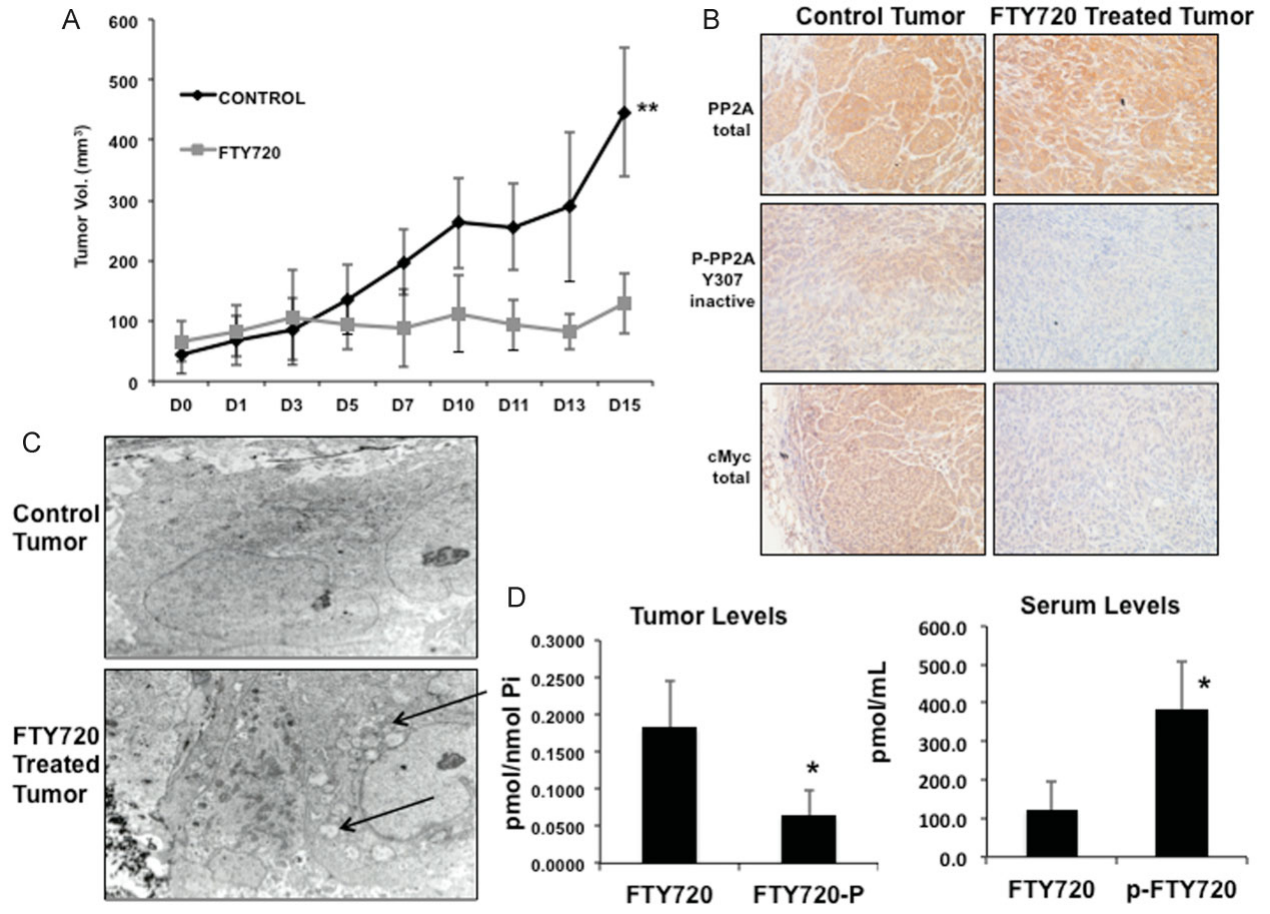
FTY720 inhibits A549-derived xenograft tumour growth in SCID mice Effects of FTY720 (3 mg/kg/day via oral gavage for 15 days) on A549-derived xenograft tumour growth was evaluated in Balb/c SCID mice (*n* = 12). Tumour sizes were measured at every 2–3 days.PP2A (total), P-PP2A (inactive) and cMyc (total) were detected by IHC in tumour tissues obtained from mice treated with FTY720 compared to vehicle-treated controls.Electron microscopy of control and FTY720 treated tumours. Arrows indicate areas of mitochondria damage and cytoplasmic swelling.FTY720 and P-FTY720 were measured by LC/MS/MS in tumour and serum samples obtained from mice treated with FTY720 compared to vehicle treated controls. Error bars show s.d. (*p < 0.05, **p < 0.01).

Because FTY720 directly bound I2PP2A/SET, we explored its ability to inhibit tumour growth via activation of PP2A tumour suppressor signalling. The therapeutic potential of FTY720 against A549-xenografts generated in the flanks of SCID mice was determined after oral administration of FTY720 (3 mg/kg/day for 15 days, *n* = 12) (LaMontagne et al, 2006). FTY720 significantly (*p* < 0.05, *n* = 12) inhibited the growth of A549-xenograft-derived tumours compared to controls (Fig 4A). To examine whether FTY720-mediated tumour suppression was linked to PP2A activation, we examined P-PP2A (inactive) and PP2A (total) in tumours obtained from control *versus* FTY720-treated animals. The data showed that FTY720 treatment decreased inactive P-PP2A (Y307) without affecting total PP2A compared to controls (Fig 4B, upper and middle right/left panels, respectively), suggesting PP2A activation. Because activation of PP2A results in c-Myc degradation (Mukhopadhyay et al, 2009), leading to inhibition of human telomerase reverse transcriptase (hTERT) transcription, we also confirmed FTY720-mediated PP2A activation by measuring c-Myc and hTERT in these tumour tissues. Activation of PP2A by FTY720 was consistent with decreased c-Myc (as detected using IHC or Western blotting) and hTERT mRNA (measured by Q-PCR) in these tumours compared to controls (Fig 4B, lower panel, or Supporting Information Fig S8A and B). In addition, examination of the tumours by transmission electron microscopy (TEM) revealed that ∼40% of tumours (*n* = 20) obtained from mice treated with oral FTY720 had damaged/swollen mitochondria consistent with cellular damage leading to inhibition of tumour growth (Fig 4C).

To confirm that the oral drug treatments resulted in effective FTY720 delivery, we measured FTY720 and P-FTY720 in tumour tissues and serum samples obtained from FTY720-treated mice compared to controls using LC/MS/MS. The data showed that FTY720 accumulated in the A549-xenograft-derived tumours (Fig 4D) reaching 0.2 pmol/nmol Pi, whereas P-FTY720 was much lower in tumours (0.05 pmol/nmol Pi). However, accumulation of P-FTY720 was higher in the serum of animals than FTY720, with ∼400 pmol/ml *versus* ∼100 pmol/ml, respectively (Fig 4D). Thus, these data suggest that FTY720-mediated tumour suppression is associated to PP2A activation *in vivo*, and whereas FTY720 accumulates preferentially in tumour tissues, P-FTY720 mainly accumulates in the serum.

Because FTY720 was shown to alter ceramide metabolism (Berdyshev et al, 2009; Lahiri et al, 2009), we explored whether treatment with FTY720 resulted in changes in ceramide accumulation in A549-xenograft-derived tumours and serum of SCID mice. Interestingly, we found no significant changes in total ceramide levels in the tumour or serum of mice treated with oral doses of FTY720 (Supporting Information Fig S8C).

### Phosphorylation of FTY720 might be dispensible for its I2PP2A/SET binding or tumour suppressor activity

**Figure 5:**
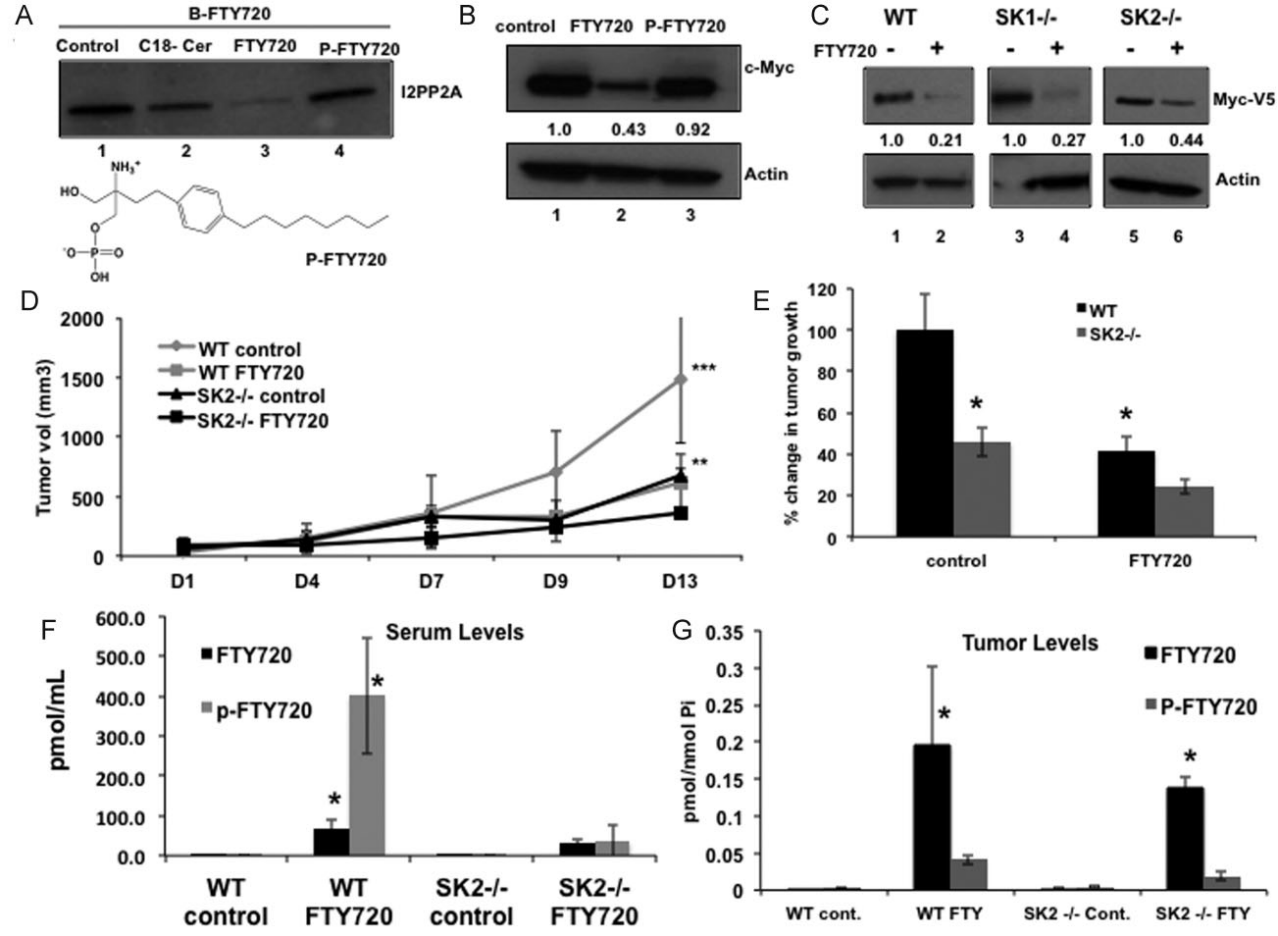
Phosphorylation of FTY720 by SK-2 is dispensable for I2PP2A/SET binding and tumour suppression **A.** Binding of B-FTY720 to purified I2PP2A/SET (elution) was measured by avidin column pull-down followed by Western blotting in the absence/presence of unlabeled C_18_-ceramide, FTY720 or P-FTY720 (10 µM/each), used as competitors. Structure of P-FTY720 is shown.**B.** Effects of FTY720 and P-FTY720 on c-Myc expression in A549 cells were examined by Western blotting. Actin was used as a loading control. Relative c-Myc levels are shown blow the first panel.**C.** Effects of FTY720 on ectopically expressed c-Myc-V5 in WT compared to SK-1^−/−^ and SK-2^−/−^ MEFs were measured by Western blotting. Actin was used as a loading control. Relative c-Myc levels are shown blow the first panel. Full blots can be seen in Supporting Information Fig S15.**D,E.** Roles of FTY720 in tumour suppression was measured in WT (C57B7/6) and SK2^−/−^ mice, bearing Lewis lung adenocarcinoma-derived allografts (**D**), and % change in tumour volume relative to WT and SK-2^−/−^ mice in response to FTY720 compared to controls at Day 13 are shown (**E**).**F,G.** FTY720 and P-FTY720 accumulation in serum (**F**) *versus* tumour tissues (**G**) grown in WT *versus* SK-2^−/−^ mice in the absence/presence of FTY720 were measured using LC/MS/MS (normalized to Pi). Error bars represent s.d. (**p < 0.01, ***p < 0.001).

Phosphorylation of FTY720 is catalysed by nuclear SK-2, and it is required for its immune-suppressor and anti-MS activities (Billich et al, 2003; Paugh et al, 2003). However, whether P-FTY720 generation plays a role in I2PP2A/SET binding or tumour suppression is unknown. Therefore, we first examined whether P-FTY720 is necessary for its I2PP2A/SET binding in A549 cells. To achieve this, purified I2PP2A/SET was incubated with B-FTY720 in the absence/presence of unlabeled C_18_-Pyr-Cer, FTY720 or P-FTY720 as competitors. Incubation with unlabeled C_18_-ceramide or FTY720, but not P-FTY720, competed significantly with B-FTY720-I2PP2A/SET binding (Fig 5A). These data suggest that P-FTY720 is not as efficient as FTY720 to bind I2PP2A/SET, supporting our molecular modelling (Fig 3B) and *in vitro* SPR studies (Fig 3E and F), which suggested that presence of a primary hydroxyl group in the FTY720 structure is important for I2PP2A/SET binding and that I2PP2A/SET has a lower affinity to P-FTY720 compared to C_18_-ceramide and FTY720, respectively.

Because P-FTY720 did not compete with B-FTY720/I2PP2A/SET binding, we explored if the phosphorylation of FTY720 by SK-2 is necessary for its tumour suppressor roles via activation of PP2A. To examine if PP2A is activated by P-FTY720, which leads to c-Myc degradation (Mukhopadhyay et al, 2009; Yeh et al, 2004), A549 cells were treated with FTY720 or P-FTY720, and c-Myc was measured by Western blotting. FTY720, but not P-FTY720, decreased c-Myc levels compared to controls (Fig 5B). Additionally, we investigated whether genetic loss of SK2, in SK2^−/−^ MEFs, modulated FTY720-mediated c-Myc expression, a known endogenous target for PP2A, or cell survival compared to controls. MEFs obtained from WT, SK-1^−/−^, and SK-2^−/−^ mice were ectopically transfected with cMyc-V5 and treated with FTY720, which resulted in reduced c-Myc expression in WT, SK-1^−/−^ and SK-2^−/−^ MEFs by 80, 70, and 55%, respectively, compared to their controls (Fig 5C). Additionally, the IC_50_ values of FTY720 against WT, SK1^−/−^ and SK2^−/−^ MEF's were comparable (∼10 µM; Supporting Information Fig S9). Accordingly, shRNA-mediated stable knockdown of SK-2 (∼75% compared to controls as detected by q-PCR, Supporting Information Fig S9B), had no preventive effect and further increased FTY720-induced cell death compared to Scr-shRNA-transfected A549 cells (Supporting Information Fig S9C). These data suggest that phosphorylation of FTY720 by SK-2 might be dispensible for the regulation of the PP2A/c-Myc axis and growth inhibitory roles of FTY720 in A549 cells.

To examine if P-FTY720 is involved in targeting I2PP2A/SET leading to tumour growth inhibition *in vivo*, we implanted Lewis lung adenocarcinoma cell (LLC)-derived allografts in the flanks of SK-2^−/−^ compared to WT mice and treated with oral doses of 10 mg/kg FTY720. The genetic loss of SK-2 significantly (*p* < 0.05) reduced the growth of LLC-derived allografts in the flanks of SK-2^−/−^ compared to WT mice and, more importantly, treatment with FTY720 (10 mg/kg/day, via oral delivery) was efficacious against LLC-allografts grown in WT and SK-2^−/−^ mice, inhibiting tumour growth by ∼60 and 47%, respectively, compared to their controls (Fig 5D and E). Interestingly, the loss of SK-2 almost completely abrogated the accumulation of P-FTY720 in the serum of SK-2^−/−^ mice (Fig 5F), whereas FTY720 accumulation was comparable in the tumours grown in WT and SK-2^−/−^ mice (Fig 5G). P-FTY720 was lower than FTY720 in the tumours grown in WT and SK-2^−/−^ animals (Fig 5G). Thus, these data support that SK-2 is important for the generation and accumulation of P-FTY720 in the serum and that SK-2-dependent P-FTY720 may not be required for mechanisms of tumour suppression that are dependent on I2PP2A/SET-targeting. It should be noted that loss of SK-2 had slight protective effects against c-Myc degradation and tumour suppression (∼20–30%) in SK-2^−/−^ MEFs or SK-2^−/−^ k/o mice, respectively, compared to controls (Fig 5C–E). These data indicate that there might be a role for P-FTY720 in the regulation of tumour growth, possibly via I2PP2A/SET/PP2A-independent mechanisms. Nevertheless, our data indicate that immune suppressor and lung tumour suppressor functions of P-FTY720 and FTY720 are distinct, consistent with their preferential accumulation in the serum *versus* lung tumour tissues, respectively.

### Targeting I2PP2A/SET by FTY720 leads to growth inhibition via PP2A activation

**Figure 6:**
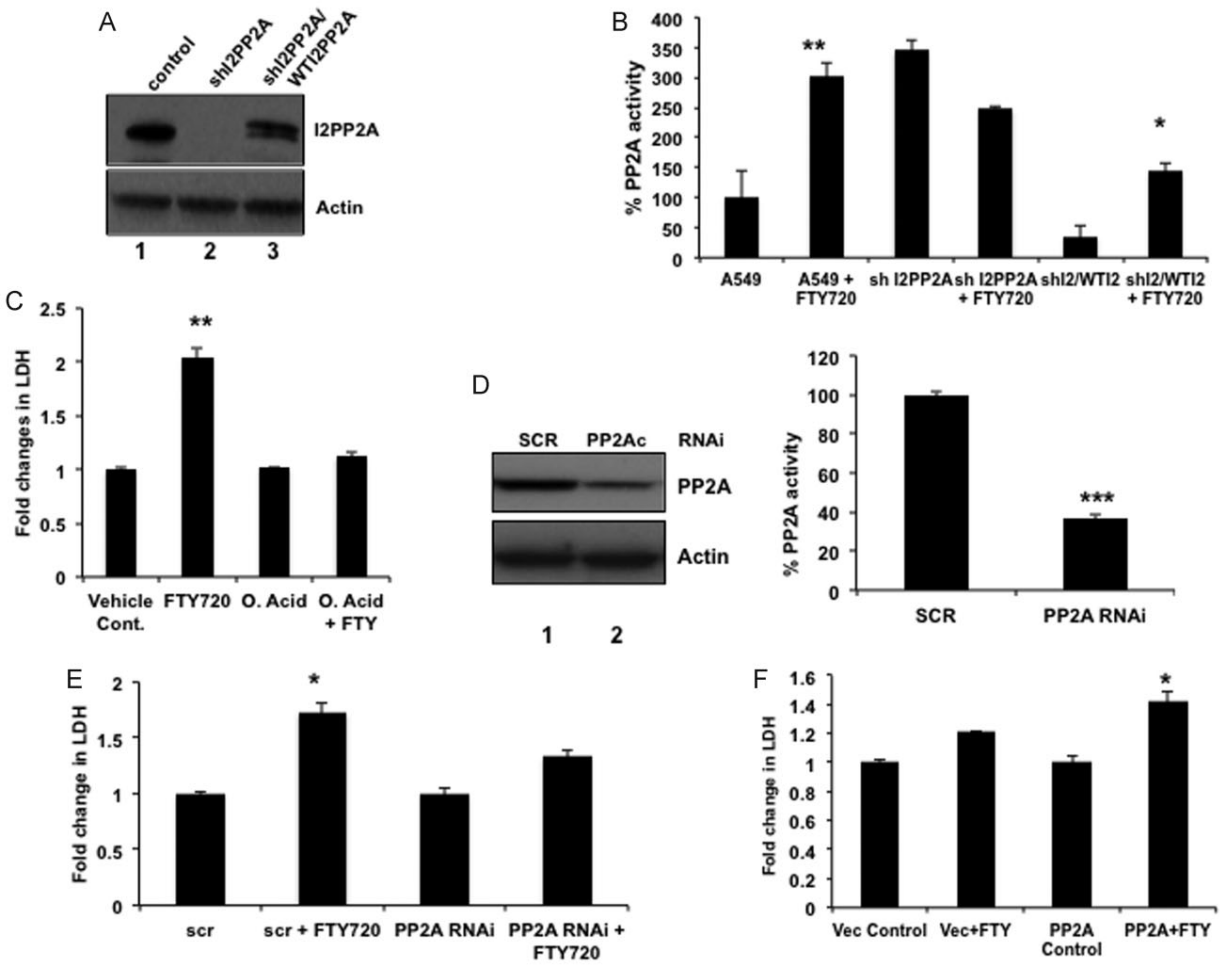
FTY720-I2PP2A/SET binding activates PP2A **A.** Knockdown of I2PP2A/SET using shRNAs and reconstitution of WT-I2PP2A compared to controls in A549 cells were confirmed by Western blotting. Actin was used a loading control.**B.** PP2A activity assay was performed using extracts obtained from A549, A549/shI2PP2A and A549/shI2PP2A/WT-I2PP2A/SET grown in the absence/presence of 10 µM FTY720.**C.** Effects of FTY720 (20 µM) on cell death was measured by detection of LDH secretion in the absence/presence of OA (10 nM).**D,E.** Roles of si-RNA-mediated PP2A knockdown (confirmed by Western blotting, shown in left panel) in the modulation of PP2A activity (**D**) or FTY720-mediated cell death (**E**) were measured in A549 cells compared to Scr-siRNA controls (right panel).**F.** Effects of PP2A-HA expression on cell death in the absence/presence of FTY720 was measured compared to controls. Error bars represent s.d. (*p < 0.05, **p < 0.01). Full blots can be seen in Supporting Information Fig S16.

To examine whether binding/targeting of I2PP2A/SET by FTY720 inhibits lung tumour growth via PP2A activation, we measured PP2A activity in response to shRNA-mediated I2PP2A knockdown (∼90%) in A549 cells (Fig 6A). Then, effects of WT-I2PP2A/SET reconstitution (∼60%) in A549/sh-I2PP2A/SET cells on PP2A activity in response to FTY720 was also measured compared to controls (Fig 6A). Interestingly, whereas FTY720 enhanced PP2A activity compared to vehicle-treated controls, knockdown of I2PP2A/SET also significantly enhanced PP2A activity, but prevented its further activation by FTY720 (Fig 6B). In contrast, when WT-I2PP2A/SET was restored in A549/sh-I2PP2A/SET cells, the PP2A activity was increased in response to FTY720 (Fig 6B). Thus, these data suggest that FTY720 activates PP2A via targeting I2PP2A/SET.

To examine whether I2PP2A/SET-FTY720 binding inhibits growth in lung cancer cells via PP2A activation, we pre-treated cells with okadaic acid (OA, 10 nm), a PP2A inhibitor, and detected that inhibition of PP2A prevented FTY720-mediated growth suppression (Fig 6C). Moreover, siRNA-mediated knockdown of PP2A [∼70% compared to scrambled (SCR) siRNA-transfected cells; Fig 6D, left panel] inhibited PP2A activity around 70% (Fig 6D, right panel) and attenuated FTY720-mediated cell death measured by LDH release to the media (Fig. 6E). In reciprocal experiments, overexpression of PP2A-HA, as confirmed by Western blotting (Supporting Information Fig S10A), increased FTY720-mediated cell death ∼40% compared to controls (Fig 6F). In these studies, it was noted that overexpression of PP2Ac itself had no significant effect on cell viability compared to vector-transfected controls (Fig 6F). PP2A forms over 150 interprotein and 570 intraprotein complexes (Herzog et al, 2012). Therefore, it is difficult to interpret the effects of overexpression of the catalytic subunit of PP2A itself on cell viability, because it is unknown, which specific PP2A interprotein or intraprotein complexes are involved in its tumour suppressor function after being released from I2PP2A/SET in response to FTY720. These data suggest then that FTY720 might not only relieve PP2Ac from the inhibitor, but it might also induce its anti-tumour activity via mediating specific interprotein or intraprotein complexes of PP2A (Zhang et al, 2009). This, however, needs to be further evaluated.

### Targeting of I2PP2A/SET is important for tumour suppressor effects of FTY720

**Figure 7:**
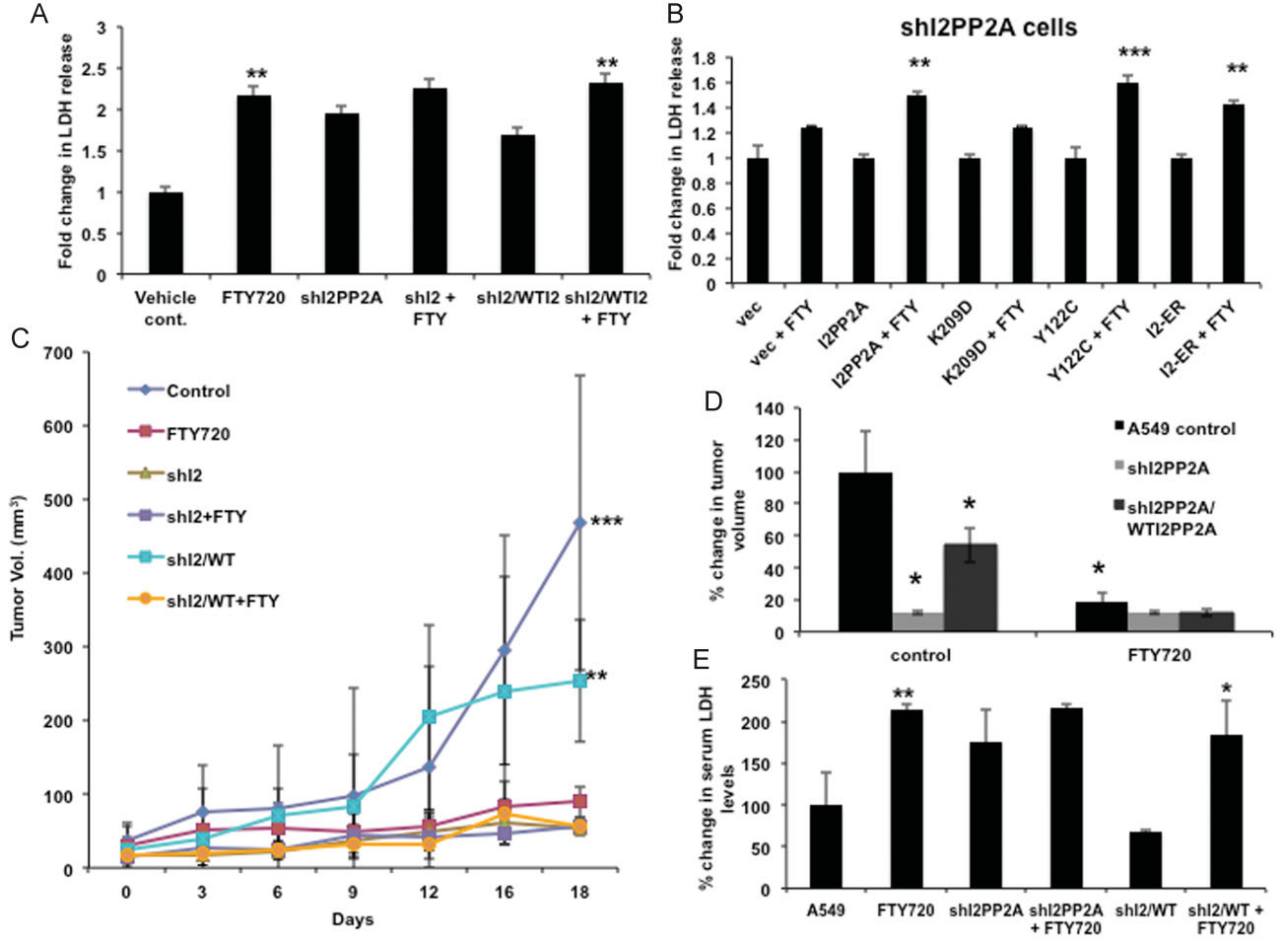
Targeting I2PP2A/SET by FTY720 inhibits tumour growth **A.** Effects of FTY720 on cell death were measured in A549/sh-control, A549/sh-I2PP2A/SET and A549/sh-I2PP2A/SET/WT-I2PP2A/SET cells, in the absence/presence of 20 µM FTY720 or vehicle control for 24 h.**B.** Cell death in response to FTY720 in A549/sh-I2PP2A/SET cells in the absence/presence of WT-, K209D-, Y122C- or ER-I2PP2A/SET expression was measured by detection of LDH levels compared to controls.**C,D.** Effects of FTY720 on tumour growth were measured in Balb/c SCID mice bearing A549/sh-control, A549/sh-I2PP2A/SET and A549/sh-I2PP2A/SET/WT-I2PP2A/SET xenografts compared to controls for 18 days. The mice were treated daily with 3 mg/kg FTY720 by oral gavage (**C**). Percent change in tumour volume relative to untreated A549/sh-control mice at Day 18 are shown (**D**).**E.** Levels of LDH release to the serum in response to FTY720 treatment compared to untreated controls in SCID mice bearing A549/sh-control, A549/sh-I2PP2A/SET and A549/sh-I2PP2A/SET/WT-I2PP2A/SET xenografts were measured by Analytics Inc. Error bars represent s.d. (*p < 0.05, **p < 0.01, ***p < 0.001).

To validate I2PP2A/SET as a target of FTY720 for its tumour suppressor roles, we determined the effects of FTY720 on cell death and/or tumour suppression in response to shRNA-mediated knockdown of I2PP2A and its reconstitution in A549/sh-I2PP2A/SET *versus* A549/sh-I2PP2A/SET-WT-I2PP2A/SET cells *in situ* and *in vivo*. Treatment with FTY720 or knockdown of I2PP2A/SET alone significantly induced cell death compared to their respective controls (Fig 7A). Importantly, knockdown of I2PP2A/SET prevented FTY720-induced cell death (Fig 7A). Reconstitution of WT-I2PP2A/SET expression in A549/sh-I2PP2A/SET cells restored FTY720-mediated cell death (Fig 7A). These data were consistent with the effects of I2PP2A/SET knockdown and expression of WT-I2PP2A/SET on PP2A activation in the absence/presence of FTY720 in these cells (Fig 6B). Expression of Y122C-I2PP2A/SET-GFP and ER-I2PP2A/SET-GFP restored FTY720-mediated cell death when endogenous I2PP2A/SET was down-regulated compared to controls in A549/sh-I2PP2A/SET cells (Fig 7B). However, expression of K209D-I2PP2A/SET had no significant effect on cell death in response to FTY720 in A549/sh-I2PP2A/SET cells (Fig 7B). I2PP2A/SET-GFP expression in A549/sh-I2PP2A/SET cells was confirmed by Western blotting (Supporting Information Fig S10B).

To confirm that tumour suppressor roles of FTY720 requires I2PP2A/SET targeting *in vivo*, we generated xenografts in SCID mice using A549/shcontrol, A549/sh-I2PP2A/SET and A549/sh-I2PP2A/SET cells that express wt-I2PP2A/SET and assessed their growth with/without oral FTY720 treatment (3 mg/kg/day) compared to vehicle-treated controls. FTY720 inhibited the growth of A549/control-derived xenografts ∼80% compared to vehicle-treated controls (Fig 7C). Knockdown of I2PP2A/SET alone inhibited tumour growth ∼85%, however, FTY720 had no additional effects on tumour suppression in response to I2PP2A/SET knockdown. Interestingly, reconstitution of WT-I2PP2A/SET expression in A549/sh-I2PP2A/SET cells increased growth of tumours ∼50% compared to controls (Fig 7C). Expression of WT-I2PP2A/SET in A549/sh-I2PP2A/SET cells restored tumour suppressor effects of FTY720 (Fig 7C and D). Overall, these data suggest that FTY720-I2PP2A/SET binding and targeting plays a critical role, at least in part, in FTY720-mediated cell death and tumour suppression.

Because our data suggest that FTY720 mediates cell death, which can be measured by elevation of LDH released in the growth media, we next examined whether elevation of LDH in A549-xenograft-derived tumours treated with FTY720 can also be detected in the serum of mice. Total LDH released in the serum of animals containing tumours derived from A549/sh-control, A549/sh-I2PP2A/SET and A549/sh-I2PP2A/SET/WT-I2PP2A/SET cells in the absence/presence of FTY720 was measured. The data show that FTY720 or shRNA-mediated knockdown of I2PP2A/SET, both of which decreased tumour growth, increased serum LDH in mice (Fig 7E). However, FTY720 treatment had no additional effect on serum LDH in tumours expressing shRNA against I2PP2A/SET (Fig 7E), consistent with the lack of enhanced tumour growth inhibition by FTY720. Remarkably, reconstitution of WT-I2PP2A/SET in A549/sh-I2PP2A/SET enhanced serum LDH in response to FTY720, consistent with its role in restoring FTY720-mediated tumour suppression *in vivo*. Thus, these data suggest that tumour suppression in response to targeting I2PP2A/SET by FTY720 can be detected by the elevation of serum LDH *in vivo*.

### FTY720 induces lung cancer cell death via RIPK1-mediated necroptosis

**Figure 8:**
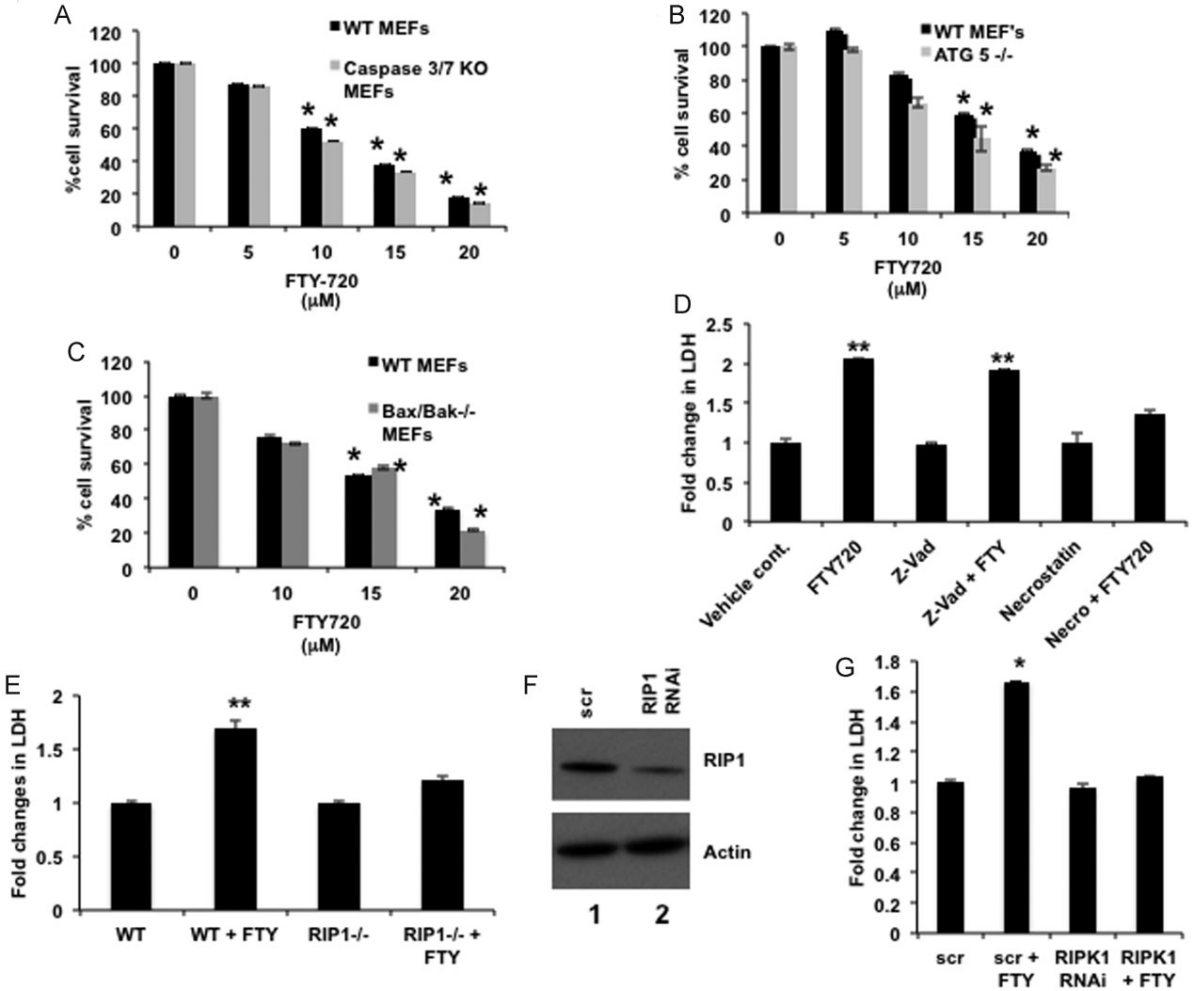
FTY720 mediates cell death via PP2A/RIPK1-dependent necroptosis **A–C.** Effects of FTY720 at various concentrations on growth of MEFs obtained from caspase 3/7^−/−^ (**A**), Bax/Bak^−/−^ (**B**) or ATG5^−/−^ (**C**) mice were measured using MTT assays compared to vehicle-treated controls.**D.** Effects of Z-VAD or necrostatin on FTY720-mediated cell death were measured in A549 cells compared to vehicle treated controls. Cell death was measured by LDH release in the media.**E.** Effects of FTY720 (15 µM) on cell death were measured in WT and RIPK1^−/−^ MEF's compared to vehicle-treated controls.**F,G.** Roles of siRNA-mediated knockdown of RIPK1 compared to Scr-siRNAs-transfected controls, confirmed by Western blotting (**F**), in FTY720-mediated cell death were measured by detection of LDH release (**G**). Error bars represent s.d. (*p < 0.05, **p < 0.01). Full blots can be seen in Supporting Information Fig S16.

To delineate the downstream mechanism by which targeting I2PP2A/SET by FTY720 results in cell death [as confirmed by increased Annexin V/7-aminoactinomycin D (7-AAD) positive A549 populations compared to controls using flow cytometry; Supporting Information Fig S11A], we examined the effects of FTY720 on apoptotis- or autophagy-dependent cell death using MEFs obtained from caspase3/7^−/−^ DKO, Bax/Bak^−/−^ DKO or ATG5^−/−^ KO mice. The data showed that genetic loss of caspases3/7, Bax/Bak, or ATG5 had no protective effect on FTY720-induced cell death in these immortalized MEFs (Fig 8A–C), which express endogenous I2PP2A/SET (Supporting Information Fig S11B). Consistent with these data, treatment with a pan-caspase inhibitor Z-VAD had no effect on A549 cell death in response to FTY720 (Fig 8D). Accordingly, ATG5^−/−^ MEFs were more sensitive to taxol-induced apoptosis (80 nM and 24 h) as measured by increased caspase 3 activity compared to WT MEFs and used as an additional control to confirm their expected response to chemotherapy-induced apoptosis (Supporting Information Fig S11C). Overall, these data suggest that FTY720 induces cell death independently of caspase/Bax/Bak-mediated apoptosis or ATG5-mediated autophagy.

Because FTY720-mediated cell death was detected by elevated LDH, a measure of primary necrosis and plasma membrane breakdown, which occurs early during regulated necrosis or late during apoptosis (secondary necrosis), we determined the effects of necrostatin, an inhibitor of RIPK-1-mediated programmed necrosis (Degterev et al, 2008) (necroptosis), in this process. Pre-treatment with necrostatin significantly prevented (*p* < 0.05) cell death in response to FTY720 (Fig 8D). Accordingly, the genetic loss of RIPK1 (Ramnarain et al, 2008) almost completely abrogated cell death in response to FTY720 in RIPK1^−/−^ MEFs compared to RIPK1^+/+^ MEFs (Fig 8E). These data were also consistent when endogenous RIPK1 was knocked down (about 80% compared to Scr-siRNA-transfected controls, measured by Western blotting; Fig 8F), FTY720-mediated cell death was blunted in A549 cells compared to controls (Fig 8G). Thus, these data suggest that FTY720-mediated cell death is regulated via induction of RIPK1-dependent necroptosis.

**Figure 9:**
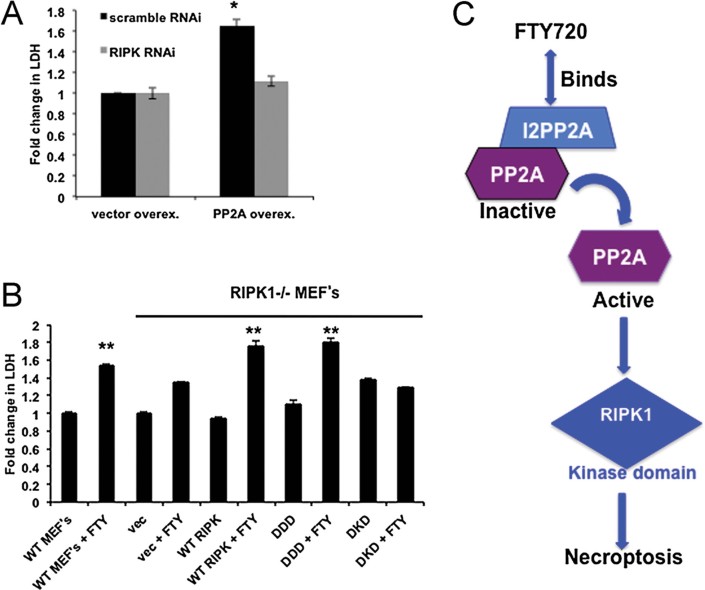
FTY720-mediated lung tumour suppression involves the kinase domain of RIPK1 Roles of siRNA-mediated knockdown of RIPK1 on cell death in the absence/presence of ectopic PP2Ac-HA expression were measured by detection of LDH release compared to controls.Roles of ectopic expression of WT-, DDD-, or DKD-RIPK1 in the regulation of cell death were measured by detection of LDH release in RIPK1^−/−^ MEFs. Error bars represent s.d. (*p < 0.05, **p < 0.01).Our novel data suggest that FTY720 directly binds and targets I2PP2A/SET oncoprotein, mimicking sphingosine/ceramide, which results in PP2A activation, subsequently leading to RIPK1-dependent necroptosis, and lung tumour suppression.

Because I2PP2A/SET-FTY720 binding activated PP2A, we explored the role of PP2A on RIPK1-induced necroptosis. Ectopic PP2Ac (catalytic domain) expression alone or in combination with FTY720 induced necroptosis, however, siRNA-mediated knockdown of RIPK1 prevented necroptosis in response to PP2Ac (Fig 9A). Induction of PP2A-dependent necroptosis by FTY720 was also detected in H157 (K-Ras mutant) and H827 (EGFR mutant) human lung cancer cells, in which inhibition of RIPK1 or PP2A using necrostastin or OA and knockdown of RIPK1 or PP2Ac prevented FTY720-mediated cell death compared to controls (Supporting Information Fig S12A–C). Interestingly, ectopic PP2Ac had no effect on FTY720-mediated cell death in RIPK1^−/−^ MEFs compared to vector-transfected controls (Supporting Information Fig S13A). Interestingly, siRNA-mediated knockdown of RIPK3, a known inducer of necroptosis via MKLN activation (Sun et al, 2012), or Drp1, a downstream target of PGAM5, which is a recently identified necroptosis inducer (Wang et al, 2012), did not prevent FTY720-mediated A549 cell death (Supporting Information Fig S13B–D). In fact, knockdown of RIPK3 increased FTY720-mediated LDH release around twofold compared to controls (Supporting Information Fig S13D). Thus, these data indicate that PP2A activation plays an upstream role in selective RIPK1-dependent necroptosis by FTY720 in these cells.

RIPK1 is composed of three domains: kinase, intermediate and death domains. To explore which RIPK1 domains play a role in FTY720-induced necroptosis, death-domain-deletion mutant (DDD), kinase domain deletion mutant (DKD) and WT-RIPK1 were expressed in RIPK1^−/−^ MEFs and their effects on FTY720-mediated necrosis were determined. Interestingly, WT-RIPK1 and DDD-RIPK1 restored FTY720-mediated necroptosis in RIPK1^−/−^ MEFs, whereas DKD-RIPK1 expression had no effect on this process (Fig 9B). WT-, DDD- and DKD-RIPK1 expression was confirmed with Western blotting (Supporting Information Fig S14). Thus, these data suggest that FTY720-mediated necroptosis requires functional kinase domain of RIPK1 and that the death domain of RIPK1 is dispensible for this process. Overall, these data suggest that direct targeting of I2PP2A/SET by FTY720 induces PP2A/RIPK1-dependent necroptosis through its kinase domain, leading to cell death and subsequent tumour suppression.

## DISCUSSION

In this study, we first characterized the details of interaction between endogenous ceramide and I2PP2A/SET, which is regulated by both structural characteristics and sub-cellular localization of I2PP2A/SET and/or ceramides with different fatty acid chain lengths, whose accumulation was altered in lung tumours. We then utilized these data to develop a novel strategy for lung cancer treatment via directly targeting I2PP2A/SET, which is highly expressed in these tumour tissues, by FTY720. Our novel data suggested that FTY720, but not P-FTY720, directly binds and targets I2PP2A/SET, mimicking ceramide/sphingosine, which activates tumour suppressor PP2A, subsequently suppressing lung tumour growth selectively via inducing RIPK1-mediated necroptosis (Fig 9C).

Molecular modelling/simulation studies suggested that the hydrophobic putative sphingolipid/ceramide-binding pocket of I2PP2A/SET is localized within the two anti-parallel beta sheet stabilized loops and an alpha helix, a common structural feature observed in CERT-ceramide binding (Kudo et al, 2008), which is possibly gated by interaction between K209 and Y122 residues. It should also be noted that although there are some structural similarities between binding sites of CERT and I2PP2A/SET to ceramide, there are also major differences. For example, ceramide binds to CERT with the two aliphatic chains packed against each other. The I2PP2A/SET model has the alkyl side chains splayed out in opposite directions. Moreover, the polar interactions of ceramide and CERT are nicely buried in the pocket, whereas in I2PP2A/SET ceramide interactions with K209 are on the surface and would have to compete with solvent, which might be less favourable. Therefore, details of precise binding site of I2PP2A/SET to ceramide need to be explored in future structural studies.

Our data also revealed that whereas the structural properties of I2PP2A/SET is important to determine its ceramide/sphingolipid binding, fatty acid chain length selectivity toward C_18_- *versus* C_16_-ceramide binding is mainly dependent upon nuclear *versus* ER localization of I2PP2A/SET. These data indicate that availability of C_18_- *versus* C_16_-ceramides for I2PP2A/SET binding in the nucleus *versus* ER membranes, respectively, is distinct in A549 cells. Overall, targeting I2PP2A/SET to different sub-cellular compartments might provide a unique molecular tool to probe the accumulation/availability of distinct ceramides in various cells.

Ceramide is known to play important functional roles in the nucleus, such as the regulation of SP3-HDAC1 suppressor function for the repression of hTERT (Wooten-Blanks et al, 2007). Interestingly, I2PP2A/SET is also involved in gene transcription and histone acetylation (Seo et al, 2001). However, how ceramides accumulate in nuclear membranes and whether ceramide–I2PP2A/SET interaction regulates only nuclear functions of PP2A and/or histone acetylation/deacetylation to suppress growth/proliferation remain unknown.

We also show here that FTY720 mimiks ceramide/sphingosine for I2PP2A/SET binding. Phosphorylation of FTY720 by SK-2 (Billich et al, 2003) is crucial for its anti-MS function via immune-suppression. However, whether anti-cancer functions of FTY720 (Pyne et al, 2011) are regulated via its phosphorylation is unclear. For example, in Jurkat cells, P-FTY720 analogue induces apoptosis (Don et al, 2007), whereas anti-cancer functions of FTY720, but not P-FTY720, have been shown in various different cancer models (Neviani et al, 2007; Roberts et al, 2010). Accordingly, our data elucidates that in lung cancer cells, phosphorylation of FTY720 by SK-2 might be dispensable for I2PP2A/SET targeting, which leads to necroptosis and lung tumour suppression. In contrast, loss of SK-2 prevented tumour suppression by FTY720 slightly (∼20–30%) in SK-2^−/−^ compared to wt MEFs, indicating that P-FTY720 might play some roles in growth inhibition via I2PP2A/SET-PP2A-independent mechanisms in these cells. Alternatively, loss of SK-2 might affect the uptake/metabolism of FTY720, which would account for this slight decrease in tumour growth inhibition.

Mechanisms by which FTY720 mediates cell death are unclear. FTY720 was shown to induce apoptosis (Don et al, 2007) or caspase-independent cell death in acute lymphoblastic leukaemia cells (Wallington-Beddoe et al, 2011). Our novel data suggest that targeting I2PP2A/SET by FTY720 suppresses lung tumour growth via PP2A-dependent RIPK1 activation, leading to necroptosis (Bonapace et al, 2010; Cho et al, 2009; Christofferson & Yuan, 2010; Weinlich et al, 2011). Moreover, RIPK3/MKLN1 and PGAM5/Drp1 appear to be dispensable in this process; however, their roles in FTY720-mediated necroptosis and lung tumour suppression need to be further investigated. Importantly, our data revealed that the kinase domain, but not the death domain, of RIPK1 was required for FTY720-mediated necrosis, consistent with the role of the kinase domain of RIPK1 in the regulation of necroptosis (Cho et al, 2009; Hitomi et al, 2008; Holler et al, 2000), whereas the death domain of RIPK1 is associated with receptor-mediated cell death (Biton & Ashkenazi, 2011). However, whether the activation of PP2A directly regulates the kinase domain of RIPK1 for the induction of necroptosis in response to FTY720 remains unknown. Induction of necroptosis in tumours in response to FTY720 treatment was also monitored by the elevation of total LDH in serum, providing a unique serum marker to monitor tumour suppression *in vivo*. Moreover, it has been shown that FTY720 affects ceramide metabolism (Berdyshev et al, 2009; Lahiri et al, 2009). Consistent with recent studies (Berdyshev et al, 2009; Neviani et al, 2007), our data showed that FTY720 had no significant effect on total ceramide accumulation in tumours or serum of animals, indicating that FTY720-mediated tumour suppression might be independent of altered ceramide accumulation/metabolism.

In summary, by dissecting ceramide/sphingosine-I2PP2A/SET signalling, we identified that I2PP2A/SET is a novel drug target in human lung tumours and, more importantly, that it can be specifically targeted by the sphingosine analogue FTY720 leading to PP2A activation and RIPK1-dependent tumour necroptosis. Collectively, these data have important clinical implications for the development of novel and mechanism-based targeted therapeutics for the treatment of lung cancers.

## MATERIALS AND METHODS

See Supporting Information for additional data (including full blots), and description of reagents, cell lines, culture conditions, Western blotting, IHC, ceramide/FTY720 measurements using LC/MS/MS, immunofluorescence/confocal microscopy, cloning and purification of recombinant human I2PP2A/SET, cell toxicity/survival assays, TEM, animal studies and statistical analysis.

### Molecular modelling of I2PP2A/SET with ceramide or FTY720

Modelling and simulations were performed using MOE. Structural pbd files used were 2E50 for I2PP2A/SET (PMID 17360516) and 2E3P for CERT (PMID 18184806). Before analysis or simulations, proteins were protonated at pH 7.4 and structures energy minimized with heavy atoms constrained. Initial simulations focused on using ceramide as a probe for potential interaction sites, docked to the WT-I2PP2A/SET, or K209D-I2PP2A/SET homodimers using the entire surface as a target. Initial placement calculated 500 poses using triangle matching with London dG scoring, the top 250 poses were then refined using forcefield and ASE scoring. ProCAM analysis was performed using the SAPS ProCAM webserver (http://ci.vbi.vt.edu/cammer/saps.html; PMID: 14696387).

### SPR binding assays

All SPR measurements were performed at 25°C as described (Stahelin and Cho, 2001). Briefly, after washing the sensor chip surface, 80 µl of vesicles containing either POPC:POPE (80:20) or POPC:POPE:*x* (75:20:5, where *x* = C_18_-ceramide, FTY720 or P-FTY720) were injected at 5 µl/min to give a response of 6200 resonance units (RU) where the POPC:POPE (80:20) surface serves as the control. Each lipid layer was stabilized by injecting 10 µl of 50 mM NaOH three times at 50 µl/min. SPR measurements were done at the flow rate of 5 µl/min where 80 µl of protein (I2PP2A/SET or p47^*phox*^-PX control) in 20 mM HEPES, pH 7.4, containing 0.16 M KCl was injected to monitor the association and dissociation phases of binding. To repeat binding measurements to the same lipid vesicles, the lipid surface was regenerated using five 10 µl injections of 50 mM NaOH at 50 µl/min. After sensorgrams were obtained in triplicate for each protein for each lipid condition, the *k*_a_ and *k*_d_ were determined from individual curves by separately fitting each curve with the BIAevaluation software (Biacore). From the *k*_a_ and *k*_d_ values, the equilibrium dissociation constant (*K*_d_) was determined for each lipid surface using the equation *K*_d_ = *k*_d_/*k*_a_.

### Detection of I2PP2A/SET–ceramide or -FTY720 binding

To measure the association between I2PP2A/SET or mutant I2PP2A/SET and endogenous ceramide (Saddoughi et al, 2011), I2PP2A/SET-wt or various mutant-I2PP2A/SET containing GFP tag were overexpressed in A549 cells. Next, over-expressed wt- and mutant-I2PP2A/SET were pulled down from cell extracts (equal amount of total protein was used for each sample) by immunoprecipitation using the µMACS™ GFP Tag protein isolation kit. The equal volume for each sample (after immunoprecipitation) was sent to Lipidomics Core Facility at MUSC (Bielawski et al, 2010) for endogenous ceramide measurement (normalized to inorganic phosphate, Pi) (Mukhopadhyay et al, 2009). For FTY720 binding, I2PP2A/SET was purified from pTrc-I2PP2A/SET-HisA vector, and 1–2 µg were incubated with biotin or biotin-FTY720. The biotin or biotin-FTY720 was immunopreciptated with µMACS Streptavidin Kit (130-074-101), and the elution was run on SDS–PAGE and blotted for I2PP2A/SET. To evaluate FTY720 binding to the I2PP2A/SET mutants, I2PP2A/SET-WT-GFP and mutants were overexpressed in A549 lung cancer cells. The cells were lysed and incubated with biotin, or biotin-FTY720 and were immunoprecipitated with µMACS Streptavidin Kit in the absence/presence of FTY720, C_18_-ceramide, or P-FTY720. The eluted samples were run on SDS–PAGE and blotted for GFP.

**The paper explained**

PROBLEM:

PP2A is a master tumour suppressor enzyme involved in the regulation of key oncoproteins, such as c-Myc and Bcr-Abl in various cancer types including lung cancers and CML, respectively. In addition to inactivation mutations of PP2A, there are biological inhibitors, such as inhibitor 2 of PP2A (I2PP2A/SET oncoprotein), which directly binds and inhibits PP2A function. However, mechanisms involved in the regulation of PP2A-I2PP2A/SET interaction for controlling PP2A-dependent tumour suppression in cancer cells have been enigmatic.

RESULTS:

In this study, molecular modelling and simulations coupled with site-directed mutagenesis indicated that the endogenously generated tumour suppressor C_18_-ceramide binds I2PP2A/SET through the hydrophobic-binding pocket, which is localized within the two anti-parallel beta sheet stabilized loops and an extended alpha helix, including the K209/Y122 residues. In addition, data revealed that I2PP2A/SET is highly expressed while C_18_-ceramide is downregulated in the majority of primary human lung tumours compared to their adjacent normal lung tissues, suggesting that I2PP2A/SET might present a key target to reactivate PP2A tumour suppressor signalling. Accordingly, we explored the therapeutic potential of targeting I2PP2A/SET using a sphingosine derivative drug, FTY720 (Fingolimod), which mimicked ceramide for binding I2PP2A/SET, leading to PP2A reactivation, and lung cancer cell death via, at least in part, RIPK1-dependent programmed necrosis, independently of its immune suppression function.

IMPACT:

The data presented here provide structural and molecular details of ceramide/FTY720-I2PP2A/SET binding via lipid–protein interaction, which in turn leads to reactivation of tumour suppressor PP2A and induction of necroptosis, resulting in lung cancer cell death. Overall, these results suggest a novel mechanism-based strategy for the inhibition of lung tumour growth via targeting I2PP2A/SET oncoprotein by a sphingosine analogue drug FTY720, indicating that future development of novel small molecule inhibitors of I2PP2A/SET might be efficacious for the treatment of lung cancers.

### PP2A activity assay

Protein lysate (100 µg) was used for PP2Ac immunoprecipitation with a phosphatase kit (Millipore), as described by the manufacturer.

## Supplementary material

Detailed facts of importance to specialist readers are published as ”Supporting Information”. Such documents are peer-reviewed, but not copy-edited or typeset. They are made available as submitted by the authors.

## References

[b1] Berdyshev EV, Gorshkova I, Skobeleva A, Bittman R, Lu X, Dudek SM, Mirzapoiazova T, Garcia JG, Natarajan V (2009). FTY720 inhibits ceramide synthases and up-regulates dihydrosphingosine 1-phosphate formation in human lung endothelial cells. J Biol Chem.

[b2] Bielawski J, Pierce JS, Snider J, Rembiesa B, Szulc ZM, Bielawska A (2010). Sphingolipid analysis by high performance liquid chromatography-tandem mass spectrometry (HPLC-MS/MS). Adv Exp Med Biol.

[b3] Billich A, Bornancin F, Devay P, Mechtcheriakova D, Urtz N, Baumruker T (2003). Phosphorylation of the immunomodulatory drug FTY720 by sphingosine kinases. J Biol Chem.

[b4] Biton S, Ashkenazi A (2011). NEMO and RIP1 control cell fate in response to extensive DNA damage via TNF-alpha feedforward signaling. Cell.

[b5] Bonapace L, Bornhauser BC, Schmitz M, Cario G, Ziegler U, Niggli FK, Schafer BW, Schrappe M, Stanulla M, Bourquin JP (2010). Induction of autophagy-dependent necroptosis is required for childhood acute lymphoblastic leukemia cells to overcome glucocorticoid resistance. J Clin Invest.

[b6] Chalfant CE, Kishikawa K, Mumby MC, Kamibayashi C, Bielawska A, Hannun YA (1999). Long chain ceramides activate protein phosphatase-1 and protein phosphatase-2A. Activation is stereospecific and regulated by phosphatidic acid. J Biol Chem.

[b7] Cho YS, Challa S, Moquin D, Genga R, Ray TD, Guildford M, Chan FK (2009). Phosphorylation-driven assembly of the RIP1-RIP3 complex regulates programmed necrosis and virus-induced inflammation. Cell.

[b8] Christofferson DE, Yuan J (2010). Necroptosis as an alternative form of programmed cell death. Curr Opin Cell Biol.

[b9] Cohen JA, Barkhof F, Comi G, Hartung HP, Khatri BO, Montalban X, Pelletier J, Capra R, Gallo P, Izquierdo G (2010). Oral fingolimod or intramuscular interferon for relapsing multiple sclerosis. N Engl J Med.

[b10] Degterev A, Hitomi J, Germscheid M, Ch'en IL, Korkina O, Teng X, Abbott D, Cuny GD, Yuan C, Wagner G (2008). Identification of RIP1 kinase as a specific cellular target of necrostatins. Nat Chem Biol.

[b11] Don AS, Martinez-Lamenca C, Webb WR, Proia RL, Roberts E, Rosen H (2007). Essential requirement for sphingosine kinase 2 in a sphingolipid apoptosis pathway activated by FTY720 analogues. J Biol Chem.

[b12] Eichhorn PJ, Creyghton MP, Bernards R (2009). Protein phosphatase 2A regulatory subunits and cancer. Biochim Biophys Acta.

[b13] Hitomi J, Christofferson DE, Ng A, Yao J, Degterev A, Xavier RJ, Yuan J (2008). Identification of a molecular signaling network that regulates a cellular necrotic cell death pathway. Cell.

[b14] Herzog F, Kahraman A, Boehringer D, Mak R, Bracher A, Waltzhoeni T, Leitner A, Beck M, Harti FU, Ban N (2012). Structural probing of a protein phosphatase 2A network by chemical cross-linking and mass spectrometry. Science.

[b15] Holler N, Zaru R, Micheau O, Thome M, Attinger A, Valitutti S, Bodmer JL, Schneider P, Seed B, Tschopp J (2000). Fas triggers an alternative, caspase-8-independent cell death pathway using the kinase RIP as effector molecule. Nat Immunol.

[b16] Karathanassis D, Stahelin RV, Bravo J, Perisic O, Pacold CM, Cho W, Williams RL (2002). Binding of the PX domain of p47(phox) to phosphatidylinositol 3,4-biphosphate and phosphatidic acis is masked by an intramolecular interaction. EMBO J.

[b17] Kudo N, Kumagai K, Tomishige N, Yamaji T, Wakatsuki S, Nishijima M, Hanada K, Kato R (2008). Structural basis for specific lipid recognition by CERT responsible for nonvesicular trafficking of ceramide. Proc Natl Acad Sci USA.

[b18] Lahiri S, Park H, Laviad EL, Lu X, Bittman R, Futerman AH (2009). Ceramide synthesis is modulated by the sphingosine analog FTY720 via a mixture of uncompetitive and noncompetitive inhibition in an Acyl-CoA chain length-dependent manner. J Biol Chem.

[b19] LaMontagne K, Littlewood-Evans A, Schnell C, O'Reilly T, Wyder L, Sanchez T, Probst B, Butler J, Wood A, Liau G (2006). Antagonism of sphingosine-1-phosphate receptors by FTY720 inhibits angiogenesis and tumor vascularization. Cancer Res.

[b20] Li M, Makkinje A, Damuni Z (1996). The myeloid leukemia-associated protein SET is a potent inhibitor of protein phosphatase 2A. J Biol Chem.

[b21] Liu Q, Alinari L, Chen CS, Yan F, Dalton JT, Lapalombella R, Zhang X, Mani R, Lin T, Byrd JC (2010). FTY720 shows promising in vitro and in vivo preclinical activity by downmodulating Cyclin D1 and phospho-Akt in mantle cell lymphoma. Clin Cancer Res.

[b22] Mukhopadhyay A, Saddoughi SA, Song P, Sultan I, Ponnusamy S, Senkal CE, Snook CF, Arnold HK, Sears RC, Hannun YA (2009). Direct interaction between the inhibitor 2 and ceramide via sphingolipid–protein binding is involved in the regulation of protein phosphatase 2A activity and signaling. FASEB J.

[b23] Muto S, Senda M, Akai Y, Sato L, Suzuki T, Nagai R, Senda T, Horikoshi M (2007). Relationship between the structure of SET/TAF-Ibeta/INHAT and its histone chaperone activity. Proc Natl Acad Sci USA.

[b24] Neviani P, Santhanam R, Oaks JJ, Eiring AM, Notari M, Blaser BW, Liu S, Trotta R, Muthusamy N, Gambacorti-Passerini C (2007). FTY720, a new alternative for treating blast crisis chronic myelogenous leukemia and Philadelphia chromosome-positive acute lymphocytic leukemia. J Clin Invest.

[b25] Ogretmen B, Hannun YA (2004). Biologically active sphingolipids in cancer pathogenesis and treatment. Nat Rev Cancer.

[b26] Paugh SW, Payne SG, Barbour SE, Milstien S, Spiegel S (2003). The immunosuppressant FTY720 is phosphorylated by sphingosine kinase type 2. FEBS Lett.

[b27] Pchejetski D, Paulmurugan R, Park S, Mickey BE, Asaithamby A, Saha D, Kelliher MA, Mukhopadhyay P, Banani F, Madden CJ (2010). FTY720 (fingolimod) sensitizes prostate cancer cells to radiotherapy by inhibition of sphingosine kinase-1. Cancer Res.

[b28] Pewzner-Jung Y, Ben-Dor S, Futerman AH (2006). When do Lasses (longevity assurance genes) become CerS (ceramide synthases)? insights into the regulation of ceramide synthesis. J Biol Chem.

[b29] Pyne S, Bittman R, Pyne NJ (2011). Sphingosine kinase inhibitors and cancer: seeking the golden sword of Hercules. Cancer Res.

[b30] Ramnarain DB, Paulmurugan R, Park S, Mickey BE, Asaithamby A, Saha D, Kelliher MA, Mukhopadhyay P, Banani F, Madden CJ (2008). RIP1 links inflammatory and growth factor signaling pathways by regulating expression of the EGFR. Cell Death Differ.

[b31] Roberts KG, Smith AM, McDougall F, Carpenter H, Horan M, Neviani P, Powell JA, Thomas D, Guthridge MA, Perrotti D (2010). Essential requirement for PP2A inhibition by the oncogenic receptor c-KIT suggests PP2A reactivation as a strategy to treat c-KIT+ cancers. Cancer Res.

[b32] Saddoughi SA, Garrett-Mayer E, Chaudhary U, O'Brien PE, Afrin LB, Day TA, Gillespie MB, Sharma AK, Wilhoit CS, Bostick R (2011). Results of a phase II trial of gemcitabine plus doxorubicin in patients with recurrent head and neck cancers: serum C-ceramide as a novel biomarker for monitoring response. Clin Cancer Res.

[b33] Salas A, Ponnusamy S, Senkal CE, Meyers-Needham M, Selvam SP, Saddoughi SA, Apohan E, Sentelle RD, Smith C, Gault CR (2011). Sphingosine kinase-1 and sphingosine 1-phosphate receptor 2 mediate Bcr-Abl1 stability and drug resistance by modulation of protein phosphatase 2A. Blood.

[b34] Senkal CE, Ponnusamy S, Manevich Y, Meyers-Needham M, Saddoughi SA, Mukhopadyay A, Dent P, Bielawski J, Ogretmen B (2011). Alteration of ceramide synthase 6/C16-ceramide induces activating transcription factor 6-mediated ER-stress and apoptosis via perturbation of cellular Ca+2 and ER/golgi membrane network. J Biol Chem.

[b35] Seo SB, McNamara P, Heo S, Turner A, Lane WS, Chakravarti D (2001). Regulation of histone acetylation and transcription by INHAT, a human cellular complex containing the set oncoprotein. Cell.

[b36] Stahelin RV, Cho W (2001). Differential roles of ionic, aliphatic, and aromatic residues in membrane-protein interactions: a surface plasmon resonance study on phospholipases A2. Biochemistry.

[b37] Sun L, Wang H, Wang Z, He S, Chen S, Liao D, Wang L, Yan J, Liu W, Lei X (2012). Mixed lineage kinase domain-like protein mediates necrosis signaling downstream of RIP3 kinase. Cell.

[b38] Thon L, Mohlig H, Mathieu S, Lange A, Bulanova E, Winoto-Morbach S, Schutze S, Bulfone-Paus S, Adam D (2005). Ceramide mediates caspase-independent programmed cell death. FASEB J.

[b39] Wallington-Beddoe CT, Hewson J, Bradstock KF, Bendall LJ (2011). FTY720 produces caspase-independent cell death of acute lymphoblastic leukemia cells. Autophagy.

[b40] Wang Z, Jiang H, Chen S, Du F, Wang X (2012). The mitochondrial phosphatase PGAM5 functions at the convergence point of multiple necrotic death pathways. Cell.

[b41] Weinlich R, Dillon CP, Green DR (2011). Ripped to death. Trends Cell Biol.

[b42] Westermarck J, Hahn WC (2008). Multiple pathways regulated by the tumor suppressor PP2A in transformation. Trends Mol Med.

[b43] Wooten-Blanks LG, Song P, Senkal CE, Ogretmen B (2007). Mechanisms of ceramide-mediated repression of the human telomerase reverse transcriptase promoter via deacetylation of Sp3 by histone deacetylase 1. FASEB J.

[b44] Yeh E, Cunningham M, Arnold H, Chasse D, Monteith T, Ivaldi G, Hahn WC, Stukenberg PT, Shenolikar S, Uchida T (2004). A signalling pathway controlling c-Myc degradation that impacts oncogenic transformation of human cells. Nat Cell Biol.

[b45] Zhang W, Yang J, Liu Y, Chen X, Yu T, Jia J, Liu C (2009). PR55 alpha, a regulatory subunit of PP2A, specifically regulates PP2A-mediated beta-catenin dephosphorylation. J Biol Chem.

