# Supplementary material for: Sphingosine analogue drug FTY720 targets I2PP2A/SET and mediates lung tumour suppression via activation of PP2A-RIPK1-dependent necroptosis
Source: EMBO Mol Med. 2012 Nov 25;5(1):105–21. doi: 10.1002/emmm.201201283 (PMC3569657; doi:10.1002/emmm.201201283)
Supplement: Supplementary file 1 [file emmm0005-0105-SD1.pdf]

EMBO Molecular Medicine

Manuscript EMM-2012-01283

Sphingosine analogue drug FTY720 targets I2PP2A/SET and mediates lung tumor suppression via activation of PP2A-RIPK1-dependent necroptosis

Sahar A. Saddoughi, Salih Gencer, Yuri K. Peterson, Katherine E. Ward, Archana Mukhopadhyay, Joshua Oaks, Jacek Bielawski, Zdzislaw M. Szulc, Raquela J. Thomas, Shanmugam P. Selvam, Can E. Senkal, Elizabeth Garrett-Mayer, Ryan M. De Palma, Dzmitry Fedarovich, Angen Liu, Amyn A. Habib, Robert V. Stahelin, Danilo Perrotti, and Besim Ogretmen

Corresponding author: Besim Ogretmen, Medical University of South Carolina

Review timeline:

Submission date:	13 February 2012
Editorial Decision:	12 March 2012
Revision received:	10 August 2012
Editorial Decision:	10 September 2012
Revision received:	26 September 2012
Accepted:	28 September 2012

Transaction Report:

(Note: With the exception of the correction of typographical or spelling errors that could be a source of ambiguity, letters and reports are not edited. The original formatting of letters and referee reports may not be reflected in this compilation.)

1st Editorial Decision 12 March 2012

Thank you for the submission of your manuscript to EMBO Molecular Medicine. We have now heard back from the three referees whom we asked to evaluate your manuscript. Although the referees find the study to be of potential interest, they also raise a number of concerns that should be convincingly addressed in a major revision.

As you will see from the enclosed reports, there are some consistencies between the different referees, especially regarding:

- the direct interaction between ceramide and FTY720 and I2PP2A that should be better demonstrated (referees 1 and 2),

- cytotoxicity and ceramide levels that should be better analysed (referees 1, 2 and 3),

- deeper investigation of the underlying mechanism that is required as suggested by referee #3 and hinted upon by referee #1.

Given these evaluations, I would like to give you the opportunity to revise your manuscript, with the

understanding that the referee concerns must be fully addressed within the space and time constraints outlined below and that acceptance of the manuscript would entail a second round of review. Please note that it is EMBO Molecular Medicine policy to allow only a single round of revision and that, as acceptance or rejection of the manuscript will depend on another round of review, your responses should be as complete as possible.
In addition, on an editorial point of view, please refer to our guidelines to provide higher figure resolution, reformat the references to our standard and copy-edit the manuscript for typos and mistakes.

Revised manuscripts should be submitted within three months of a request for revision; they will otherwise be treated as new submissions, except under exceptional circumstances in which a short extension is obtained from the editor. Also, the length of the revised manuscript may not exceed 60,000 characters (including spaces) and, including figures, the paper must ultimately fit onto optimally ten pages of the journal. You may consider including any peripheral data (but not methods in their entirety) in the form of Supplementary information.

I look forward to seeing a revised form of your manuscript as soon as possible.

Yours sincerely,

Editor
EMBO Molecular Medicine

***** Reviewer's comments *****

Referee #1:

The manuscript by Saddoughi et al follows up their previous observation that I2PP2A binds ceramide. They model the interaction based on the CERT/ceramide structure, and show binding of endogenous ceramides to I2PP2A expressed in A549 cells. They show that I2PP2A levels are often increased in lung cancers, and that the sphingolipid analogue FTY720 has efficacy in an A549 xenograft tumor model. Through a convincing set of experiments, they show that the unphosphorylated form of FTY720 binds I2PP2A, thereby stimulating PP2A activity and inhibiting tumor formation, and that while SK2 is important for generation of P-FTY720 ,particularly in plasma, it is not required for tumor suppressive effects. They demonstrate that knockdown of PP2A partially alleviates the FTY720-induced decrease in cell viability, and that myc degradation is a potentially important consequence of PP2A activation. They further demonstrate that I2PP2A knockdown mimics FTY720 treatment, and prevents further cell death by FTY720. Finally, they suggest that the decrease in cell survival is due to necrosis, as it is independent of caspases, autophagy, bax/bak, and further requires RIPK1.

Although there are some interesting findings in this article, they are not really currently presented in a coherent and in-depth manner. The first figure seems to show very similar data to what was previously published by this group. The conclusion that I2PP2A levels in lung tumors seems reasonable (although the underlying mechanism for this is not explored), but the ceramide levels (and ceramide synthase levels) seem inconclusive. While the data showing the effects of FTY720 on viability and tumor formation is compelling, as well as the role of I2PP2A and PP2A in this, the data showing direct binding of FTY720 to I2PP2A (Fig. 3), seems marginal. Finally the conclusion that the effects on cell viability are entirely through necroptosis seems premature, as the effects using inhibitors, RNAi and knockout cells is partial. The general applicability of FTY720 sould also be tested by including additional lung cancer cell line models.

Specific points:

They should show some control proteins in Fig. 3C that do not bind to FTY720.

The Fig. legend to 3D-E refers to F, which doesn't exist.

The data showing binding of FTY720 to I2PP2A in Fig. 3D-E is quite weak.

It isn't clear why binding of I2PP2A to FTY720 would decrease the amount of PP2A Y307 phosphorylation.

The statement "These data suggest that phosphorylation of FTY720 is not required to bind to I2PP2A/SET" is misleading. In fact their data suggest that FTY720 phosphorylation prevents binding to I2PP2A.

Referee #2 (Comments on Novelty/Model System):

A major shortcoming of this study is that no biophysical data on the interaction between ceramide or FTY720 and I2PP2A are provided. Binding of FTY720 was shown only at a high drug concentration (10 μM in pull-down studies and 10 mol% in lipid sedimentation). This raises serious doubts regarding the relevance of the observations. Furthermore, is is not convincingly demonstrated that FTY720 is cytotoxic at such high concentration only through binding to I2PP2A, and that its tumor-suppressive activity in vivo is indeed mediated through I2PP2A.

Referee #2 (Other Remarks):

The interaction between protein phosphatase 2A (PP2A) and its inhibitor I2PP2A/SET is supposed to be modulated by ceramide, which binds to the inhibitor. The present manuscript attempts to describe the interaction of ceramide species with I2PP2A based on molecular docking into a homology model and based on mutagenesis studies. Overexpression of I2PPA and/or downregulation of C18-ceramide is given as potential mechanism for suppression of PP2A in lung tumors. Furthermore, the drug FTY720 was identified to also bind to I2PP2A, supposedly leading to PP2A reactivation and lung cancer cell death.

A major shortcoming of this study is that no biophysical data on the interaction between ceramide or FTY720 and I2PP2A are provided. Binding of FTY720 was shown only at a high drug concentration (10 μM in pull-down studies and 10 mol% in lipid sedimentation). This raises serious doubts regarding the relevance of the observations. Furthermore, is is not convincingly demonstrated that FTY720 is cytotoxic at such high concentration only through binding to I2PP2A, and that its tumor-suppressive activity in vivo is indeed mediated through I2PP2A.

Specific points:

- The discussion of pulldown data in the 2nd half of p.6 is confusing:
* "... C18-ceramide (about 30% of total C18-ceramide) ..." should probably read "... C18-ceramide (about 30% of total ceramide) ..."

* The following sentence is not understandable:
"In addition to C18-ceramide, wt-I2PP2A/SET also bound to C20- (1.9 fold), C22- (2.6 fold), C24- (2.1 fold) and C26-ceramide (2.5 fold) compared to the K209D-I2PP2A/SET in A549 cells (Supplemental Figure S3)." When using the "% of elution"-values in Suppl Fig. S and dividing the wt-I2PP2A and the vector values, one obtains these fold-increases (but that it is an increase is not mentioned in the sentence). But this is not a comparison to the K209D-I2PP2A mutant, as stated in the sentence.

© EMBO

* Also the general point is raised which parameter should be used to define "binding": the absolute amount of eluted ceramide (as done in Fig.1D), or the elution ratio (E/T) over vector values, and with which rationale.

- The study on the I2PPA mutants suffers from the fact that is unknown if they are all expressed to the same extent as the wt-protein.

- The sentence " ...Y122C-I2PP2A/SET increased binding to C18-, C20-, C22-, and C26-ceramide around 2.5, 4.2, 4.6, 3.2-, and 2.4-fold, respectively (Supplemental Figure S3)." is unclear - increased binding compared to what and using which parameter?
- According to Suppl. Fig. S3 Wt-I2PP2A directed to the ER binds C16 and a low amount of C18, while expression in the nucleus leads to binding of C18 and no binding of C16. Does this correlate with the expression of the various ceramide species in these compartments?
- In Fig. 3D it remains unclear what "fold-change in binding" on the y-axis means (what is compared to what?).

- A concentration of FTY720 of 10 µM is regarded as physiological by the authors - what is the evidence for this? In the animal experiment, serum levels of FTY720 were 0.1 µM (Fig.4D) and tumor levels are only reported per nmol Pi (so abolute concentration remains elusive). Actually, in the absence of evidence for a PP2A-mediated effect, Fig. S9 shows that FTY720 is cytotoxic to MEFs (i.e., non-cancer cells) at 10 µM by whatever mechanism.

- What is the absolute amount of FTY720 binding to I2WT in Fig.3E?

- In Fig.7A, FTY720 is shown to be unable to increase LDH release when I2PP2A is silenced; but cells are apparently already dying because of the silencing so one must not necessarily expect an additional effect by FTY720. A control for a non-targeting shRNA is missing, so LDH release may simply be due to the transduction procedure.

- Data in Fig. 7C and D are claimed to show that FTY720 works in tumor suppression via I2PP2A. But FTY720 actually suppresses tumor growth in any case, so this conclusion cannot be drawn.

Referee #3:

In the article entitled "Sphingosine analogue drug FTY720 directly binds/targets I2PP2A/SET and mediates lung tumor suppression via activation of PP2A-RIPK1- dependent tumor necroptosis", Saddoughi et al. characterize a signaling cascade that bridges I2PP2A/SET inhibition by the clinically employed sphingosine analogue FTY720 to activation of protein phosphatase 2A (PP2A) and cancer cell death via regulated necrosis. The authors applied a highly diversified approach and employed a variety of in silico, in vitro and in vivo models, including structural modeling, studies on human and murine cell lines, assays on xenografts and assessments on patient material. The authors provide a very robust dataset supporting the contention that FTY720 can exert oncosuppressive functions by directly binding to I2PP2A/SET and hence de-inhibiting PP2A.

Dealing with the molecular mechanisms whereby a drug that is approved for the treatment of multiple sclerosis (FTY720) may be used as an off-label medication for anticancer therapy, the report by Saddoughi et al. falls well within the editorial scope of EMBO Molecular Medicine. The authors approached the scientific question in a very appropriate way, employing a variety of complementary models and undertaking a high number of well-targeted experiments. This said, the authors should resolve the following major concerns for the article to be considered for publication in EMBO Molecular Medicine.

1. I'm not convinced by the fact that the phosphorylation of FTY720 by SK2 is really dispensable for the regulation of the PP2A/Myc axis (even though I am convinced by the binding data shown in Figure 5A). First, in Figure 5C, Myc degradation is not fully prevented by the absence of SK2. Second, in Figure 6D, the effects of FTY720 on SK2-/- mice are too small to confidently conclude that FTY720 is entirely efficient in this context. In addition, in this set of experiments, the authors employed SK2-/- mice but LLC allografts, which (to my knowledge) express SK2. The paper would be ameliorated if the authors investigate in more details and hence clarified this issue. Among other experiments, the authors might evaluate the growth of SK2-deficient versus SK2-proficient cells responding to FTY720 in SK2-deficient versus SK2-proficient animals.

2. In several instances, for example (but not only) in Figure 6E versus 6F versus 7B, FTY720 is shown to induce cell death to quite variable extents. This is problematic as it constitutes a control condition for the interpretation of several other results.

3. In Figure 5G,H, the authors report that MycT58A cannot be degraded upon the administration of FTY720 as the wild-type variant does, and that this provides some extent of cytoprotection. Do the authors suggest that Myc degradation is required for cell death as induced for FTY720? If so, how do the authors link this to the RIPK1-dependent pathway elucidated in the last sections of the paper? Would Myc act upstream or downstream RIPK1? This is a critical point that must be clarified.

4. Recent work from Xiaodong Wang's group has elucidated the signaling pathways that operate downstream of RIPK1/RIPK3 for the execution of regulated necrosis. The authors should investigate whether FTY720 induces indeed RIPK3-, MLKL- and PGAM5-dependent regulated necrosis.

5. It would be interesting to know the abundance of other ceramide species in NSCLC patients, as the authors measured only C18-ceramide but I2PP2A/SET also binds other ceramide.

Additional issues that must be addressed include:

1. Electron microscopy data in Figure 4C must be provided in quantitative terms (for instance % of cells with disrupted mitochondria in samples of 100-150 cells per condition)

2. Serum concentration in Figures 4D and 5F should be expressed with standard units (pM or ng/mL)

3. Statistical comparisons are missing from several figures, and should be included

4. PP2A overexpression in Figure 6F must be demonstrated by immunoblotting

5. The sentence "Importantly, knockdown of I2PP2A/SET prevented FTY720-induced..." does not reflect the data presented in Figure 7A. I assume the authors wanted to say "Importantly, knockdown of FTY720 is ineffective in cells lacking I2PP2A/SET as a result of shRNA-mediated knockdown"

6. Atg5-/- MEFs are equally sensitive to FTY720 than their WT counterparts, which is relatively rare, as these cells are normally more sensitive to cell death induction. The authors should check that their Atg5-/- MEFs are more sensitive to the induction of cell death by apoptotic stimuli.

7. Elevated LDH in the supernatant is not a measure of necrosis, meaning primary necrosis. This is conceptually important and must be clarified: LDH release is a measure of plasma membrane breakdown, which occurs early during regulated necrosis or late during apoptosis (secondary necrosis).

8. The authors should have the article corrected by a native English speaker, as several grammatical mistakes, incorrect sentences and typos are scattered throughout the text

9. On page 6, the discussion on ceramide binding should be made by always keeping as a comparison term the WT I2PP2A/SET protein

10. The first two sections of the paper can easily be merged into a single one, for the sake of synthesis.

11. The binding data relative to ER-I2PP2A/SET in situ can simply reflect the local abundance of distinct ceramide species. The authors should perform in vitro studies to clarify this issue.

1st Revision - Authors' Response 10 August 2012

Thank you for your letter of March 12, 2012, regarding the review of our manuscript (EMM-2012-01283). We would like to thank the Reviewers for their careful and positive review. We are pleased that the referees found the study to be of potential interest, and the revision was invited for further consideration. We also thank you for extending our deadline for submission of the revised manuscript, which required a substantial amount of additional experiments to address points raised in previous review.

We have revised the manuscript to address the Reviewers' comments, and are now submitting the amended manuscript entitled "Sphingosine analogue drug FTY720 targets I2PP2A/SET and mediates lung tumor suppression via activation of PP2A-RIPK1-dependent tumor necroptosis" to be considered for publication in *EMBO Molecular Medicine*.

Please find below our point-by-point response to the comments raised by the Reviewers:

Reviewer 1:

We thank the Reviewer 1 for finding our experiments convincing and interesting. We have addressed the points raised by the Reviewer as follows:

1. The Reviewer mentioned that the first figure seems to show very similar data to what was previously published.

RESPONSE: Molecular modeling of ceramide-I2PP2A/SET binding, or effects of subcellular localization of I2PP2A/SET on endogenous ceramide binding preference, which are now shown in Fig. 1, have not been shown previously. We believe that these novel data will be informative for investigators who are interested in structural and functional details of I2PP2A/SET-ceramide interaction.

2. It was pointed out that the data showing direct binding of FTY720 to I2PP2A seem marginal.

RESPONSE: To address this point, additional binding studies were performed using purified human I2PP2A/SET protein and FTY720, P-FTY720 and C_{18}-ceramide by surface plasmon resonance (SPR). These data are now included in Fig. 3E-F and Table 1.

3. The Reviewer stated that the conclusion that the effects on cell viability are entirely through necroptosis seems premature, as the effects using inhibitors, RNAi and knockout cells is partial.

RESPONSE: We agree with the Reviewer, and we now rephrase our conclusions to convey the point throughout the revised manuscript that the effects of FTY720 on the inhibition of cell viability are **in part** regulated through necroptosis, which might be context/cell/tumor type dependent.

4. The Reviewer mentioned that the general applicability of FTY720 should also be tested by including additional lung cancer cell line models.

RESPONSE: We agree, and to address this point, other human lung cancer cell lines H157 and H827 were utilized in our additional experiments. These data are now shown in Supplemental Figure S12A-C.

Specific points:

1. It was requested that some control proteins that do not bind to FTY720 should be included in binding studies.

RESPONSE: To address this, in our new binding studies, we utilized purified $p47^{phox}$-PX which binds $PI(3,4)P_2$ and phosphatidic acid, but not sphingolipids, as an additional control in SPR assays. These data are now shown in Table 1.

2. The Reviewer pointed out that the Fig. legend to previous 3D-E refers to F, which did not exist.

RESPONSE: Figure legend has been corrected for revised Fig. 3.

3. The Reviewer raised an important point that the data showing binding of FTY720 to I2PP2A in Fig. 3D-E is quite weak.

RESPONSE: To address this point, we have performed additional binding studies using purified I2PP2A/SET in SPR binding studies, and the data are now shown in Fig. 3E-F and Table 1.

4. The Reviewer mentioned that it is not clear why binding of I2PP2A to FTY720 would decrease the amount of PP2A Y307 phosphorylation.

RESPONSE: We agree with the Reviewer that I2PP2A/SET is not known to regulate Y307 phosphorylation of PP2A. However, it is well established that phosphorylation of PP2A at Y307 is an inactive form of the phosphatase. Thus, in our studies, we detected Y307 phosphorylation of PP2A to monitor its basal activity status in lung tissues. This point is now clarified in the text.

5. It was pointed out that the statement "these data suggest that phosphorylation of FTY720 is not required to bind to I2PP2A/SET" was misleading. In fact their data suggest that FTY720 phosphorylation prevents binding to I2PP2A.

RESPONSE: We agree, and this statement is now clarified in the text.

Reviewer 2:

We thank the Reviewer 2 for his/her careful review. We have addressed the points raised by the Reviewer as follows:

1. The Reviewer pointed out that a major shortcoming of this study is that no biophysical data on the interaction between ceramide or FTY720 and I2PP2A are provided.

RESPONSE: To address this point, additional binding studies were performed using purified I2PP2A/SET protein and FTY720, P-FTY720 and C_{18}-ceramide. These data are now included in Fig. 3E-F and Table 1.

Specific points:

1. It was mentioned that the discussion of pulldown data in the 2nd half of p.6 is confusing.

RESPONSE: This is now clarified in the revised text.

2. The Reviewer stated that the study on the I2PP2A mutants suffers from the fact that it is unknown if they are all expressed to the same extent as the wt-protein.

RESPONSE: We have addressed this point by providing Western blots to demonstrate the expression levels of these proteins in Supplemental Figure S3.

3. The Reviewer pointed out that the sentence " ...Y122C-I2PP2A/SET increased binding to C18-, C20-, C22-, and C26-ceramide around 2.5, 4.2, 4.6, 3.2-, and 2.4-fold, respectively (in revised Supplemental Figure S2)." is unclear - increased binding compared to what and using which parameter?

RESPONSE: To clarify this point, we have stated in the revised text that these numbers were obtained in comparison to vector-transfected controls.

4. It was mentioned that according to Suppl. Fig. S3 Wt-I2PP2A directed to the ER binds C16 and a low amount of C18, while expression in the nucleus leads to binding of C18 and no binding of C16. Does this correlate with the expression of the various ceramide species in these compartments?

RESPONSE: To address this point, we measured ceramide levels in cytoplasm versus nuclei-enriched A549 cell fractions. The data showed that nuclear versus cytoplasmic

levels of C18 versus C16-ceramides do not correlate with their binding preference to wt-I2PP2A or its ER-mutant. Thus, these data suggest that ceramide-binding selectivity is mainly regulated by the subcellular localization of I2PP2A/SET and availability of ceramides, rather than their fatty acid chain lengths, in ER versus nuclear membranes. These data are now shown in Supplemental Figure S4.

5. The Reviewer mentioned that in Fig. 3D it remains unclear what "fold-change in binding" on the y-axis means (what is compared to what?).

RESPONSE: This point is now clarified in the revised text.

6. It was pointed out that a concentration of FTY720 of 10 μM is regarded as physiological by the authors - what is the evidence for this?

RESPONSE: This is now corrected in the text.

7. It was asked what the absolute amount of FTY720 binding to I2WT was in Fig.3E?

RESPONSE: These data are now provided in Supplemental Figure S2.

8. The Reviewer pointed out that in Fig.7A, FTY720 is shown to be unable to increase LDH release when I2PP2A is silenced; but cells are apparently already dying because of the silencing, so one must not necessarily expect an additional effect by FTY720. A control for a non-targeting shRNA is missing, so LDH release may simply be due to the transduction procedure.

RESPONSE: We have performed these studies using vector-transfected controls, which had no effect on LDH release. This is now clarified in revised text.

9. The Reviewer mentioned that data in Fig. 7C and D are claimed to show that FTY720 works in tumor suppression via I2PP2A. But FTY720 actually suppresses tumor growth in any case, so this conclusion cannot be drawn.

RESPONSE: This is now clarified and corrected in revised text.

Reviewer 3:

We thank the Reviewer 3 for stating that “the authors provide a very robust dataset supporting the contention that FTY720 can exert oncosuppressive functions by directly binding to I2PP2A/SET and hence de-inhibiting PP2A”. We are also pleased that the Reviewer 3 pointed out that “the authors approached the scientific question in a very appropriate way, employing a variety of complementary models and undertaking a high number of well-targeted experiments”. We have addressed the points raised by the Reviewer as follows:

1. The Reviewer stated that the fact that the phosphorylation of FTY720 by SK-2 is really dispensable for the regulation of the PP2A/Myc axis was not very clear.

RESPONSE: To address this point, we performed additional experiments using sh-SK-2/A549 cells for stable knockdown of SK-2. These cells compared to Scr-shRNA/A549 cells were treated with FTY720 or vehicle controls, and the data showed that knockdown of SK-2, which reduces phosphorylation of FTY720, had no preventive effects, and resulted in an increase in cell death compared to controls, suggesting that phosphorylation of FTY720 by SK-2 is not required for it's cell killing effects at least in these lung cancer cells. These data are now included in the revised manuscript (Supplemental Fig. S9B-C)

2. It was mentioned that in several instances, for example (but not only) in Figure 6E versus 6F versus 7B, FTY720 is shown to induce cell death to quite variable extents. This is problematic as it constitutes a control condition for the interpretation of several other results.

RESPONSE: We apologize for not making these data more clear. Cells used in studies shown in 6E/6F are different than those in 7B. The cells in 7B are sh-I2PP2A/A549 cells, which have been reconstituted with vector, WT-I2PP2A or I2PP2A-mutants. In this setting, we found that cells that lack I2PP2A, but have vector control reconstitution do not respond to FTY720. Therefore, we do not see an increase in LDH/cell death in this setting. In Fig. 6E, cells are A549 cells that have siRNA scrambled or PP2Ac RNAi. Since these cells have I2PP2A present, they are more responsive to FTY720, however, the lack of PP2A seemed to attenuate cell killing effects of FTY720, as expected.

3. The Reviewer pointed out that in Figure 5G,H, the authors report that MycT58A cannot be degraded upon the administration of FTY720 as the wild-type variant does, and that this provides some extent of cytoprotection. Do the authors suggest that Myc degradation is required for cell death as induced for FTY720? If so, how do the authors link this to the RIPK1-dependent pathway elucidated in the last sections of the paper? Would Myc act upstream or downstream RIPK1? This is a critical point that must be clarified.

RESPONSE: We agree, and to clarify this point, we have performed additional studies, in which effects of c-Myc knock-down on necroptosis in the absence/presence of FTY720 were examined. The data showed that c-Myc knockdown by itself had no effect on necroptosis. These data suggest that endogenous c-Myc may not be necessary for the regulation of necroptosis. However, expression of T58A-c-Myc mutant appeared to have protective effects against FTY720-mediated necroptosis. Therefore, based on these data, it is difficult to judge whether c-Myc plays a role upstream or downstream of FTY720 for regulation of necroptosis. Thus, for clarity, we elected to remove T58A-c-Myc related data from the revised manuscript, and focus the manuscript on PP2A/RIPK1-dependent necroptosis. The possible relationship between c-Myc and necroptosis regulation needs to be clarified in depth future studies.

4. It was pointed out that recent work from Xiaodong Wang's group has elucidated the signaling pathways that operate downstream of RIPK1/RIPK3 for the execution of regulated necrosis. The authors should investigate whether FTY720 induces indeed RIPK3/MLKL- and PGAM5/Drp1-dependent regulated necrosis.

RESPONSE: To address this point, we have performed additional experiments. First, we found that siRNA-mediated knockdown of RIP3 had no protective effects against FTY720-induced necroptosis. These data suggest that RIP3/MLKL axis might not be involved in this process. Then, we have examined whether knock-down of Drp1, which is a down-stream target of PGAM5 affects FTY720-mediated necroptosis. Our data showed that knockdown of Drp1 had no protective effects on this process, suggesting that PGAM5/Drp1 axis might not be involved. These data are now presented in Supplemental Figure S13B-D. Based on these data, it can be speculated that FTY720 might activate RipK1 via affecting its dimerization, which is important for its activation, and/or phosphorylation on its kinase domain, as it was shown to be necessary for FTY720-mediated necroptosis in our experiments (Fig. 9B).

5. The Reviewer mentioned that it would be interesting to know the abundance of other ceramide species in NSCLC patients, as the authors measured only C18-ceramide but I2PP2A/SET also binds other ceramides.

RESPONSE: These data are now included in the revised manuscript (Supplemental Figure S5).

Additional points:

1. The Reviewer pointed out that Electron microscopy data in Figure 4C must be provided in quantitative terms (for instance % of cells with disrupted mitochondria in samples of 100-150 cells per condition).

RESPONSE: This is now provided in the revised manuscript.

2. It was mentioned that serum concentration in Figures 4D and 5F should be expressed with standard units (pM or ng/mL).

RESPONSE: This is now corrected as suggested.

3. It was pointed out that statistical comparisons are missing from several figures, and should be included.

RESPONSE: These are now included.

4. The Reviewer asked that PP2A overexpression in Figure 6F must be demonstrated by immunoblotting.

RESPONSE: This is now included in the revised manuscript (Supplemental Figure S10A).

5. It was pointed out that the sentence "Importantly, knockdown of I2PP2A/SET prevented FTY720-induced..." does not reflect the data presented in Figure 7A.

RESPONSE: This is now clarified in the text.

6. The Reviewer mentioned that Atg5-/- MEFs are equally sensitive to FTY720 than their WT counterparts, which is relatively rare, as these cells are normally more sensitive to cell death induction. The authors should check that their Atg5-/- MEFs are more sensitive to the induction of cell death by apoptotic stimuli.

RESPONSE: To address this, we have performed additional experiments, and treated WT MEF's, ATG5-/- MEF's and A549 cells with Taxol (80 nM, 24 h) or vehicle control, and then measured caspase 3 activity. We found that in this setting ATG5-/- had a greater increase in caspase 3 activity from baseline than WT MEF's, suggesting that Atg5-/- MEFs are more sensitive to apoptosis than controls. These data are now shown in Supplemental Figure S11C.

7. It was pointed out by the Reviewer that Elevated LDH in the supernatant is not a measure of necrosis, meaning primary necrosis. This is conceptually important and must be clarified: LDH release is a measure of plasma membrane breakdown, which occurs early during regulated necrosis or late during apoptosis (secondary necrosis).

RESPONSE: This is now clarified in the revised text.

8. The Reviewer suggested that the article should be corrected by a native English speaker, as several grammatical mistakes, incorrect sentences and typos are scattered throughout the text.

RESPONSE: The manuscript is edited by Dr. Jennifer Schnellman.

9. The Reviewer suggested that on page 6, the discussion on ceramide binding should be made by always keeping as a comparison term the WT-I2PP2A/SET protein.

RESPONSE: This was corrected, as suggested.

10. The Reviewer suggested that the first two sections of the paper can easily be merged into a single one, for the sake of synthesis.

RESPONSE: Because first two figures show endogenous association between ceramide and I2PP2A/SET and clinical relevance of ceramide and I2PP2A in lung tumors, respectively, we elected to keep them as separate figures for clarity.

11. It was pointed out that the binding data relative to ER-I2PP2A/SET in situ can simply reflect the local abundance of distinct ceramide species. The authors should perform in vitro studies to clarify this issue.

RESPONSE: This point is now addressed by providing additional data, as shown in Supplementary Figure S4B, which showed that ER/I2PP2A/SET binds ceramide similarly as WT-I2PP2A/SET *in vitro*.

In summary, we thank the Reviewers and editorial team for their positive review and constructive comments. We are very excited about our novel data presented in this manuscript. We hope that the revised manuscript will meet the criteria for publication in the *EMBO Molecular Medicine*.

Thank you for your consideration.

2nd Editorial Decision 10 September 2012

Thank you for the submission of your revised manuscript "Sphingosine analogue drug FTY720 targets I2PP2A/SET and mediates lung tumor suppression via activation of PP2A-RIPK1-dependent necroptosis" to EMBO Molecular Medicine. We have now received the enclosed reports from the referees whom we asked to re-assess it.

As you will see, the Reviewers acknowledge that the manuscript was significantly improved during revision and two Reviewers feel that the manuscript is suitable for publication. However, Reviewer #1 still raises concerns that should be convincingly addressed. Since we do acknowledge the potential interest of your findings, we would be willing to consider a revised manuscript with the understanding that the referee concerns must be convincingly and conclusively addressed.

Specifically, Reviewer #1 highlights that, in addition to technical concerns, the modeling should be improved.

Revised manuscripts should be submitted latest within three months of a request for revision; they will otherwise be treated as new submissions, unless arranged otherwise with the editor.

I look forward to seeing a revised form of your manuscript as soon as possible.

Editor
EMBO Molecular Medicine

***** Reviewer's comments *****

Referee #1 (Comments on Novelty/Model System):

The novelty and medical impact are both high, because if the data and model is correct, it could suggest a use for an approved drug in treating cancer. However, it is not clear form their data how universal this finding is (although they do provide some data on an additional two cell lines), some of the data is not very convincing/robust, and the data doesnt seem to completely fit their model. Moreover, after discussing their model for ceramide/I2PP2A binding with an expert in computational structural biology, there are some issues that decrease enthusiasm for the rest of the manuscript, because as written, the modeling provides the basis for their subsequent experiments. They could potentially downplay the modeling aspect and concentrate on the empirical data, which was shown in a previous paper anyway.

Referee #1 (Other Remarks):

In the revised manuscript, Saddoughi et al provide some additional SPR data showing that I2PP2A binds to PC:PE (80:20) vesicles with an affinity of 70 nM, to the same vesicles with 5% FTY720 or C18 ceramide with an affinity of 11 nM, and undetectable binding when these vesicles were supplemented with 5% phosphorylated FTY720 (termed P-FTY720 and FTY720P in the text). How such a small amount of phosphorylated FTY720 can abolish the interaction with PC:PE is not explained. They have also removed some experiments using T58A myc, and added a couple of extra cell lines to show that the effects of FTY720 on viability could be a general effect through RIP1K and PP2A activation. Therefore, the manuscript is improved over the initial submission. However, I still have some issues which reduce my enthusiasm for acceptance at this time.

1. The modeling proposed for ceramide on I2PPA seems less than ideal to me. I am not familiar with the SAPS ProCam score that they mention. The link given in the methods goes to a Virginia Tech website but there is no other documentation available from that site. The authors need to provide more details of what this analysis does. Have they tried any docking with ceramide + CERT to see if their method can reproduce this experimental structure? There are some significant differences between CERT and I2PP2A that question their model - ceramide binds to cert with the

two aliphatic chains packed against each other; the I2PP2A model has the alkyl side chains splayed out in opposite directions, and the polar interactions of ceramide and CERt are nicely buried in the pocket (productive binding energy) whereas in I2PP2A the interactions with K209 are on the surface and would have to compete with solvent (less favorable). Additional mutants besides K209D and Y1222C could be designed that would test their binding model.

2. Why is there a 1000-fold difference in the binding affinity of I2PP2A to FTY720 using pull-down and SPR?

3. It is unclear why overexpression of WT I2PP2A in Fig. 7A does not restore viability, as it reduces PP2A activity beyond normal conditions (shown in Fig. 6B). This result suggests that the effects of sh I2PP2A on cell viability is independent of its effects on PP2A activity.

4. Similarly, why doesn't PP2A overexpression by itself decrease cell viability if the death induced by FTY720 is due to increased PP2A activity. They should also document the level of PP2A overexpression using a PP2A ab in addition to the HA tag antibody.

5. Figs 7A and 7B show very marginal effects, and the data is difficult to interpret, perhaps due to excessive normalization. It might be easier to interpret this data is the raw numbers are shown.

Minor points which should also be addressed before publication:

1. In Fig. 1C why are some cells devoid of calnexin staining?

2. The term 'percentage' in the table in Fig. 2E is misleading as this normally refers to something that scores 0-100.

Referee #2 (Comments on Novelty/Model System):

After revision, this manuscript is substantially improved, in particular by providing biophysical data on the I2PP2A interaction with FTY720 and ceramide. A number of missing controls have now been included now, and unclear passages have been adequately revised.

Referee #2 (Other Remarks):

After revision this manuscript is substantially improved, in particular by providing biophysical data on the I2PP2A interaction with FTY720 and ceramide. A number of missing controls have now been included now, and unclear passages have been adequately revised.

Referee #3:

The authors have done a remarkable job in addressing (most of) the reviewers' critiques.

2nd Revision - Authors' Response 26 September 2012

Thank you for your letter of September 10, 2012, regarding the review of our manuscript (EMM-2012-01283-V2). We would like to thank the Reviewers for their careful reviews. We are pleased that the Reviewers #2 and #3 felt that the revised version of the manuscript was suitable for publication. We also thank you and the editorial board for acknowledging the potential interest of our findings and for your invitation to consider a revised manuscript after addressing the additional and valid points raised by Reviewer #1.

We have re-revised the manuscript to address the Reviewer #1's comments, and are now submitting the amended manuscript entitled "Sphingosine analogue drug FTY720 targets I2PP2A/SET and mediates lung tumor suppression via activation of PP2A-RIPK1-dependent tumor necroptosis" to be considered for publication in *EMBO Molecular Medicine*.

Please find below our point-by-point response to the comments raised by the Reviewer #1:

We thank the Reviewer 1 for finding the novelty and medical impact of our work high, and stating that "if the data and model is correct, it could suggest a use for an approved drug in treating cancer". We have addressed the points raised by the Reviewer as follows:

1. The Reviewer raised a new and valid point about the modeling of ceramide and I2PP2A/SET compared to ceramide and CERT binding. Specifically, it was mentioned that the modeling proposed for ceramide on I2PPA/SET seems less than ideal, and the SAPS ProCam score was not very clear.

RESPONSE: We agree that although there are some structural similarities between binding sites of CERT and I2PP2A/SET to ceramide, there are also major differences, as pointed out by the Reviewer: Ceramide binds to CERT with the two aliphatic chains packed against each other. The I2PP2A model has the alkyl side chains splayed out in opposite directions. Moreover, the polar interactions of ceramide and CERT are nicely buried in the pocket (productive binding energy) whereas in I2PP2A the interactions with K209 are on the surface and would have to compete with solvent (less favorable). These points are now clearly discussed in the text of the revised manuscript (p. 21, lines 15-23).

In addition, based on these points, we have elected to remove supplemental data, which attempted to highlight similarities between ceramide binding sites of CERT and I2PP2A analyzed using SAPS ProCam scores (previous Figures S1A-C). Instead, we now present our modeling and simulation studies, which were supported by site-directed

mutagenesis and binding studies for analyzing the association between ceramide or FTY720 and I2PP2A/SET. It should be noted that we refer to this binding site on I2PP2A/SET as "putative ceramide/FTY720 binding site" throughout the manuscript. This site will be examined more specifically using crystallization studies in our future experiments. It should also be noted that, despite some disadvantages, molecular modeling/simulation studies have been used for examining the association between S1P and HDAC1 (Hait, NC et al., Science, 2009) or ceramide and LC3B-II (Sentelle, RD et al., Nature Chemical Biology, 2012, in press), recently.

2. The Reviewer asked why there is a 1000-fold difference in the binding affinity of I2PP2A to FTY720 using pull-down and SPR?

RESPONSE: SPR studies measure the interaction between lipid and purified proteins directly using a single step ultra sensitive binding reaction and methodology, in which lipid moleculesare coated on an active surface, allowing Kd calculations. However, analyzing lipid protein interactions using column purification followed by Western blotting or LC/MS/MS requires many stringency washing steps, which together, result in loss of proteins and/or lipids during the binding analysis without Kd values. Therefore, it is expected that SPR is much more sensitive than other methods for quantitative analysis of lipid/protein binding, in which specific Kd values can be calculated.

3. The Reviewer stated that it is unclear why overexpression of WT I2PP2A in Fig. 7A does not restore viability, as it reduces PP2A activity beyond normal conditions (shown in Fig. 6B). It was also stated that "this result suggests that the effects of sh-I2PP2A on cell viability are independent of its effects on PP2A activity".

RESPONSE: It is shown in Fig. 7A that overexpression of WT/I2PP2A increases the response of A549/sh-I2PP2A cells to FTY720, due to reconstitution of its direct target, which is the main point of Fig. 7A, performed in situ at short term treatment (24 h). As for the effects of WT/I2PP2A overexpression alone on cell viability, data shown in Fig. 7C-E, which were performed *in vivo*, showed that overexpression of I2PP2A restored tumor proliferation significantly as compared to sh-I2PP2A xenograft-derived tumors, as well as LDH release to the serum of animals. These data support our overall hypothesis which is consistent throughout the whole manuscript, in which many different models, cell lines, molecular and pharmacological tools were used to test the overall hypothesis. It should also be noted that we repeatedly mention in the manuscript that activation of PP2A/RIK1-mediated necroptosis is in part involved in FTY720-mediated tumor suppression, and that there are other mechanisms involved in this process based on cell type and/or context.

4. The Reviewer also asked "why doesn't PP2A overexpression by itself decrease cell viability if the death induced by FTY720 is due to increased PP2A activity".

RESPONSE: PP2A conforms over 150 interprotein and 570 intraprotein complexes (Herzog, F. et al. Science, 2012). Therefore, it is difficult to interpret the effects of overexpression of catalytic subunit of PP2A itself on cell viability. Based on this, our

data shown in Fig. 6F suggest that overexpression of PP2Ac further induces FTY720-mediated LDH release, as expected. However, it is unknown which specific PP2A interprotein or intraprotein complexes are involved in its tumor suppressor function, after relieved from I2PP2A/SET in response to FTY720. These data suggest that FTY720 might not only relieve PP2Ac from the inhibitor, but it might also induce the anti-tumor activity via mediating specific interprotein or intraprotein complexes of PP2A. This point is also discussed in the text (p. 15 and 16, lines 22-23 and 1-8, respectively). The involvement of PP2A activation was also shown using okadaic acid and siRNAs to target PP2A, both of which prevented FTY720-mediated necroptosis in lung cancer cells (Fig. 6C and 6E, respectively).

5. The Reviewer mentioned that Figs 7A and 7B show very marginal effects, and the data is difficult to interpret, perhaps due to excessive normalization.

RESPONSE: We believe that data shown in Figs. 7A and 7B show that reconstitution of WT/I2PP2A restores FTY720-mediated necroptosis in A549 cells in culture even after a short-term FTY720 treatment. Importantly, these data were further validated using animal studies, in which long-term FTY720 treatment resulted in more robust effects on tumor suppression against A549 xenograft-derived tumors (shown in Figs. 7C-E). These data support our overall model and hypothesis, as mentioned above.

In addition, minor points mentioned by the Reviewer regarding: 1) Fig. 1C, in which there was an error in one of the panels for calnexin versus I2PP2A staining, and 2) Misleading Table for scoring of tumor microarray staining shown in Fig. 2E is now corrected.

In summary, we thank the Reviewers and editorial team for their positive review and constructive comments. We are very excited about our novel data presented in this manuscript. We hope that the revised manuscript will meet the criteria for publication in the *EMBO Molecular Medicine*.

Thank you for your consideration.
