# Supplementary material for: Sphingosine analogue drug FTY720 targets I2PP2A/SET and mediates lung tumour suppression via activation of PP2A-RIPK1-dependent necroptosis
Source: EMBO Mol Med. 2012 Nov 25;5(1):105–21. doi: 10.1002/emmm.201201283 (PMC3569657; doi:10.1002/emmm.201201283)
Supplement: Supplementary file 2 [file emmm0005-0105-SD2.pdf]

Sphingosine analogue drug FTY720 targets I2PP2A/SET and mediates lung tumor suppression via activation of PP2A-RIPK1-dependent necroptosis

Sahar A. Saddoughi, Salih Gencer, Yuri K. Peterson, Katherine E. Ward, Archana Mukhopadhyay, Joshua Oaks, Jacek Bielawski, Zdzislaw M. Szulc, Raquela J. Thomas, Shanmugam P. Selvam, Can E. Senkal, Elizabeth Garrett-Mayer, Ryan M. De Palma, Dzmitry Fedarovich, Angen Liu, Amyn A. Habib, Robert Stahelin, Danilo Perrotti, and Besim Ogretmen

Supporting Information Table of Contents

Supporting Information Materials and Methods pp. 1-6

Supporting Information Figure Legends pp. 7-11

Supporting Information Figure 1 pp. 12
Supporting Information Figure 2 pp. 13
Supporting Information Figure 3 pp. 14
Supporting Information Figure 4 pp. 15
Supporting Information Figure 5 pp. 16
Supporting Information Figure 6 pp. 17
Supporting Information Figure 7 pp. 18
Supporting Information Figure 8 pp. 19
Supporting Information Figure 9 pp. 20
Supporting Information Figure 10 pp. 21
Supporting Information Figure 11 pp. 22
Supporting Information Figure 12 pp. 23
Supporting Information Figure 13 pp. 24
Supporting Information Figure 14 pp. 25
Supporting Information Figure 15 pp. 26
Supporting Information Figure 16 pp. 27

Supplementary Information

MATERIALS AND METHODS

Cell lines and reagents

A549, H157, and H827 human lung cancer cells and LLC mouse lung cancer cells were grown in DMEM containing 10% FBS (Cellgro) and 1% penicillin/streptomycin (Cellgro) H1650 human lung cancer cells were grown in RPMI with 10% FBS and 1% P/S. WT, SK1-/-, SK2-/-, Bax/Bak-/-, Atg5-/-, RIP1-/-, caspase 3/7-/- MEF's were grown in DMEM with 10% FBS and 1% P/S. Cells were treated with the following reagents: FTY720, P-FTY720, and Biotin-FTY720 (Cayman Chemical Company), okadaic acid (Calbiochem), ZVAD-FMK (R&D systems) and necrostatin (Sigma).

Western blotting, IHC and confocal microscopy

Proteins were analyzed by Western blotting using the following antibodies: the rabbit polyclonal anti-I2PP2A (Globozyme), β-actin (Sigma, St. Louis, MO), anti-GFP (Abcam Inc., Cambridge, MA), rabbit polyclonal anti-eGFP for LUV binding assays (Thermo Scientific), cMyc (Santa Cruz, 9E10, sc-40), V5 (Invitrogen), HA (Cell Signaling, 6E2), RIP (BD Biosciences, 610458). The following antibodies were used for IHC staining of tumor tissues: I2PP2A/SET (Santa Cruz Bio. H-120), cMyc (Santa Cruz Bio. 9E10), PP2A total (Epitomics), p-PP2A Y307 (Epitomics).

WT-I2PP2A/SET-GFP or mutant I2PP2A/SET-GFP were expressed in A549 cells and cellular localization of these proteins was examined by immunofluorescence using Leica confocal microscopy. Rabbit polyclonal anti-calnexin (Santa Cruz Bio.) antibody in

1% BSA for another hour. Following that, rhodamine-conjugated donkey anti-rabbit IgG (Jackson Immuno Research) was used as a secondary antibody.

Cloning and expression of recombinant human I2PP2A/SET

The I2PP2A/SET gene was cloned into a pET14b plasmid (Novagen Merck KGaA, Darmstadt, Germany). First, the human I2PP2A/SET gene was PCR amplified using the following primers: Forward (N-terminal) Primer: 5'-GGCAGCCATATGTCG-GCGCCGGCGGCCAAAGTC 3'[1] and Reverse (C-terminal) Primer: 5' GTCCTC-GAGGTCATCTTCTCCTTCATCCTCCTC 3'[2]. The PCR product was digested with NdeI and XhoI (New England BioLabs Inc., Ipswich MA, USA) and gel extracted for purification using the QIAquick® Gel Ectraction Kit (QIAGEN Valencia, CA USA). The pET14b vector was first digested with NdeI for 16 h, and then digested with XhoI and gel extracted for purification. 100 ng of digested vector and 150 ng of digested I2PP2A/SET PCR product was used in a ligation reaction, which was incubated at 16°C for 3hr. I2PP2A/SET was verified by direct DNA sequencing at the Nucleic Acid Sequencing Core facility at MUSC. The pET14b/I2PP2A/SET was then transformed into BL21 (DE3) pLysS cells for expression (Novagen Merck KGaA, Darmstadt, Germany), and purification.

I2PP2A/SET expression and purification

BL21 (DE3) pLysS cells transformed with the pET14b/I2PP2A/SET were grown at 37° C with shaking (250 rpm) to an optical density of 0.6 at 600 nm at which time the protein production was induced by adding 0.7 mM IPTG. Cells were then incubated with

shaking (250 rpm) at 25° C for 5 h. For harvesting, cells were centrifuged at 5000 g for 10 min, the pellet was washed once with sodium phosphate buffer (pH 8.0) and resuspended in 25 ml of extraction buffer containing 50 mM sodium phosphate, pH 8.0, 160 mM NaCl, 10 mM imidazole, 1% Triton X-100, 1 mM phenylmethylsulfonyl fluoride, and 1 mM lysozyme. The suspension was chilled on ice for 30 min followed by sonication (8 cycles, 20 sec on, 40 sec off) on ice. The extract was centrifuged at 50,000 g for 30 min at 4 °C and the supernatant was then filtered through a 0.2 mm filter into a 50 ml tube. One ml of nickel-nitrilotriacetic acid agarose (Qiagen, Valencia, CA) was added to the supernatant and the mixture was incubated on ice while shaking at 80 rpm for 1 h. The mixture was poured onto a 10 ml column, and the column was washed first with 20 ml of 50 mM sodium phosphate, pH 8.0 containing 300 mM NaCl and 10 mM imidazole, and subsequently with 15 ml of 50 mM sodium phosphate, pH 8.0 containing 300 mM NaCl and 15 mM imidazole. The column was washed with an additional 10 ml of the same buffer containing 20 mM imidazole. The protein was then eluted in 6 fractions of 0.5 ml in 50 mM sodium phosphate buffer containing 300 mM imidazole. Eluted fractions were then analyzed by 12% SDS-PAGE. Fractions containing I2PP2A/SET were concentrated and desalted in an Ultrafree-15 centrifugal filter device and protein concentrations were determined by the bicinchonic acid method.

Ceramide and FTY720/P-FTY720 measurements by LC/MS/MS

LC/MS/MS analysis of FTY720 and its phosphate (p-FTY720) were performed on a Thermo-Fisher TSQ Quantum triple quadrupole mass spectrometer, operating in a Multiple Reaction Monitoring (MRM) positive ionization mode, using modified version

of a previously published protocol (Bielawski et al, 2010). Simultaneous quantitative analysis of bioactive sphingolipids by high-performance liquid chromatography-tandem mass spectrometry (Bielawski et al, 2010). Optimized mass transitions of 308.2-255.1 at 23eV and 388.3-255.1 at 25eV for FTY720 and p-FTY720 respectively, were monitored and used for quantitative determination.

Plasmids, site-directed mutagenesis, RNAi and shRNA

The mutant forms of I2PP2A/SET were generated using site-directed mutagenesis as described previously (Mukhopadhyay et al, 2009; Senkal et al, 2011). The plasmids for wt-I2PP2A/SET-GFP or its mutant forms, containing I2PP2A/SET- I2PP2A/SET-ER, Y122C or K209D were cloned into pEGFP-C3 vector (BD Biosciences, San Jose, CA) with the GFP tag and transfected into A549 cells by using Effectene (Qiagen) according to manufacturer's protocol. WT-Myc-V5, T58A-Myc-V5, PP2A-HA, WT-RIP1-Flag, DDD-RIP1-Flag, DKD-RIP1-Flag were used as previously described (Mukhopadhyay et al, 2009; Ramnarain et al, 2008).

Scrambled non-targeting control (Dharmacon), PP2Ac (Zhang et al, 2009) or RIPK1, RIPK3 or Drp1 siRNAs (Biton & Ashkenazi, 2011) were transfected into lung cancer cells with Dharmafect for 48h. Stable knockdown of I2PP2A/SET was achieved by retroviral transduction of PLL3.7-shI2PP2A/SET, and cells were then sorted for GFP with flow cytometry (and shRNA control plasmid was also used). To reconstitute WT-I2PP2A/SET in A549/sh-I2PP2A cells, MSCV-flag-I2PP2A/SET was used and puromycin selection was performed (Neviani et al, 2007).

Measurement of cell viability

FTY720 or DMSO control was added to cells for 24 hrs, then cell death was measured by LDH release using the Biovision LDH cytotoxicity detection kit, or Annexin V/7-AAD detection by flow cytometry using the BD Pharmingen™ PE Annexin V Apoptosis Detection Kit, as described by the manufacturers. The IC_{50} values of FTY720 were determined by MTT assay.

Ultra structure analysis by TEM

Cells grown in the absence/presence of FTY720 were fixed in 2% glutyraldehyde in 0.1M cacodylate buffer after removal of culture medium. After post-fixation in 2% osmium tetroxide, specimens were embedded in epon 812, and sections were cut orthogonally to the cell monolayer with a diamond knife. Thin sections were visualized in a JEOL 1010 transmission electron microscope.

Animal studies, IHC and human NSCLC primary tumors

Six to eight week old mice SCID mice (Harlan) were implanted with 2 million A549 cells (sh-control, sh-I2PP2A/SET, sh-I2PP2A/SET/WT-I2PP2A/SET) on the flanks (6-8 mice per group, 12-16 tumors). After measureable implantation of the tumor, mice were divided into two groups (placebo control) or FTY720 (3 mg/kg) and were treated daily through oral gavage. Tumors were measured with calipers every 3 days. At the end of study (15-18 days), blood and tumors were collected from the mice. FTY720 and P-FTY720 was measured in the tumor and serum at the MUSC Lipidomics facility. Serum LDH was measured by Analytics Inc. Six to eight week old WT (C57B7/6) and Sphingosine Kinase 2 knockout (SK2-/-) mice (MUSC) were implanted using Lewis

Lung Carcinoma (LLC) cells (1 x 10^6). Mice were treated orally with placebo (water) or FTY720 dissolved in H_20 (10 mg/kg). These studies were conducted according to the IACUC approved protocols at MUSC.

Fresh frozen human lung tumors and adjacent non-cancerous lung tissues were obtained from the Tumor Bank at Hollings Cancer Center. Studies using these human samples were performed according to the protocols approved by the IRB at MUSC. TMAs (LC1002) containing NSCLC tumor/normal tissues were obtained from BioMax, Inc. IHCs were performed using anti-I2PP2A/SET (Santa Cruz Bio. H-120, sc-25564), anti-PP2A (Epitomics), anti-P-PP2A Y307 (Epitomics), or anti-c-Myc (Santa Cruz Bio. 9E10, sc-40). Statistical analyses were performed as described previously (Mukhopadhyay et al, 2009; Saddoughi et al).

Statistical analysis

Statistical analysis was performed by Student's t-test using Prism/GraphPad software; $p < 0.05$ was considered significant, as described previously (Salas et al, 2011; Saddoughi et al, 2011).

SUPPLEMENTARY RESULTS

Supplementary Figure Legends

Figure S1. Molecular modeling of ceramide-binding pocket of I2PP2A/SET Interaction diagram obtained by molecular modeling/simulation of C_{18}-ceramide-I2PP2A/SET binding suggests that one of the prominent docking sites of I2PP2A/SET for ceramide binding includes the K209 residue, which interacts with the primary hydroxyl group of ceramide possibly via charge attraction.

Figure S2. Endogenous ceramides bind to I2PP2A/SET. The binding of WT-, K209D, Y122C-, I2-ER to endogenous ceramides in A549 cells was measured by LC/MS/MS. I2PP2A/SET-wt, K209D and I2-ER containing GFP tag were overexpressed in A549 cells, pulled down by immunoprecipitation by anti-GFP-conjugated columns. The elution and flow through fractions were analyzed for endogenous ceramides by LC/MS/MS. Data were compared to vector-GFP controls. Ceramide levels were normalized to Pi.

Figure S3. Measurement of WT- and mutant I2PP2A/SET expression in A549 cells. (A-C) Expression of WT- (A), K209D- (A), ER- (B) and Y122C-I2PP2A/SET-GFP (C) in A549 cells were measured by Western blotting using anti-GFP antibody compared to vector-transfected controls. Actin was used as a loading control.

Figure S4. Effects of subcellular localization of ceramide on I2PP2A binding. (A) Endogenous ceramides with different fatty acid chain lengths were measured using LC/MS/MS in A549 cell extracts enriched in cytoplasmic and nuclear fractions.

Ceramide levels were normalized to Pi. Error bars represent s.d. (**B**) Effects of WT-, K209D- or ER-I2PP2A/SET-GFP on B-C_6-ceramide binding in cell extracts in vitro were examined by Western blotting after avidin column pulldown studies. Chemical structure of B-C_6-ceramide is shown.

Figure S5. Measurement of ceramides in lung tumor versus non-cancerous lung tissues. (A-B) Endogenous ceramides with different fatty acid chain lengths were measured using LC/MS/MS, and normalized to ng protein (A). Ceramide levels (pmol/ng protein) were determined in duplicates, n=10 (B). Error bars represent s.d. (**P <0.05).

Figure S6. Inactive form of PP2A (P-PP2A) is over-expressed in lung tumor tissues compared to non-cancerous lung tissues. (**A**) Expression of p-PP2A (Y307) in lung tumor tissues versus adjacent non-cancerous lung tissues were detected by IHC using TMA (n=48 pairs). (**B**) Relative expression of p-PP2A (Y307) in lung tumor versus normal lung tissues are shown, calculated using paired t-test.

Figure S7. Molecular modeling/simulation of FTY720-I2PP2A/SET binding. Interaction diagram obtained by molecular modeling/simulation of FTY720-I2PP2A/SET binding, as constructed based on ceramide-I2PP2A/SET interaction, predicts that one of the primary hydroxyl groups of FTY720 binds to the K209 residue of I2PP2A/SET.

Figure S8. Effects of FTY720 on c-Myc expression and hTERT mRNA in A549-xenografts in SCID mice. (**A-B**) Effects of FTY720 on c-Myc expression (A) or hTERT

mRNA (B) were measured by Western blotting or Q-PCR, respectively in A549-xenograft-derived tumor tissues in SCID mice, compared to controls. Error bars represent s.d. and $p < 0.05$ was considered significant. (**C**) Total ceramide levels were measured by LC/MS/MS in tumor and serum (left and right panels, respectively) of SCID mice treated with vehicle control or FTY720 (3mg/kg).

Figure S9. Genetic loss or siRNA-mediated knockdown of SK-2 does not prevent FTY720-mediated growth inhibition. (A) Effects of FTY720 (10 μM) on the growth of MEFs obtained from WT, SK1-/- and SK2-/- mice were measured by MTT assays. (**B-C**) Effects of shRNA-mediated stable knockdown of SK-2 on SK-2 mRNA (B) and FTY720-mediated cell death were measured by Q-PCR and LDH release assay (C), respectively. Error bars represent s.d. and $P < 0.05$ was considered significant.

Figure S10. Detection of PP2A-HA expression or reconstitution of WT- and mutant-I2PP2A/SET in A549/sh-I2PP2A/SET cells. (A-B) Expression of PP2A-HA (A), or WT-, K209D-, Y122C-, and ER-I2PP2A/SET-GFP (B) in A549/sh-I2PP2A/SET cells were determined by Western blotting using the anti-HA or anti-GFP antibodies, respectively, compared to vector-only-transfected controls. Actin was used as a loading control.

Figure S11. Effects of FTY720 versus taxol on cell death. (A) Effects of FTY720 on A549 cell death were measured using the PE Annexin V Apoptosis Detection Kit by flow cytometry as described by the manufacturer. Increases in A549 cell populations with 7-

AAD and/or Annexin V/7-AAD positive staining in response to FTY720 compared to controls are shown. Error bars represent s.d. (**B**) Expression of I2PP2A/SET in MEFs (wt and ATG5-/-) versus A549 cells in the absence/presence of taxol were examined by Western blotting using anti-I2PP2A/SET antibody. (**C**) Effects of taxol on apoptosis were measured by caspase 3activity assay in wt versus ATG5-/- MEFs. Error bars represent s.d.

Figure S12. Effects of FTY720 on cell death in human H157 and H827 lung cancer cells. (A-C) Effects of FTY720 (20 μM) on cell death in H157, expressing a mutant K-Ras, in the absence/presence of necrostatin (A) or OA (B), or in H827, expressing mutant EGFR (C) in the absence/presence of siRNAs against RIPK1 or PP2A, were measured using LDH release assay. Error bars represent s.d. (***P* <0.05).

Figure S13. Role of PP2Ac, RIPK3 or DRP1 in FTY720-mediated cell death. (A) Effects of ectopic expression of PP2Ac on FTY720-mediated cell death was measured by detection of serum LDH release in RIPK1-/- MEFs. Error bars represent s.d. and *P* <0.05 was considered significant. (**B-D**) Effects of siRNA-mediated knockdown of RIPK3 or Drp1 on RIPK1 (B) or Drp1 (C) expression and A549 cell death were measured by Western blotting and LDH release assay (D), respectively. Actin levels were used as loading controls in (B) and (C). Error bars represent s.d. (**P* <0.05) in (D).

Figure S14. Measurement of WT-, DDD-, DKD-RIPK1-FLAG expression. (A) Expression of WT-, DDD-, and DKD-RIPK1-FLAG was confirmed by Western blotting

using anti-RIPK1 antibody compared to vector-transfected controls (A), lanes 2-4 and 1, respectively. **(B)** Because the anti-RIPK1 antibody recognizes the death domain, the DDD-RIPK1 was not detectable when it was used in Western blotting (in A, lane 3). Therefore, expression of the DDD-RIPK1 was detected using the anti-FLAG antibody in Western blots compared to vector-transfected controls, lanes 2-1, respectively.

Figure S15. Full blots. Western blots shown in Figures 2, 3 and 5 are shown.

Figure S16. Full blots. Western blots shown in Figures 6 and 8 are shown.

Figure S1

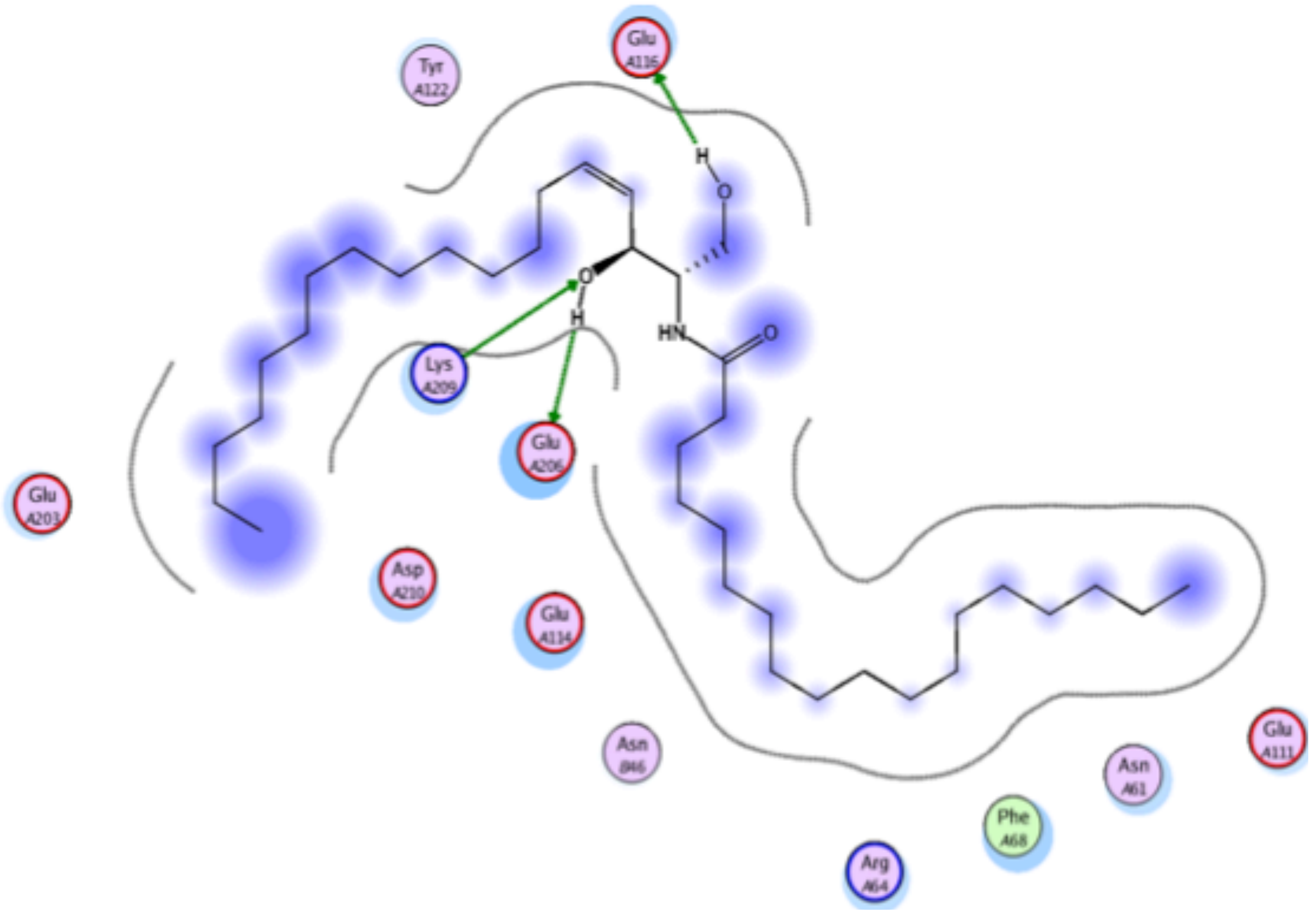

Glu A116
Tyr A122
H
O
O
H
HN
O
Lys A209
Glu A206
Glu A203
Asp A210
Glu A114
Asn B46
Glu A111
Asn A61
Phe A68
Arg A64

Figure S2

Ceramide	Protein	Flow Through (pmol/ nmolPi)	Elution (pmol/ nmol Pi)	Total =E+FT	% of elution= (E/T)*100	Total Binding (I2PPA - Vector)
**C14**	vector	0.070	0.006	0.076	7.9	
	I2PP2A	0.071	0.004	0.075	4.7	no binding
	I2-K209D	0.065	0.011	0.076	14.5	6.6
	I2-ER	0.085	0.008	0.093	8.6	0.7
	I2-Y122C	0.084	0.006	0.090	6.6	no binding
**C16**	vector	1.300	0.070	1.370	5.1	
	I2PP2A	1.310	0.036	1.346	2.7	no binding
	I2-K209D	1.060	0.044	1.104	4.0	no binding
	I2-ER	1.000	0.140	1.140	12.3	7.2
	I2-Y122C	0.980	0.057	1.037	5.5	0.4
**C18**	vector	0.091	0.038	0.129	29.5	
	I2PP2A	0.053	0.100	0.153	65.4	35.9
	I2-K209D	0.077	0.038	0.115	33.0	3.5
	I2-ER	0.086	0.045	0.131	34.4	4.9
	I2-Y122C	0.050	0.133	0.183	72.7	43.2
**C20**	vector	0.133	0.014	0.147	9.5	
	I2PP2A	0.193	0.043	0.236	18.2	8.7
	I2-K209D	0.203	0.025	0.228	11.0	1.5
	I2-ER	0.166	0.027	0.193	14.0	4.5
	I2-Y122C	0.100	0.066	0.166	39.8	30.3
**C22**	vector	0.190	0.006	0.196	3.1	
	I2PP2A	0.210	0.019	0.229	8.3	5.2
	I2-K209D	0.210	0.006	0.216	2.8	no binding
	I2-ER	0.210	0.009	0.219	4.1	1.0
	I2-Y122C	0.183	0.031	0.214	14.3	11.2
				0.000		
**C24**	vector	3.130	0.081	3.211	2.5	
	I2PP2A	4.310	0.240	4.550	5.3	2.8
	I2-K209D	4.910	0.143	5.053	2.8	0.3
	I2-ER	4.800	0.121	4.921	2.5	no binding
	I2-Y122C	4.200	0.363	4.563	8.0	5.5
**C26**	vector	0.103	0.005	0.108	4.6	
	I2PP2A	0.113	0.015	0.128	11.7	7.1
	I2-K209D	0.130	0.005	0.135	3.7	no binding
	I2-ER	0.113	0.006	0.119	5.0	0.4
	I2-Y122C	0.120	0.015	0.135	10.9	6.3

Figure S3

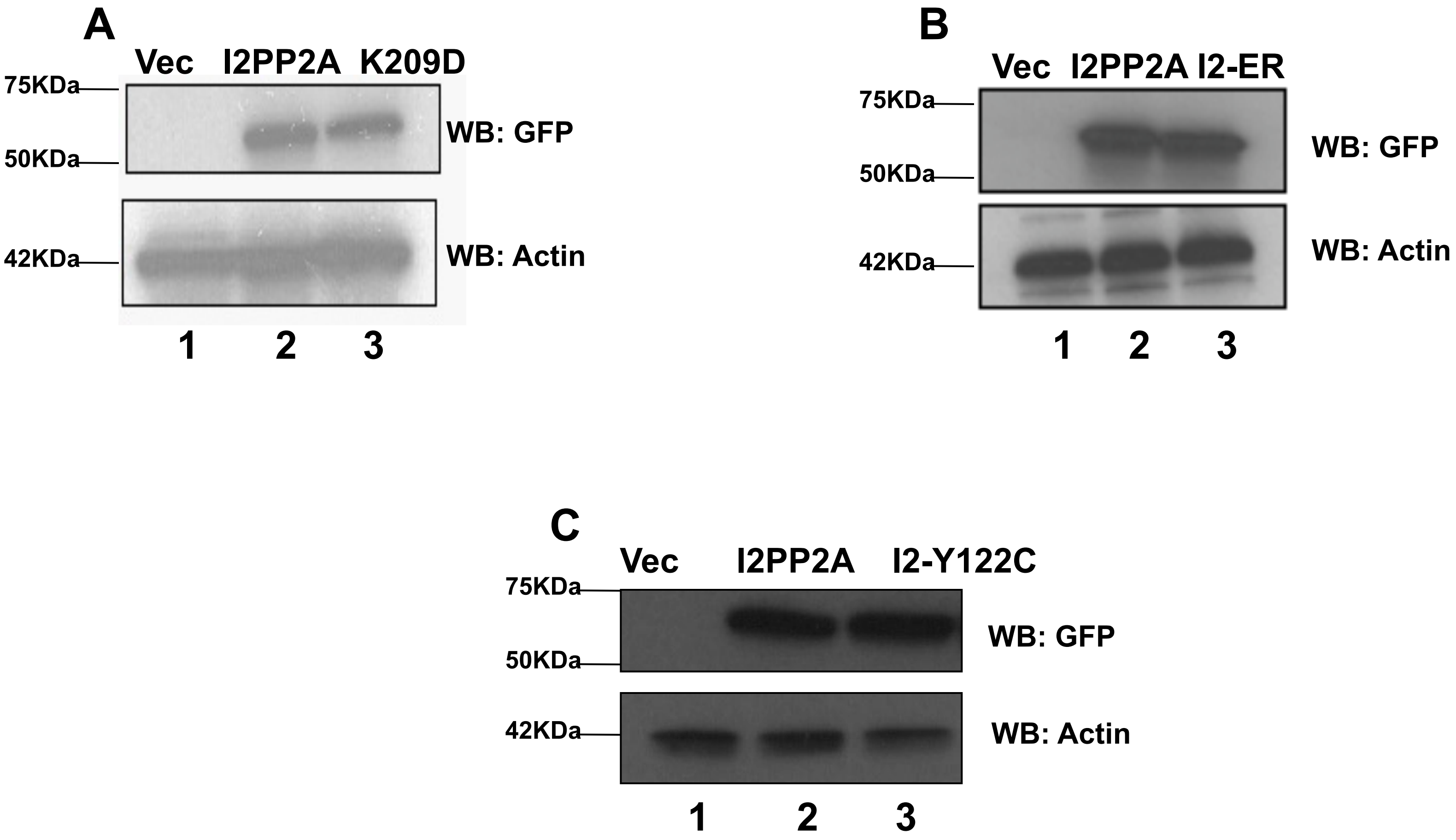
A
Vec
I2PP2A
K209D
75KDa
50KDa
42KDa
WB: GFP
WB: Actin
1
2
3
B
Vec
I2PP2A
I2-ER
75KDa
50KDa
42KDa
WB: GFP
WB: Actin
1
2
3
C
Vec
I2PP2A
I2-Y122C
75KDa
50KDa
42KDa
WB: GFP
WB: Actin
1
2
3

Figure S4

A

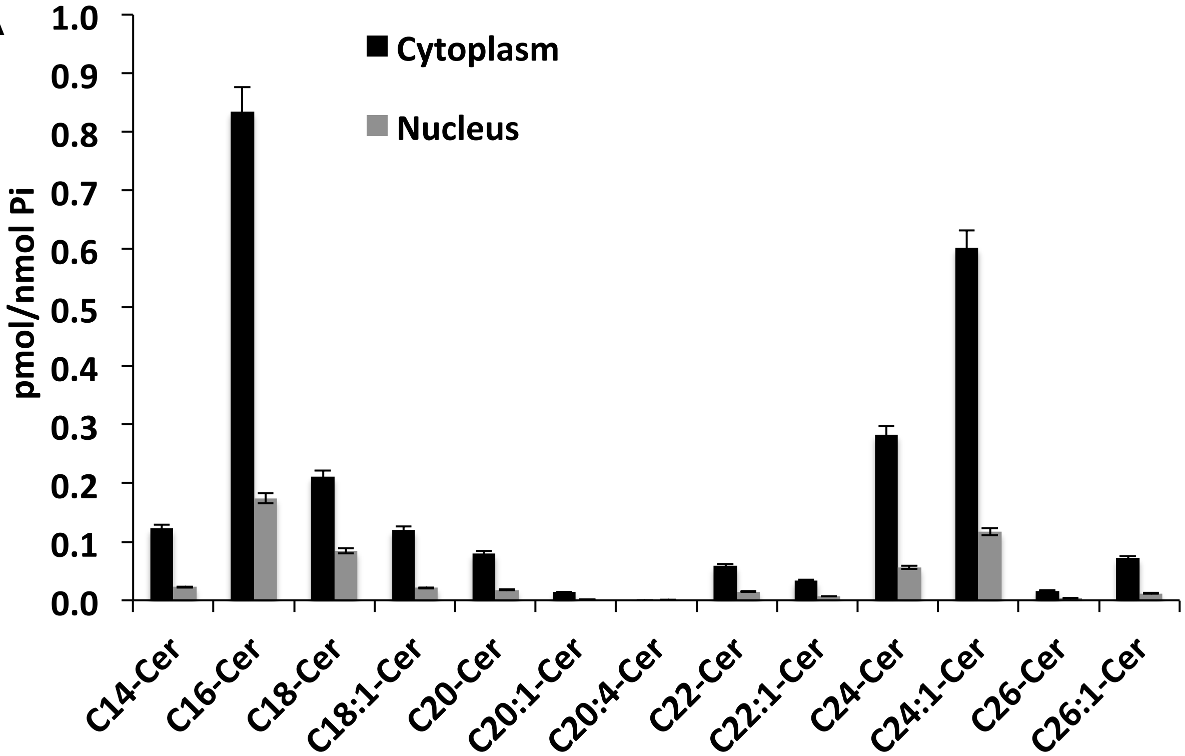

B

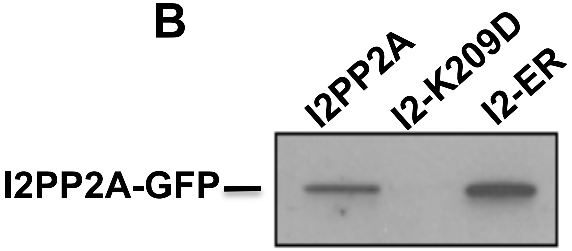

In-vitro binding

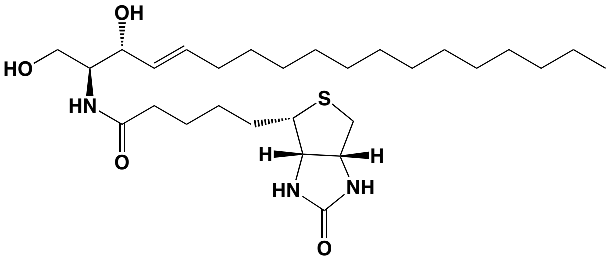

Biotin-C6-ceramide

Figure S5

A

	Normal pmol/ng protein (n=10)	Tumor pmol/ng protein (n=10)
C14-ceramide	1.62	2.19
C16-ceramide	13.88	25.43
C18-ceramide	4.78	2.73
C18:1-ceramide	2.03	1.47
C20-ceramide	3.68	2.63
C20:1-ceramide	0.78	0.64
C20:4-ceramide	0.06	0.09
C22-ceramide	2.36	1.87
C24-ceramide	14.61	11.72
C24:1-ceramide	18.47	17.90
C26-ceramide	0.58	0.67
C26:1-ceramide	0.69	0.60

B

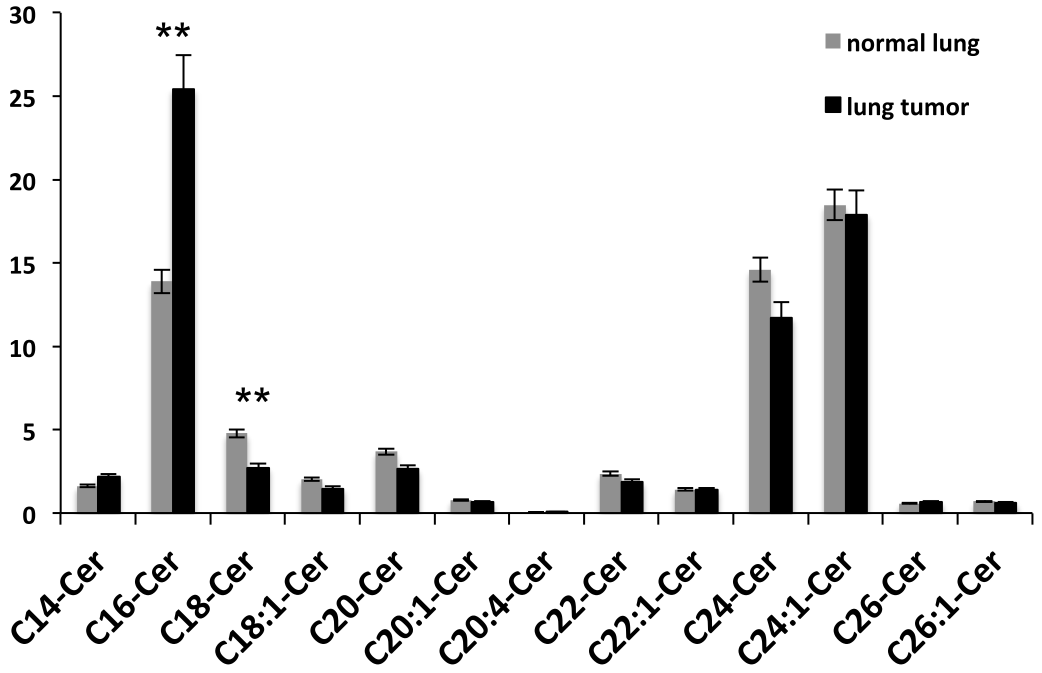

Figure S6

A

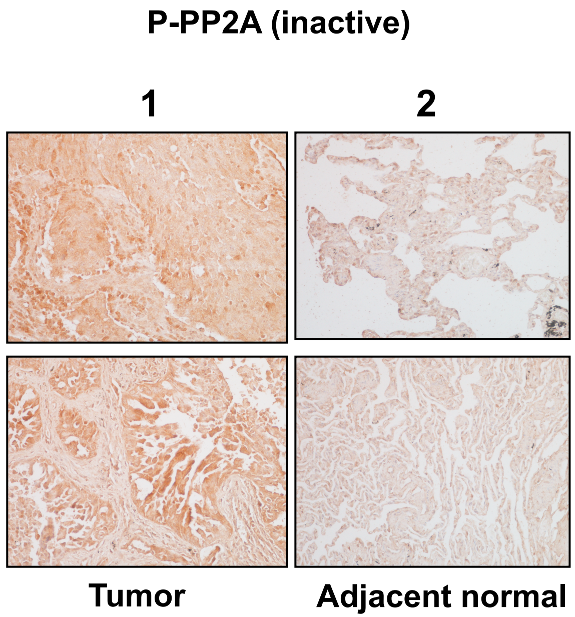
P-PP2A (inactive)
1
2
Tumor
Adjacent normal

B

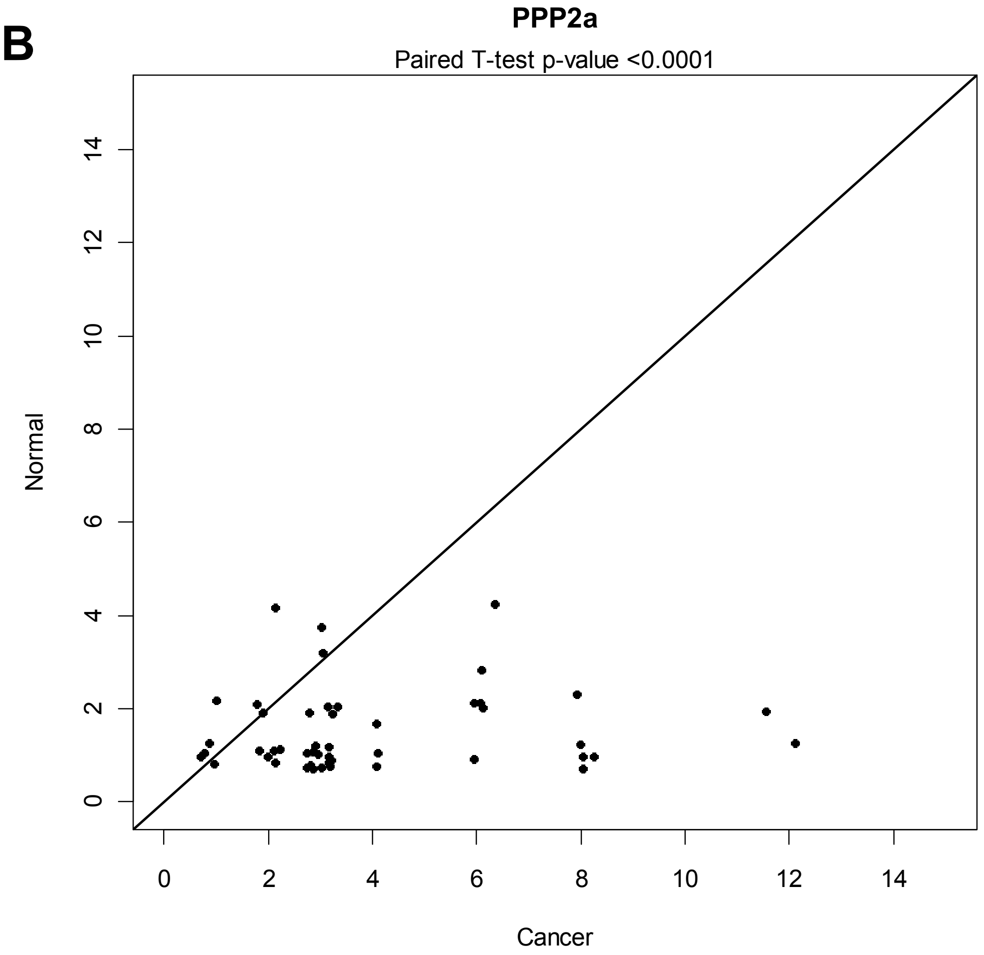
PPP2a
Paired T-test p-value <0.0001
Normal
Cancer
0
2
4
6
8
10
12
14

Figure S7

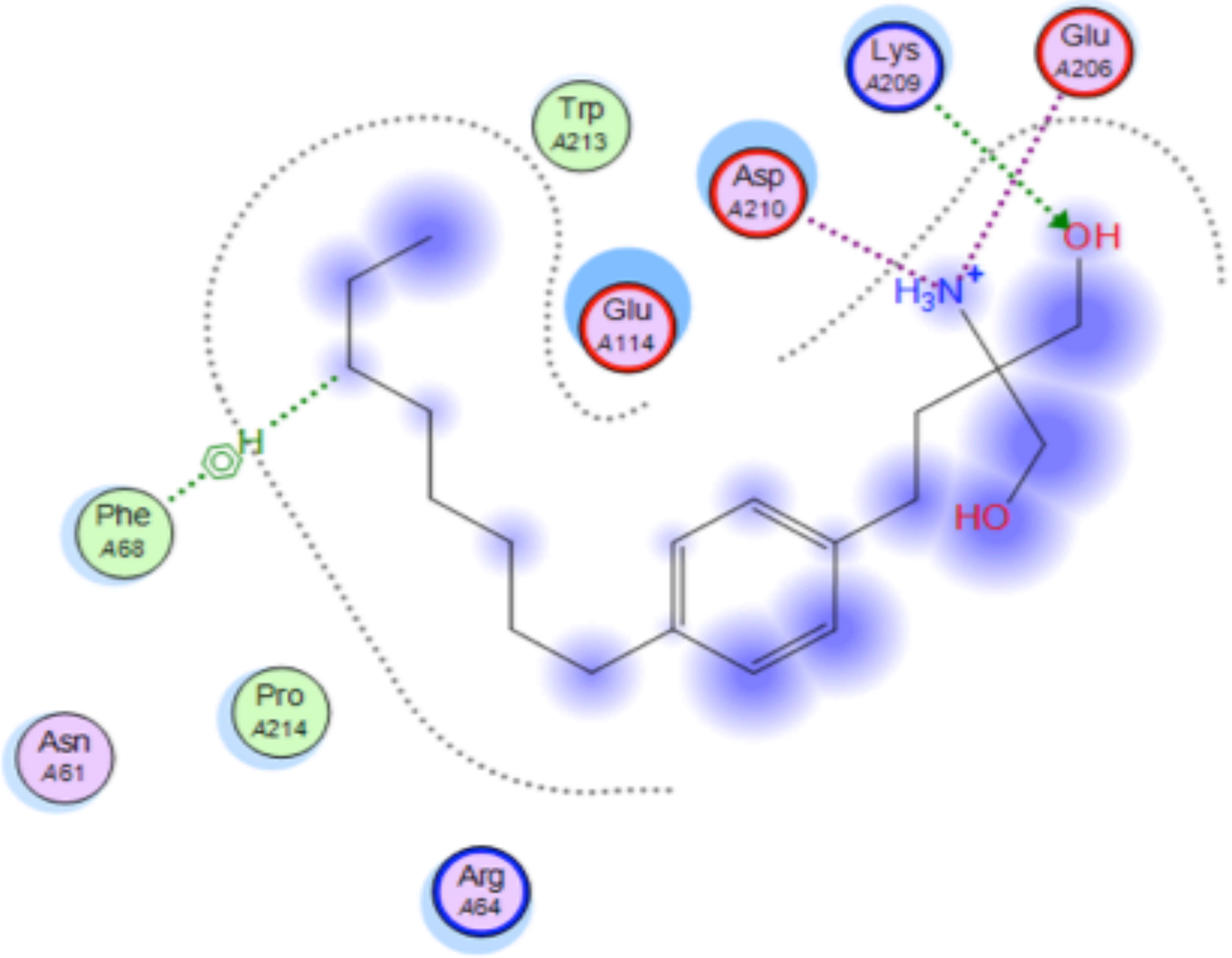

Trp
A213
Lys
A209
Glu
A206
Asp
A210
OH
Glu
A114
H_3N^+
H
Phe
A68
HO
Pro
A214
Asn
A61
Arg
A64

Figure S8

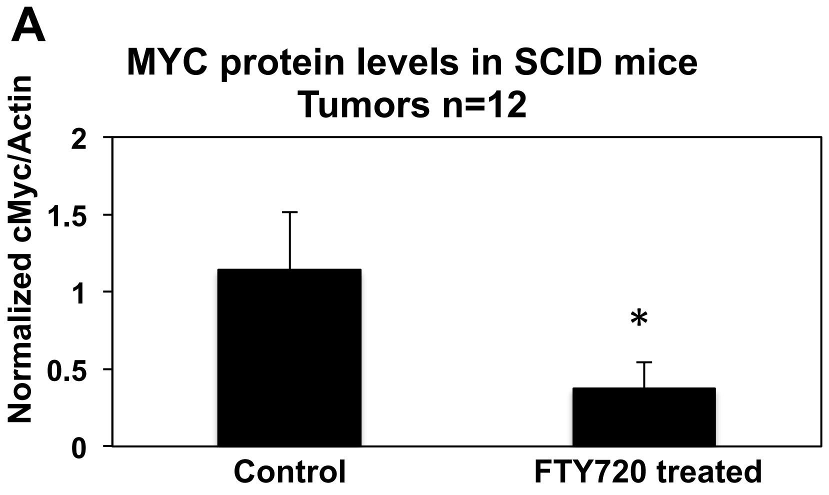
A
MYC protein levels in SCID mice
Tumors n=12
Normalized cMyc/Actin
2
1.5
1
0.5
0
*
Control
FTY720 treated

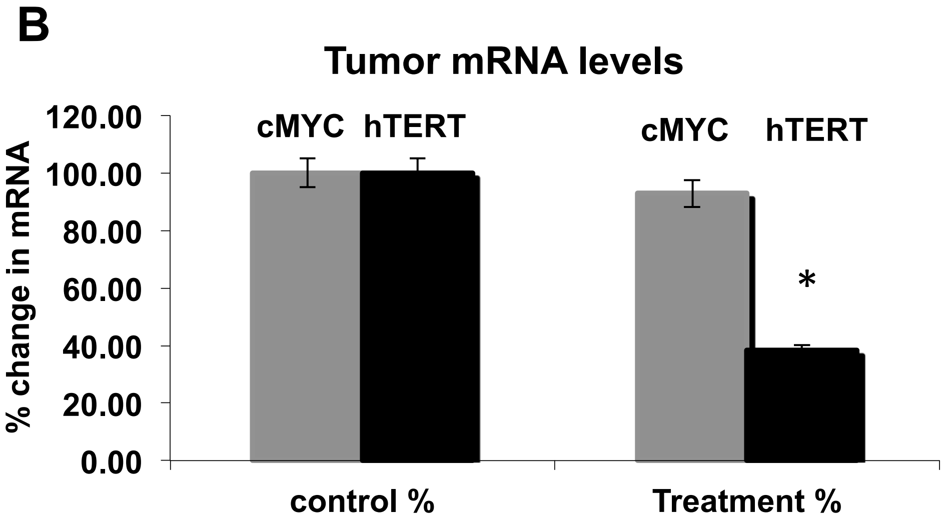
B
Tumor mRNA levels
% change in mRNA
120.00
100.00
80.00
60.00
40.00
20.00
0.00
cMYC
hTERT
cMYC
hTERT
*
control %
Treatment %

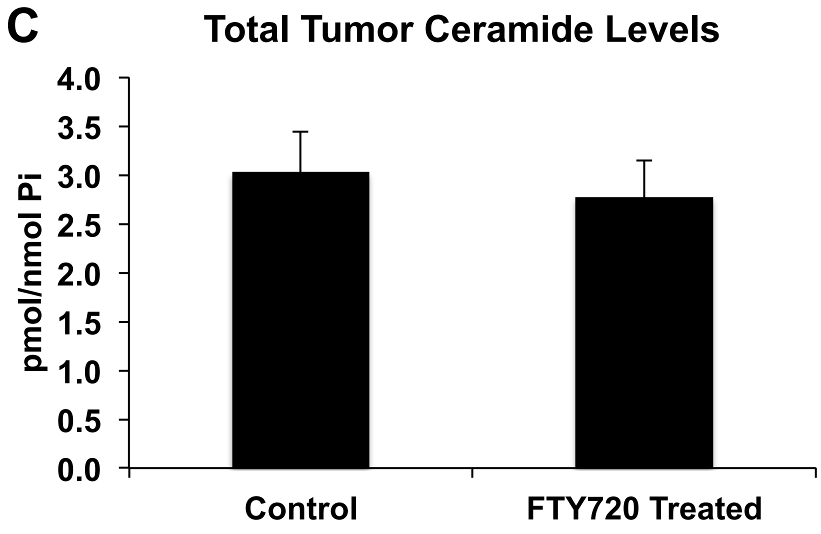
C
Total Tumor Ceramide Levels
pmol/nmol Pi
4.0
3.5
3.0
2.5
2.0
1.5
1.0
0.5
0.0
Control
FTY720 Treated

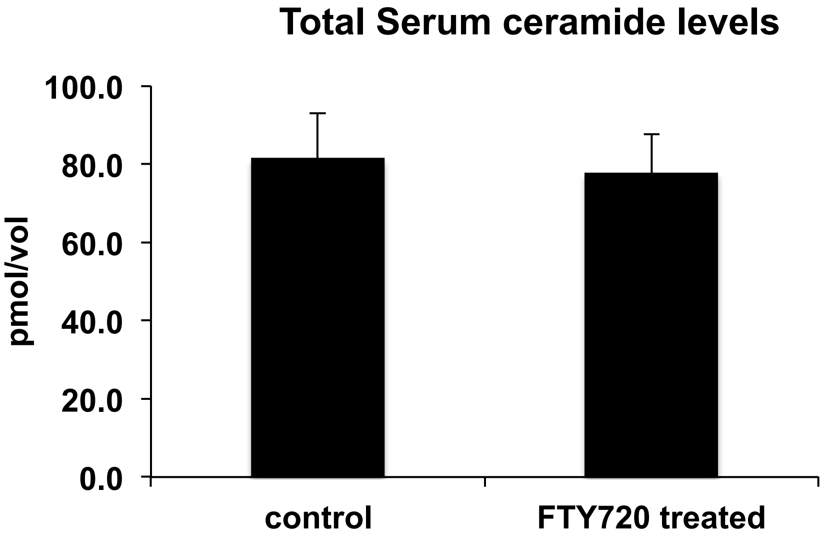
Total Serum ceramide levels
pmol/vol
100.0
80.0
60.0
40.0
20.0
0.0
control
FTY720 treated

Figure S9

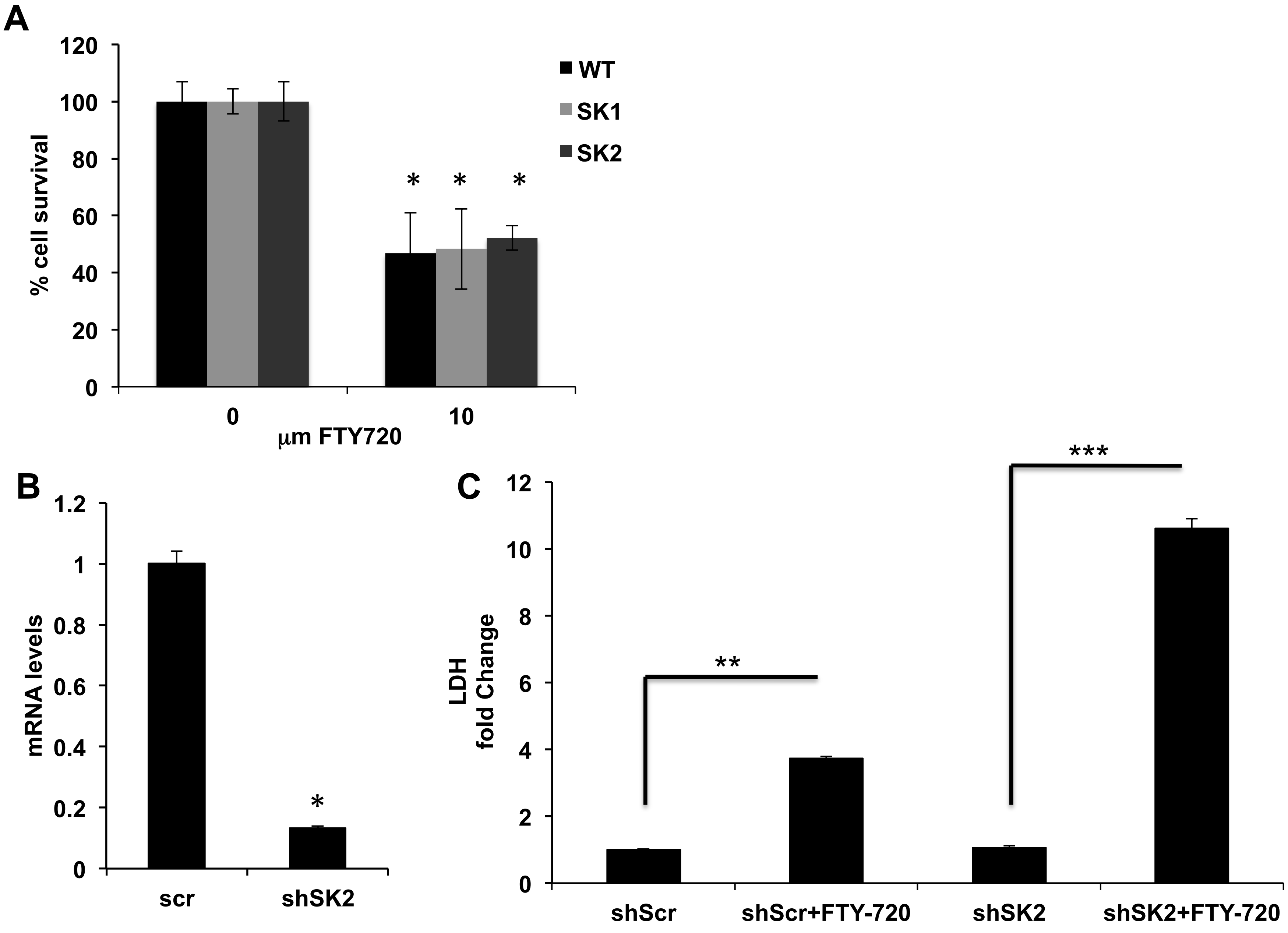
A
120
100
80
60
40
20
0
% cell survival
WT
SK1
SK2
*
*
*
0
10
μm FTY720
B
1.2
1
0.8
0.6
0.4
0.2
0
mRNA levels
*
scr
shSK2
C
12
10
8
6
4
2
0
LDH
fold Change
**
***
shScr
shScr+FTY-720
shSK2
shSK2+FTY-720

Figure S10

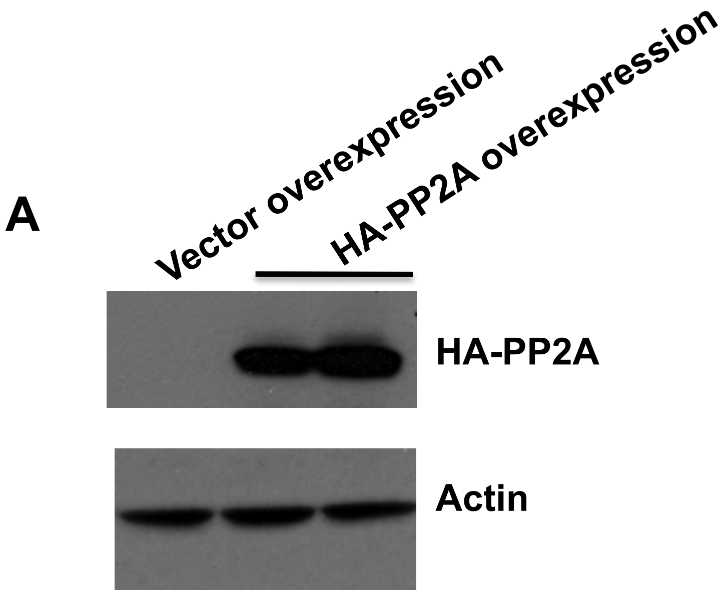
A
Vector overexpression
HA-PP2A overexpression
HA-PP2A
Actin

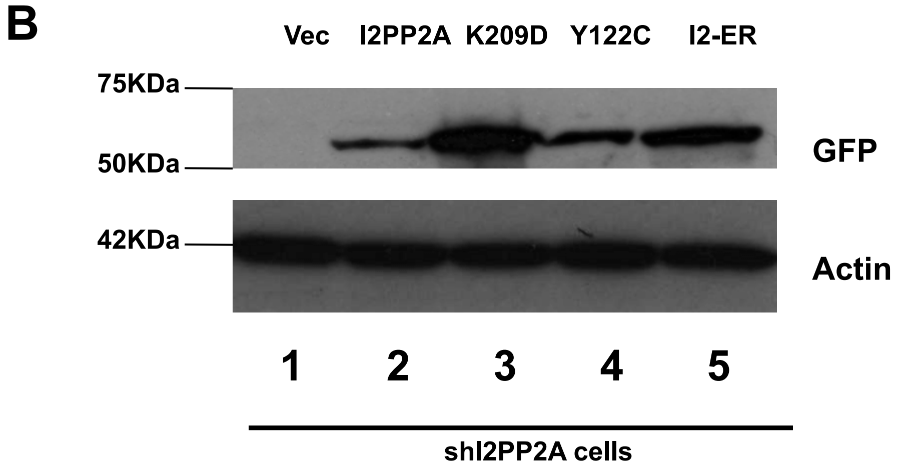
B
Vec
I2PP2A
K209D
Y122C
I2-ER
75KDa
50KDa
42KDa
GFP
Actin
1
2
3
4
5
shI2PP2A cells

Figure S11

A

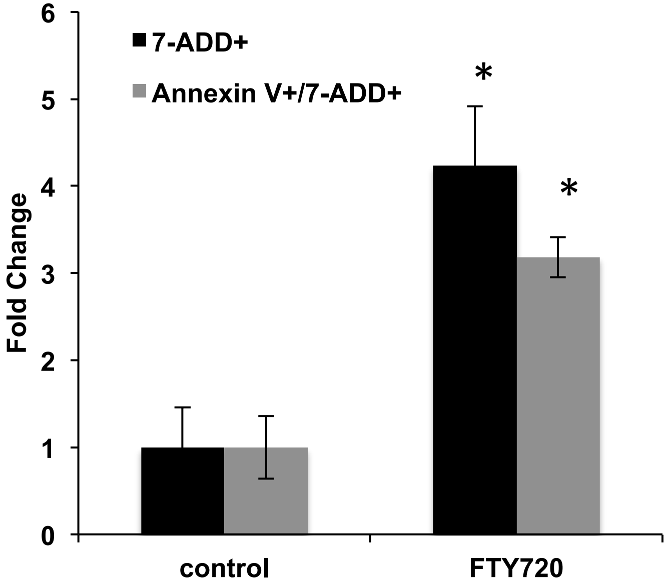

C

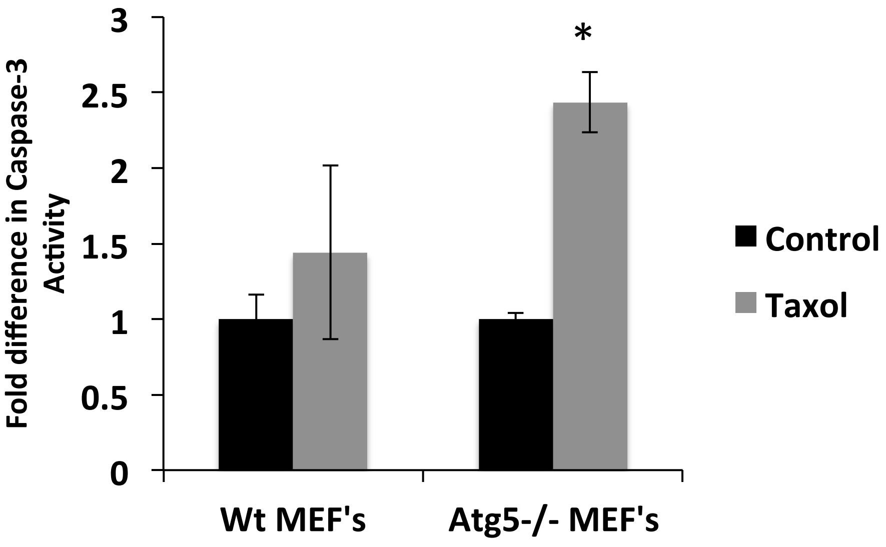

B

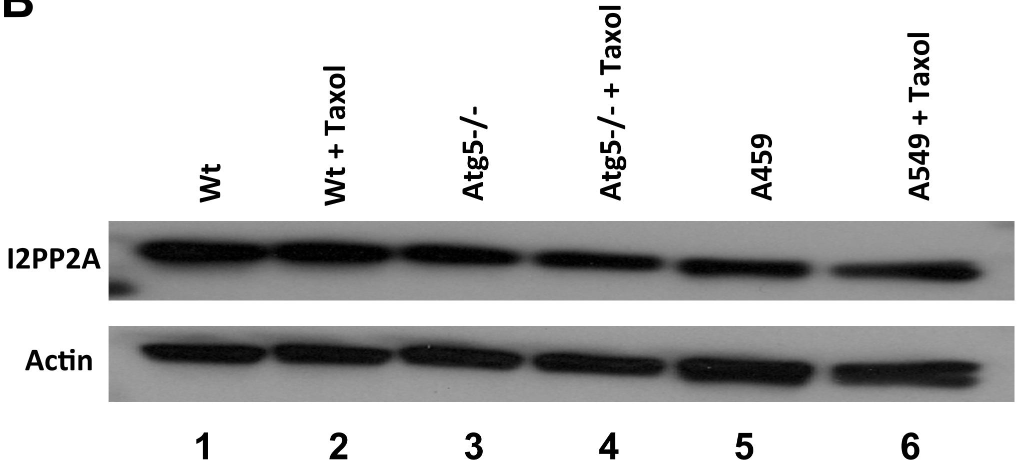

Figure S12

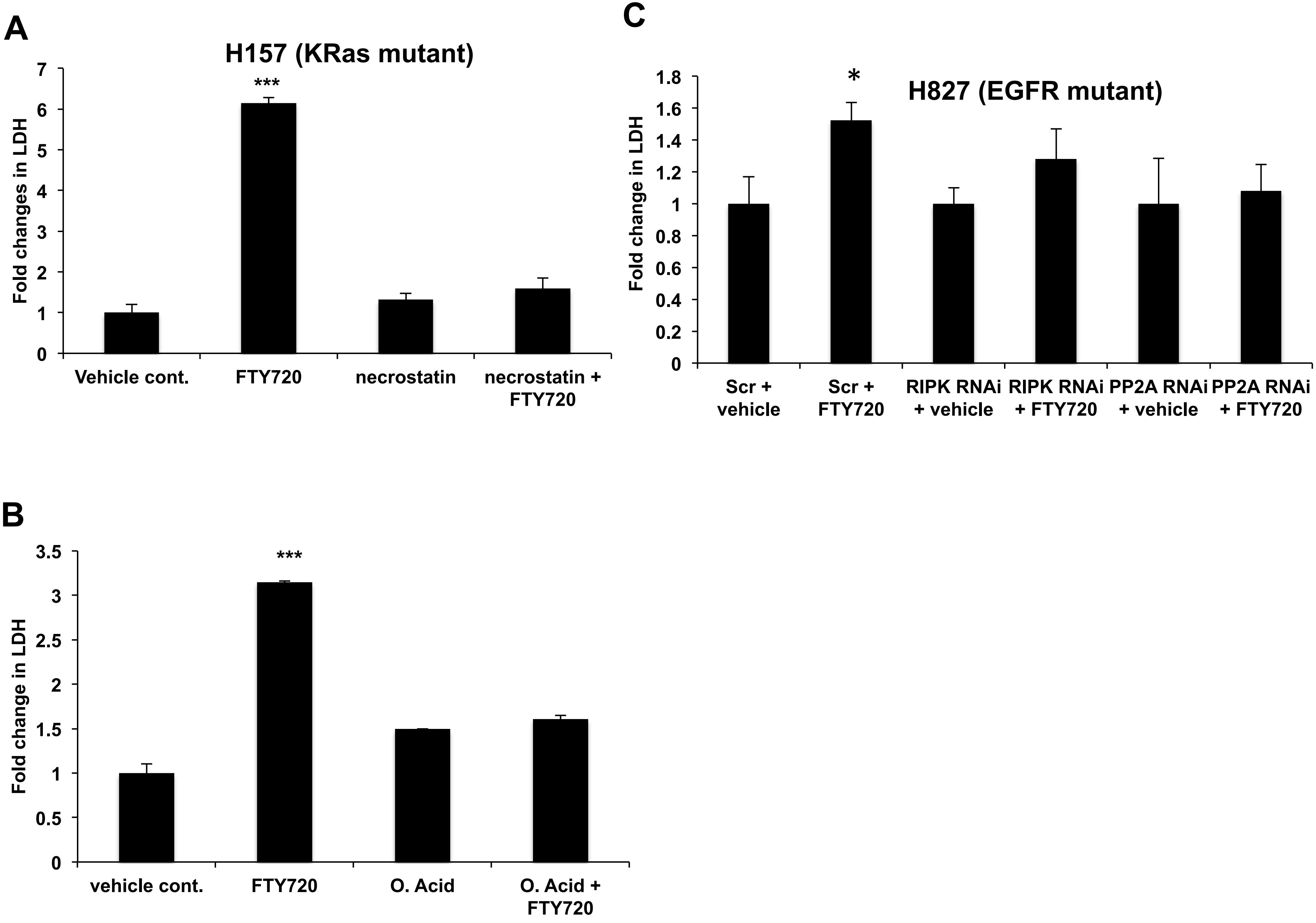
A
H157 (KRas mutant)
Fold changes in LDH
0
1
2
3
4
5
6
7
***
Vehicle cont.
FTY720
necrostatin
necrostatin + FTY720
B
Fold change in LDH
0
0.5
1
1.5
2
2.5
3
3.5
***
vehicle cont.
FTY720
O. Acid
O. Acid + FTY720
C
H827 (EGFR mutant)
Fold change in LDH
0
0.2
0.4
0.6
0.8
1
1.2
1.4
1.6
1.8
*
Scr + vehicle
Scr + FTY720
RIPK RNAi + vehicle
RIPK RNAi + FTY720
PP2A RNAi + vehicle
PP2A RNAi + FTY720

Figure S13

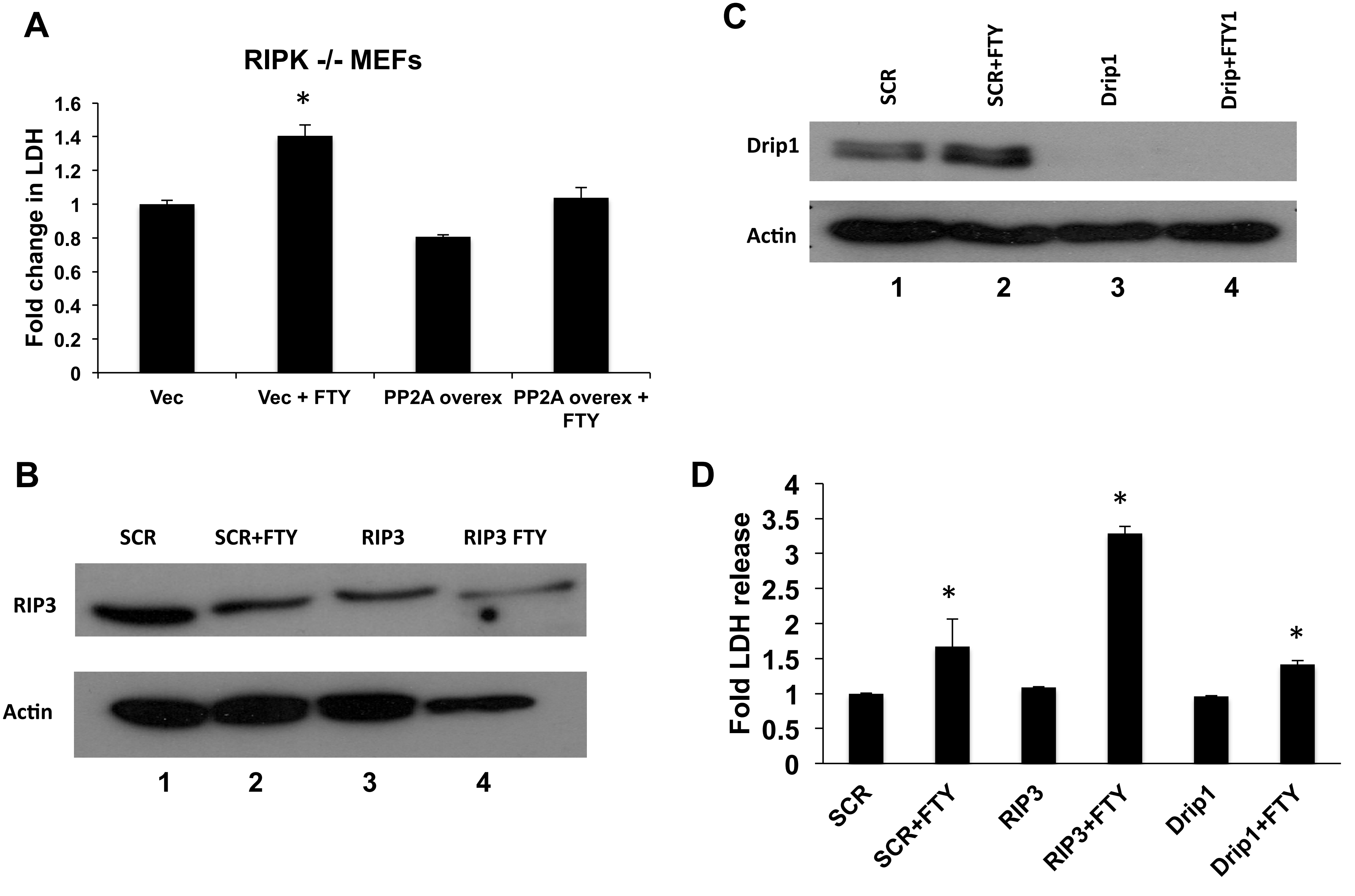

A
RIPK -/- MEFs
Fold change in LDH
1.6
1.4
1.2
1
0.8
0.6
0.4
0.2
0
*
Vec
Vec + FTY
PP2A overex
PP2A overex + FTY
B
SCR
SCR+FTY
RIP3
RIP3 FTY
RIP3
Actin
1
2
3
4
C
SCR
SCR+FTY
Drip1
Drip+FTY1
Drip1
Actin
1
2
3
4
D
Fold LDH release
4
3.5
3
2.5
2
1.5
1
0.5
0
*
*
*
SCR
SCR+FTY
RIP3
RIP3+FTY
Drip1
Drip1+FTY

Figure S14

A

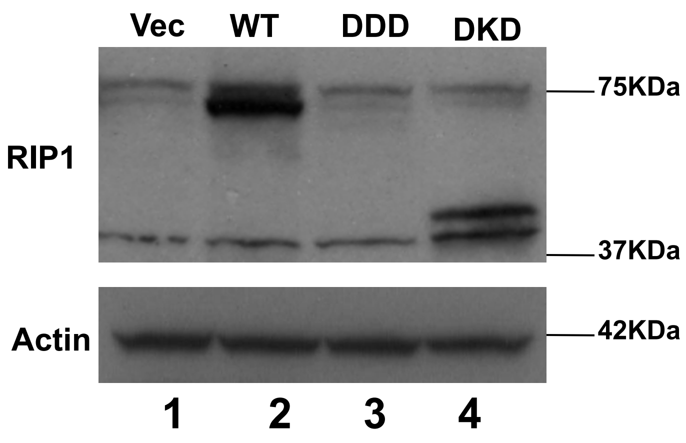

B

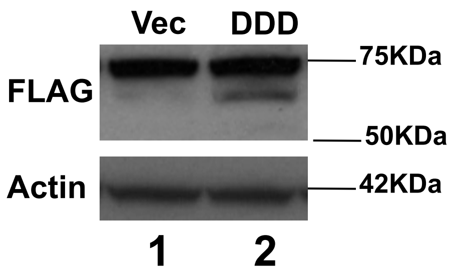

Figure S15

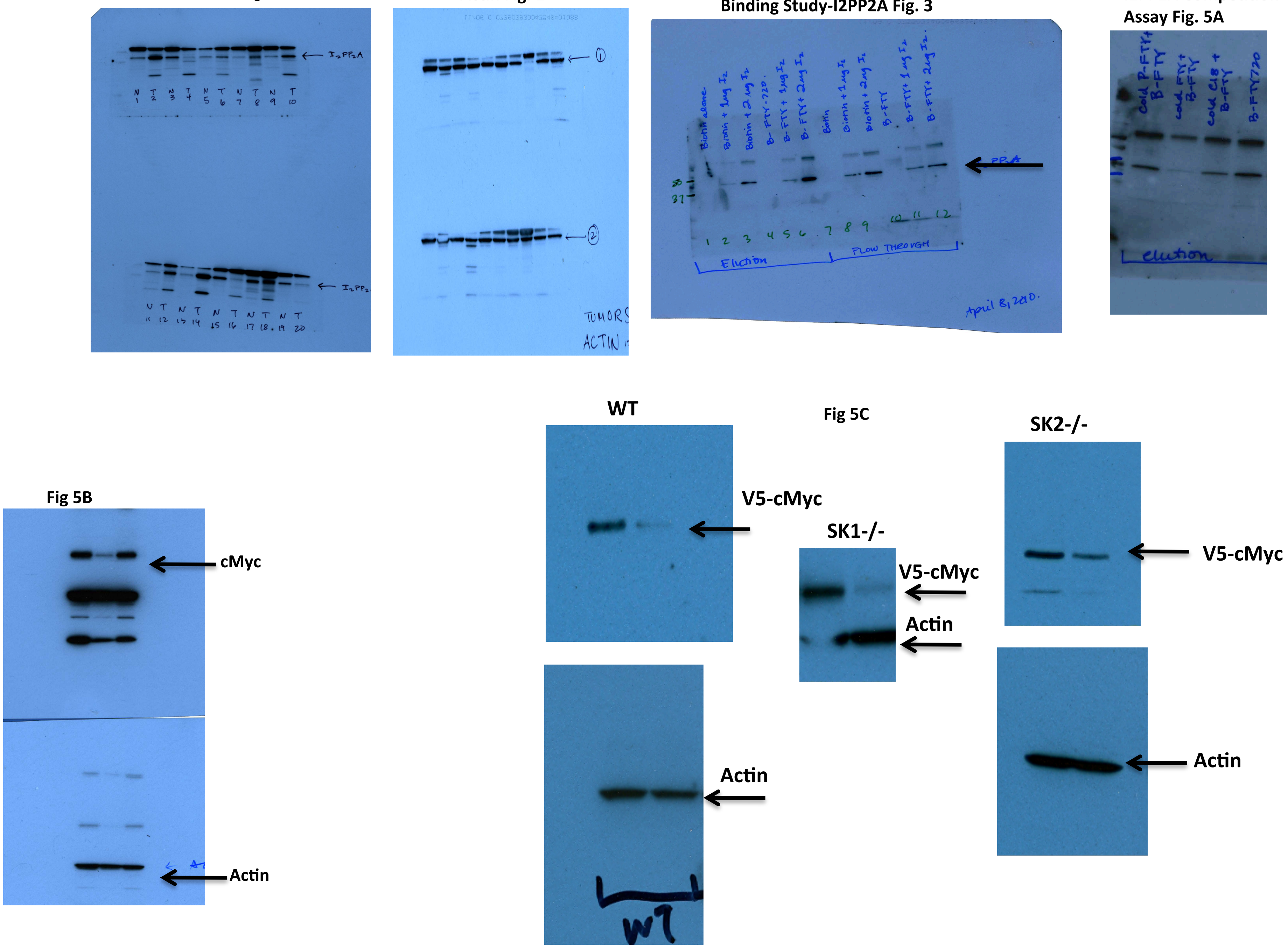
I2PP2A Fig. 2
Actin Fig. 2
Binding Study-I2PP2A Fig. 3
I2PP2A Competition Assay Fig. 5A
Fig 5B
cMyc
Actin
WT
Fig 5C
V5-cMyc
Actin
SK1-/-
V5-cMyc
Actin
SK2-/-
V5-cMyc
Actin

Figure S16

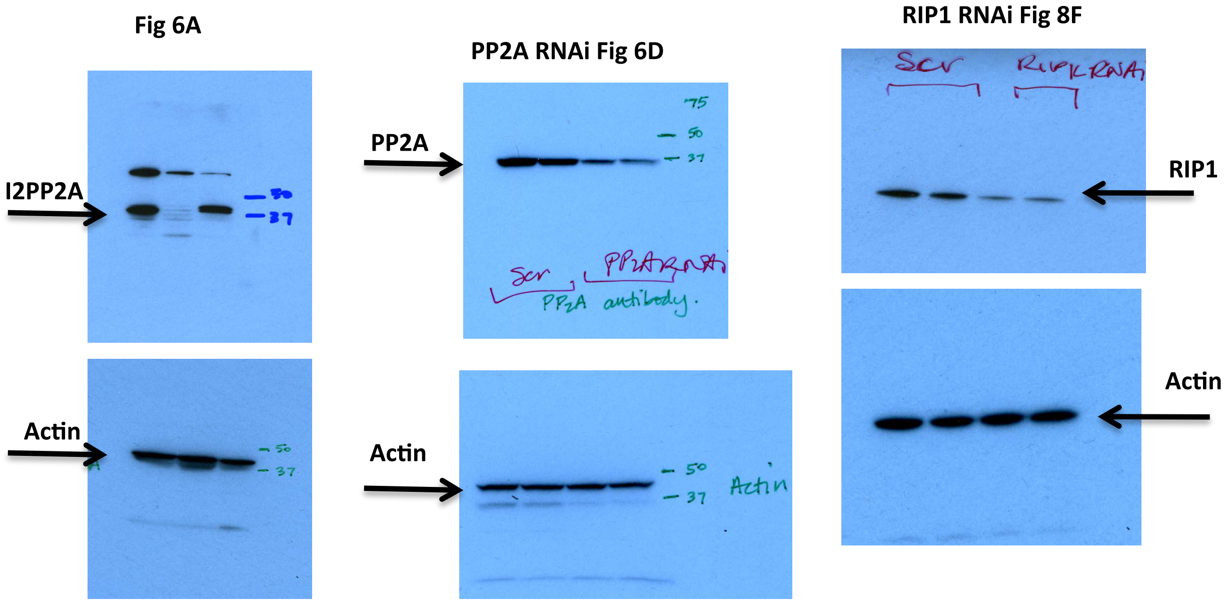
Fig 6A
PP2A RNAi Fig 6D
RIP1 RNAi Fig 8F
I2PP2A
PP2A
RIP1
Actin
Actin
Actin
Scr
PP2A antibody.
50
37
75
